# Annual versus less frequent mammographic surveillance in people with breast cancer aged 50 years and older in the UK (Mammo-50): cost-effectiveness and budget impact analysis

**DOI:** 10.1038/s41416-025-03248-2

**Published:** 2025-11-06

**Authors:** Paola Cocco, Chris Bojke, Claire Hulme, Peter S. Hall, Amy Hopkins, Andrea Marshall, Janet A. Dunn, David Meads, Bethany Shinkins

**Affiliations:** 1 Academic Unit of Health Economics, University of Leeds, Leeds, UK; 2 Lumanity, Sheffield, UK; 3 University of Exeter, Exeter, UK; 4 University of Edinburgh, Edinburgh, UK; 5 Warwick Clinical Trials Unit, University of Warwick, Coventry, UK; 6 Warwick Screening, University of Warwick, Coventry, UK

**Keywords:** Breast cancer, Health care economics

## Abstract

**Background:**

There is limited evidence on the optimal frequency of mammogram surveillance. At 5-year follow-up, the Mammo-50 trial found that, in patients aged 50+ and 3 years post diagnosis, less frequent mammograms were non-inferior to annual mammograms for breast-cancer-specific-survival, recurrence-free interval and overall survival.

**Methods:**

A within-trial cost-effectiveness analysis compared annual versus less frequent mammogram surveillance over 5 years from healthcare and societal perspectives. Hospital Episodes Statistics captured hospital-based resource use. Health-related quality of life and other cost data were obtained via questionnaires at surveillance mammograms. A budget impact analysis estimated NHS savings.

**Results:**

Less frequent surveillance led to cost savings of −£543.88 (−£1116; £26) and a small reduction in quality-adjusted life years (QALYs) of −0.02 (−0.095; 0.06) per patient. The incremental net monetary benefit at a £20,000/QALY threshold was £187 (−£1574; £2027). Including societal costs increased savings to £1543 per person (−£2416; −£669), and cost-effectiveness. Projected NHS savings were £185.87 million over 6 years.

**Conclusion:**

Less frequent mammogram surveillance is cost-effective. Uncertainty remains due to variability in costs and quality of life estimates, and missing data in the less frequent arm due to study design. Given the trial’s non-inferiority findings, this strategy is recommended from healthcare and societal perspectives.

## Introduction

Current national guidance in the UK recommends that all patients treated for early and locally advanced breast cancer (including Ductal Carcinoma In Situ [DCIS]) are followed up with annual mammograms for 5 years [1]. However, there has been no robust evidence to support the optimum frequency and duration of mammographic surveillance [2]. An analysis of 53 randomised trials found that the proportion of loco-regional recurrences decreased significantly between 1990 and 2011 (from 30 to 15%), demonstrating the improved efficacy of surgical practice and systemic therapies in preventing recurrence in recent years [3]. Age is a significant predictor of poor mammography surveillance outcomes, with older age being a protective factor [4].

These findings suggest the possibility of safely reducing surveillance frequency in older patients treated for early and locally advanced breast cancer. This could benefit both patients and the health system. Less frequent mammograms may reduce overdiagnosis and associated anxiety associated with regular screening, though longer intervals could worsen anxiety form some. Reduced surveillance frequency could also ease the financial burden of breast cancer survivorship and relieve pressure on overstretched radiology services.

The Mammo-50 trial, a multicentre, randomised controlled, phase III non-inferiority trial, aimed to establish if patients treated surgically with curative intent for invasive and non-invasive breast cancer, aged 50 or over at diagnosis and with more than 3 years post-surgery, were not disadvantaged in terms of breast-cancer-specific-survival (BCSS), recurrence-free-interval (RFI) and overall-survival (OS) by having surveillance mammograms 2-yearly after conservation surgery and 3 yearly after mastectomy [5]. The main results of the trial demonstrated non-inferiority of less frequent mammograms compared to annual mammography across all three outcomes [5]. Health-related quality of life data was collected throughout the trial to assess the impact of surveillance frequency on outcomes beyond survival and explore whether less frequent follow-up significantly worsens patient’s quality of life.

Based on the data from the Mammo-50 trial and a linked analysis of Hospital Episodes Statistics (HES), here we report the results of a within-trial economic evaluation, estimating the cost-effectiveness of reduced mammographic surveillance. We also report the results of a budget impact analysis, estimating the annual National Health Service (NHS) savings if reduced frequency surveillance were adopted in national guidelines.

## Methods

Full details of the Mammo-50 trial (ISRCTN48534559) are published elsewhere [5]; however, key cohort details are summarised below for context.

Women aged 50 years or older at the time of initial diagnosis with either invasive breast cancer or DCIS were eligible, on the condition that they had received curative surgery and had no evidence of recurrence for 3 years following treatment [5]. Confirmation of no recurrence was required via mammography at year three [5], and participants were randomised between 36 and 44 months after initial therapeutic surgery [5]. There was no upper age limit specified in the eligibility criteria—inclusion of older participants was at the discretion of clinicians and patients, with the oldest participant aged 91 and the median age at randomisation 66 years [5]. Individuals were excluded if they had bilateral breast cancer (including bilateral DCIS), known BRCA or other high-risk genetic mutations, classical lobular carcinoma in situ, a prior diagnosis of breast malignancy, or a previous non-breast malignancy unless treated surgically with no recurrence for at least 10 years [5]. Individuals with a history of basal cell carcinoma of the skin or cervical intraepithelial neoplasia were not excluded [5].

Two economic evaluations were performed as part of the trial: (a) a within-trial analysis assessing the cost-effectiveness of annual mammography compared to less frequent surveillance over a 5-year period post-randomisation, using trial data linked with HES data [6], and (b) a budget impact analysis estimating NHS savings from reduced-frequency surveillance mammography.

More detail is provided in Supplementary Information file 1.

### Resource use data collection and costings

#### Mammograms

All surveillance mammograms were recorded via trial case report forms (CRFs). Participants were invited for mammograms based on their trial arm and, in the less frequent mammography arm, surgery type: 2-yearly (bilateral) for those after conservation surgery, and 3-yearly (contralateral) after a mastectomy. Participants could request a mammogram at any point during the trial, with reasons recorded.

Data collection timepoints were based on when scheduled mammograms occurred, so timepoint dates varied by participant depending on recruitment and mammogram timing, rather than following fixed calendar dates.

#### Hospital Episodes Statistics data

Due to the trial design, health-related quality of life and resource use data were collected only at mammogram visits, creating data availability imbalances between arms. To ensure we had robust hospital resource use data for all participants, we obtained HES data (Admitted Patient Care, Outpatient Care, and Critical Care) for each participant for a period of 5 years from their date of entry into the trial (2014–2023). These data captured all hospital activity, not limited to breast cancer-related care. Randomisation was expected to balance comorbidities and unrelated healthcare use across arms. As the last patient was randomised in September 2018, follow-up extends to 2023. Final timepoint cost data may be incomplete for later recruits due to data collection timing.

#### Other resource use

Participants completed a questionnaire at each surveillance mammogram, covering community-based health and social care use, prescribed and non-prescribed medications for breast cancer symptoms and side-effects, private or self-funded treatment, and other health-related expenses. Participants were also asked to provide details of time off work (for themselves or carers), incapacity benefits, travel and parking related to health appointments.

### Health-related quality of life

The questionnaire also included the EQ-5D-5L to capture health-related quality of life. UK utility values were derived by mapping the 5 L descriptive system data onto the 3 L value set—in line with the National Institute for Health Care Excellence (NICE) recommendations [7].

### Multiple imputation

Multiple imputation was to address the missing patient questionnaire data and the missing HES data at final timepoint, using the ‘mice’ [8] and ‘miceadds’ [9] packages in R (version 2024-06-14, The R Foundation for Statistical Computing, Vienna, Austria). Imputation was conducted separately by trial arm [10]. Given the longitudinal nature of the dataset, a hierarchical imputation model accounted for within- and between-patient variability. Twenty-five imputation datasets were generated, with up to 20 iterations each. Observed healthcare costs (e.g. hospital costs, prescribed medication costs) and EQ-5D index scores were included as predictors for missing healthcare costs and societal costs (e.g. other expenses, parking costs) to maintain consistency and comparability across analysis perspectives. Societal costs were not used to predict healthcare costs or EQ-5D index due to low correlations. Logarithmic transformations were applied to skewed cost variables. The trial stratification variables (e.g., age, type of disease, surgery type, hormone therapy at randomisation and ER status) and recurrence status at each timepoint were also included as predictors Diagnostic checks assessed validity.

ChatGPT was used to debug coding errors and warnings in R. After using this tool, PC reviewed and edited the imputation model as needed.

### Within-trial cost-effectiveness analysis

The base-case cost-effectiveness analysis adopted an NHS and Personal Social Services perspective, including: (i) hospital costs; (ii) costs of surveillance mammograms; (iii) community-based health and social care costs; and (iv) prescribed medications costs. Only data from timepoints four to eight were included to align with the HES 5-year follow-up.

Resource use was valued using 2023 unit costs from national sources (i.e. NHS Reference Costs [11], British National Formulary [12], Drugs and pharmaceutical electronic market information tool (eMIT) [13], and Personal Social Services Research Unit [PSSRU] unit costs [14]). Healthcare costs were inflated to 2023/24 using the NHS Cost Inflation Index [14–16], whereas non-healthcare expenses (e.g. private and self-funded treatment expenses, parking costs) were using the Consumer Price Inflation rates from the Office for National Statistics (ONS) [17]. Summary of the unit costs is provided in Supplementary Information file 2.

Due to uncertainty around the surveillance mammogram unit cost, the closest unit cost available (following discussion with NHS Digital) was that of a screening mammogram (NHS Cost Collection, £115.25 [11]). Consultations with participating trusts confirmed that surveillance mammograms are typically performed without consultation and resemble screening.

Patient health benefit was measured in quality-adjusted life years (QALYs), combining survival (life years) during follow-up and the associated health-related quality of life (utility) at each timepoint, using the area under the curve method. Survival was estimated as the number of years patients were alive during the follow-up. The primary outcome was the incremental net monetary benefit (INMB) at a willingness-to-pay (WTP) threshold of £20,000 per QALY [18].

#### Uncertainty analyses

To estimate uncertainty in the cost-effectiveness results, we conducted non-parametric bootstrapping with replacement using the ‘boot’ [19] package in R. For each of the 25 imputed datasets, 5000 bootstrapped samples were drawn with replacement. A Seemingly Unrelated Regression (SUR) model was fitted to each sample using the ‘systemfit’ [20] package, jointly estimating total costs and QALYs across all timepoints while controlling for trial stratification variables (age, type of disease, surgery type, hormone therapy at randomisation and ER status).

Bootstrapped estimates were used to plot the cost-effectiveness plane (CE) plane and cost-effectiveness acceptability curves.

#### Secondary analyses

Due to the absence of a standard unit cost for surveillance mammography, a sensitivity analysis was conducted using cost estimates from participating trusts. The wide range of reported costs informed the analysis.

A within-trial economic evaluation from a societal perspective was also undertaken, incorporating costs for non-prescribed medication, private and self-funded treatment, travel and parking, incapacity benefit, productivity loss, unpaid informal care and other healthcare-related expenses. These costs were collected via patient questionnaires administered at each surveillance mammogram. Unit costs were derived from national sources or estimated based on participant-reported expenses.

To make the most of available data up to the final timepoint, a 6-year follow-up analysis from a healthcare perspective was conducted, despite incomplete data for some participants recruited later in the trial.

There was a possibility that the outpatient care costs included some of the costs associated with the surveillance mammogram, therefore a sensitivity analysis was conducted excluding outpatient care costs to assess the impact on cost-effectiveness estimates. Subgroup analyses assessed variation in incremental costs and QALYs by stratification variables.

### Budget-impact analysis

A pragmatic budget impact analysis was conducted to estimate the NHS savings from implementing reduced-frequency surveillance mammography compared to annual surveillance mammography over a 6-year time horizon (2024–2029), using a simple cost calculator model.

The surveillance population was projected prospectively, accounting for prevalent cases, new diagnoses, survival probabilities and attrition. Savings began from year four post-diagnosis, as patients received annual surveillance for the first 3 years, in line with the main trial. Annual savings were estimated by multiplying annual per-patient cost savings by the number of patients in each surveillance phase per year. The expected number of mammograms saved annually was also estimated. No discounting was applied to costs, in line with the ISPOR Budget Impact Analysis Good Practice guidance [21]. One-way deterministic sensitivity analyses explored uncertainty in attrition rates, survival probabilities, and initial surveillance population, while scenario analyses varied mammogram unit costs.

## Results

Additional results are provided in Supplementary Information file 3.

### Base-case analysis

Return rates for patient questionnaires were initially high but declined over time (note missingness at timepoint nine is partially due to incomplete follow-up). In the less frequent arm, higher rates of missing data at timepoints four (94%), six (86%) and eight (99%) reflect the trial design, in which questionnaires were scheduled every 2 years for participants who had conservation surgery and every 3 years for those who had a mastectomy.

Table 1 summarises the total healthcare costs per patient by trial arm, post imputation. Outpatient care (£2631 vs. £2864) and mammogram costs (£206 vs. £524) were lower in the less frequent arm than the annual mammography arm. Community-based health and social care costs were slightly higher in the less frequent arm (£1002 vs. £960). Little difference was observed in prescribed medication costs and admitted patient care costs.

**Table 1:** Within-trial imputed healthcare and societal costs: summary statistics of the healthcare and societal costs per patient by mammogram follow-up frequency.

	Mammogram surveillance frequency						
	Arm 1: annual			Arm 2: less frequent (2-yearly and 3-yearly)			
Cost category	Mean	Median	SE	Mean	Median	SE	Mean difference
Healthcare perspective							
Community-based health and social care	£960	£560	£1431	£1002	£682	£1206	£41
Prescribed medications	£43	£0	£205	£37	£0	£182	−£6
Admitted patient care	£3579	£541	£8058	£3553	£496	£8486	−£26
Outpatient care	£2864	£2021	£2976	£2631	£1839	£2990	−£234
Mammograms cost	£524	£576	£121	£206	£231	£100	−£319
Total healthcare costs	£7971	£4434	£10,250	£7428	£3991	£10,590	−£543
Societal perspective							
Non-prescribed medications	£23	£0	£113	£20	£0	£92	−£3
Private and self-funded treatment	£372	£0	£2237	£232	£0	£1507	−£139
Other expenses	£214	£0	£1402	£172	£0	£1435	−£42
Travelling	£219	£109	£370	£188	£106	£269	−£31
Parking	£47	£15	£133	£44	£15	£131	−£3
Time off work	£634	£0	£2641	£499	£0	£2465	−£135
Incapacity benefits	£570	£0	£2819	£484	£0	£2651	−£86
Unpaid informal care	£2347	£0	£7608	£1848	£0	£6031	−£499
Time off work friends’ family	£150	£0	£1507	£87	£0	£840	−£63
Total societal costs	£12,546	£6867	£15,864	£11,003	£5745	£14,674	−£1543

Across both arms, the mean EQ-5D index score decreased slightly over time, but minimal differences were evident between arms (Table 2).

**Table 2:** Summary statistics of patient-reported EQ-5D index scores over time.

	Mammogram surveillance frequency						
	Arm 1: annual			Arm 2: less frequent (2-yearly and 3-yearly)			
Timepoint	Mean	Median	SE	Mean	Median	SE	Mean difference
4	0.784	0.7960	0.201	0.784	0.8260	0.2082	0.0004
5	0.779	0.7960	0.211	0.772	0.7950	0.2201	−0.0074
6	0.768	0.7960	0.229	0.765	0.7950	0.2320	−0.0036
7	0.760	0.7950	0.240	0.754	0.7950	0.2470	−0.0061
8	0.739	0.7680	0.263	0.740	0.7680	0.2569	0.0010

**Fig. 1:**
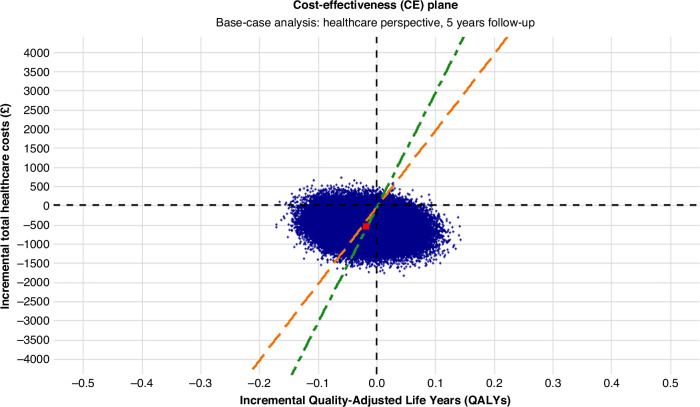
Base-case analysis cost-effectiveness plane with £20,000 (orange dashed line) and £30,000 (green dashed line) per QALY WTP thresholds and mean ICER (red square).

Figure 1 present the base-case bootstrapped cost-effectiveness results (the deterministic results can be found in Supplementary Information file 3). The mean incremental costs were −£543.88 (95% CI: −£1116 to £26) and the majority of the estimates on the cost-effectiveness plan (96.9%) lie below the x-axis indicating a very high probability that the less frequent arm is associated with health care cost savings. The mean incremental QALYs were −0.02 (95% CI: −0.095 to 0.06), suggesting that the less frequent arm yields slightly fewer QALYs on average. However, there was large amount of uncertainty associated with this estimate; 66% of the incremental QALY estimates were negative. At a WTP threshold of £20,000 per QALY gain, the INMB was £187 (95% CI: −£1574 to £2027), indicating that less frequent arm is marginally cost-effective albeit with considerable uncertainty. At a WTP threshold of £30,000 per QALY gain, the INMB decreased to £8 (95% CI: −£2498 to £2643), suggesting that the less frequent arm would be less cost-effective at this threshold.

At a WTP threshold of £20,000 per QALY, the probability of cost-effectiveness for less frequent surveillance is 58%. As the WTP threshold increases to £30,000 per QALY, the probability of cost-effectiveness for the less frequent arm decreases to 50%.

#### Secondary analyses

The mean differences in costs categorised as societal costs by trial arm can be found in Table 1. These cover the 5-year follow-up period and have been calculated post multiple imputation. The total societal costs were lower in the less frequent arm (£11,003) compared to the annual arm (£12,546), resulting in a mean difference of £1543 per patient. Across all costing categories, the less frequent arm was associated with lower costs. The largest cost savings were found in unpaid informal care (£499 lower in the less frequent arm), private and self-funded treatment (£139 lower), and time off work costs (£135 lower).

Cost-effectiveness results using a societal costing perspective are shown in Supplemental Information file 3. Compared to the base case, larger cost savings were observed (£1543 per person, 95% CI: −£2416 to −£669) due to inclusion of societal costs. The probability that the less frequent arm was cost saving increased to 86%. At a WTP threshold of £20,000 per QALY, the INMB rose to £1186 (95% CI: −£824 to £3270), which was higher than the base-case value of £187 due to the additional cost savings.

Extending the follow-up from 5 years to 6 years post-randomisation increased the cost savings (£734; 95% CI: −£1525 to £101), with little impact on incremental QALYs. The INMB increased to £339 (95% CI: −£1527 to £2142) at a WTP threshold of £20,000 per QALY, suggesting longer follow-up improves cost-effectiveness of less frequent mammogram surveillance.

Excluding outpatient care costs (i.e. assuming they reflect mammogram cost savings) reduced savings to £310 (95% CI: −£780 to £162), resulting in a negative INMB and loss of cost-effectiveness at the £20,000 per QALY threshold.

Cost-effectiveness varied across sub-groups, though small sample sizes limit interpretation (Supplementary Table 3.13). Notably, QALY losses were concentrated among patients receiving 3-yearly mammograms after mastectomy; no QALY difference and modest cost savings were observed for those undergoing local excision, who made up 80% of each arm and received 2-yearly mammograms. Subgroup analyses by age suggested a modest QALY gain and positive net monetary benefit for participants aged 53–55, while reduced surveillance appeared less likely to be cost-effective in those aged over 75. Among older patients, small QALY losses combined with minimal cost savings led to a negative INMB. These findings are subject to wide uncertainty given the small numbers in both the youngest (8%) and oldest subgroups (9%).

A sensitivity analysis was conducted to assess the impact of varying the unit cost of a surveillance mammogram on the INMB at a WTP of £20,000 per QALY. Higher mammogram unit costs (£272) led to greater cost savings for the less frequent arm (−£953 per patient), as expected, resulting in higher INMB values (£1,134,745). Conversely, as the unit cost decreased to values lower than the base case estimate (£33), the INMB became negative (−£595,137), as the cost savings no longer outweighed the slight decrement in QALYs.

#### Budget impact analysis

**Fig. 2:**
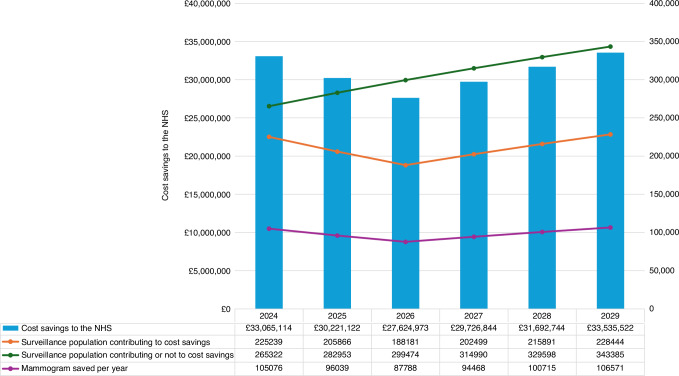
Base-case budget impact analysis results: total surveillance population, total surveillance population contributing to cost saving, annual cost savings to the NHS and mammograms saved per year over a 6-year horizon (2024–2029).

Figure 2 presents the total mammogram surveillance population and corresponding cost savings to the NHS England associated with implementing reduced-frequency mammogram surveillance compared to annual surveillance over a 6-year time horizon. The surveillance population grew annually due to the addition of new diagnoses and retention of existing patients, reaching a total of 1,835,722 patients, of whom a total of 1,266,119 (69%) contributed to cost savings due to reduced-surveillance. This growth in the surveillance population resulted in annual cost savings to the NHS increasing from £33,065,114 in 2024, due to the prevalent population, to £33,535,522 million in 2029. After 3 years, the implementation of reduced-surveillance mammography resulted in cumulative cost-savings of £90,911,208, whereas over the 6-year time horizon the cumulative cost savings to the NHS increased to £185,866,318. Over the 6-year time horizon (2024-2029), implementing reduced-frequency surveillance led to a cumulative reduction of 590,657 mammograms compared to standard annual surveillance.

## Discussion

The Mammo-50 trial demonstrated that less frequent mammogram surveillance in women aged 50 or over, treated surgically for breast cancer and with more than 3 years post-surgery, was non-inferior to current practice in terms of BCSS, RFI, and OS [5]. Here, we reported the within-trial cost-effectiveness analysis and budget impact analysis results for the Mammo-50 trial, exploring whether reduced mammographic surveillance was cost-effective from a healthcare and societal perspective.

Reducing the frequency of mammogram surveillance significantly reduced both healthcare and societal costs, with a high probability of being cost saving overall (healthcare perspective: 96.9%, societal perspective: 99.9%). Cost savings were primarily driven by lower mammogram costs and outpatient care costs, as well as societal costs such as reduced travel expenses and productivity losses. There was a small mean decrement in QALYs associated with the less frequent mammogram arm, but the difference was small and associated with uncertainty (66% probability of reducing QALYs). Reducing the frequency of mammogram surveillance had a 58% probability of being cost-effective from a healthcare perspective at a WTP threshold of £20,000 per QALY. At a WTP threshold of £30,000 per QALY, the probability of reduced mammogram frequency being cost-effective drops to 50%. These results highlight the uncertainty in both the incremental costs and QALYs. This is partially driven by the amount of missing data and reliance on multiple imputation, but we also observed large variability in the healthcare costs and EQ-5D index scores. Extending the follow-up period to 6 years led to increased cost savings and no notable impact on incremental QALYs, demonstrating that accounting for cost savings over a longer time horizon is likely to increase the cost-effectiveness of less frequent surveillance mammograms.

Sensitivity analyses showed, unsurprisingly, that the cost of a surveillance mammogram was a major determinant of cost-effectiveness. Not only was there wide heterogeneity in the cost estimates we obtained for a surveillance mammogram, but there was also some uncertainty about the extent to which any of the resource use associated with a surveillance mammogram was included in the outpatient HES costs. We are confident that they are not fully or consistently captured in the outpatient costs, as we compared the timing of the mammograms with the outpatient costs incurred for alignment (which was not evident). To explore the potential impact of this, we set outpatient cost differences to zero in a scenario analysis. Healthcare cost savings remained but were lower, and not large enough to offset the QALY reduction at a WTP threshold of £20,000 per QALY.

The findings of the subgroup analysis indicated the decrement in QALYs associated with the less frequent surveillance arm was primarily driven by those who had a mastectomy and therefore had 3-yearly surveillance. This may reflect higher missing EQ-5D data and reliance on multiple imputation, or slightly lower overall survival in this group, which - while non-significant based on the trial results (94.5% vs 94.7% at 5 years) [5]—would result in marginally fewer life years and QALYs. In contrast, reduced surveillance in participants who underwent wide local excision (representing 80% of the trial population) may represent a safe and efficient alternative with minimal impact on health outcomes, modest cost savings and potentially important implications for future surveillance policy.

Subgroup analyses by age showed a modest QALY gain and positive INMB in the youngest group (aged 53–55), suggesting that reduced surveillance may be more favourable in this group. Among participants aged over 75, however, reduced surveillance was associated with small QALY losses and minimal cost differences, resulting in a negative INMB. This may reflect higher overall healthcare use in older adults, which could reduce the incremental cost difference between arms and limit the extent to which cost savings offset modest QALY losses. Given the small sample sizes in both age subgroups, these findings should be interpreted with caution.

The budget impact analysis showed the potential financial benefits of implementing reduced-frequency surveillance mammography, which resulted in a cumulative £185.87 million cost savings for the NHS over 6 years. Savings were driven by the increasing proportion of patients transitioning from annual to reduced-frequency surveillance, ensuring that cost reductions aligned with real-world population dynamics. Sensitivity analysis highlighted that the unit cost of surveillance mammograms had a major influence on the magnitude of cost savings.

A key strength of the trial was the comprehensive collection of resource use data, covering a broad spectrum of healthcare and societal costs. A limitation, however, was that data collection—including health-related quality of life measures—was feasible only at the time of mammograms. As a result, fewer timepoints were available for the less frequent mammogram arm, necessitating substantial reliance on multiple imputation at certain timepoints. To address this, linking HES data from NHS England for trial participants over the follow-up period was crucial in providing robust information on hospital resource use, ensuring that we could explore the impact of mammogram frequency on hospital admissions, critical and outpatient care. This data was also used as a predictor in a multiple imputation approach that accounted for the hierarchical and longitudinal structure of the data and closely approximated the observed distributions. The budget impact analysis projections were based on trial-derived attrition and survival rates, which may not fully capture real-world variability. The cost saving estimates trial conditions and may not account for regional variations in healthcare delivery and the actual unit cost of surveillance mammograms across different NHS trusts.

The base-case results at WTP threshold of £20,000 per QALY supports the potential revision of current surveillance guidelines to allow less frequent mammographic follow-up. This approach could generate substantial cost savings for both the NHS and patients, while also freeing up mammography and radiology resources for other screening and diagnostic pathways. The main trial demonstrated clear non-inferiority in terms of key health outcomes. However, it can be challenging to convince policymakers to make changes when the ICER lies in the south-west quadrant of the CE plane, as seen here due to a slight QALY decrement in the less frequent arm. This is partly explained by marginally lower overall survival in the trial for the less frequent arm (albeit non-inferior and non-significant). The differences in EQ-5D were inconsistent over time, and heavily dependent on the multiple imputation model to overcome missing data, particularly in the less frequent arm. A sub-study of the Mammo-50 trial, where more comprehensive data was collected for a wide range of quality-of-life measures, observed no differences between the trial arms.

While this study provides valuable evidence to inform national policy, further research could explore variation in cost-effectiveness by social or geographic factors, or assess alternative surveillance strategies (e.g. remote mammography, physical examination plus ultrasound), particularly in under-served and isolated populations in the UK.

In conclusion, reduced mammogram surveillance is cost-effective compared to annual surveillance at NICE recommended WTP threshold of £20,000 per QALY, but there was considerable uncertainty associated with the results largely due to high variability both in costs and health-related quality of life. The budget impact analysis demonstrated that implementing this strategy on a national scale could lead to substantial cost savings for the NHS, reducing financial pressure on breast cancer surveillance programmes. These findings provide evidence to support the redesign of breast cancer follow-up, with significant cost savings to the NHS and reduced pressure on heavily constrained radiology services. Engaging with patients and healthcare providers will be crucial in developing and implementing a patient-centred surveillance strategy for breast cancer.

## Supplementary information

Supplementary Information files combined

## Data Availability

Data collected for the study, including individual participant data and a data dictionary defining each field in the set, will be made available to others after publication of the primary manuscript subject to approvals. Request for access should be made to Trial team at Warwick Clinical Trials Unit. After approval a signed data sharing agreement will be required prior to data release.
