# Supplementary material for: Annual versus less frequent mammographic surveillance in people with breast cancer aged 50 years and older in the UK (Mammo-50): cost-effectiveness and budget impact analysis
Source: Br J Cancer. 2025 Nov 6;134(2):252–8. doi: 10.1038/s41416-025-03248-2 (PMC12820096; doi:10.1038/s41416-025-03248-2)
Supplement: Supplementary file 1 — Supplementary Information files combined [file 41416_2025_3248_MOESM1_ESM.pdf]

Supplemental Information file 1: Methods

Costing resources from patient questionnaires

Community health and social care costs

Resource use information related to contacts or visits over the last three months with GP, practice nurse, district nurse, social workers, home help, complementary therapies provided by the NHS (e.g. acupuncture), NHS community-based clinics for post treatment side-effects (e.g. physiotherapy) was collected from patient questionnaires.

In addition, this costing category entailed complementary therapies provided by the NHS, NHS community-based clinics for post treatment side-effects over the last three months. Of these, the most common resource items were costed, including physiotherapy, counselling, traditional Chinese therapy (e.g. acupuncture, reflexology, podiatrist, and lymphoedema clinic.

The subcategory 'counselling' includes counselling sessions (in person or on the phone), psychotherapy, talking/listening services, well-being workshops, group therapies.

In case the number of contacts for a given resource was missing, the average number of contacts for the same resource item was manually imputed.

The number of contacts over the last three months was then multiplied with the unit cost for each costed resource item to estimate the total cost for each costed resource item.

Community health and social care costs for the three-month period were then multiplied by a factor of four to estimate annual costs, under the assumption that the reported period was representative of the full year.

Prescribed medication costs

Resource information related to prescribed medications taken to help women with continuing breast cancer symptoms and side-effects was collected from patient questionnaires. Patient reported the name of the prescribed medication, reasons as to why the medication was prescribed, date of start and end medication, and reason for stopping the medication.

Patients were instructed to list only medications/therapies prescribed since the last time the question was asked.

Data cleaning

The patient-reported names of prescribed medications were cleaned and coded into common categories. Medications which occurred more than once were costed.

The following prescribed medications were excluded from costing:

- Hospital-based prescribed medication (e.g. imaging, radiotherapy, treatment for breast cancer recurrence), as hospital costs were captured using the HES data;
- Medications classed as one-off cost (e.g. antibiotics, creams) with start date preceding trial recruitment;
- Medications with both start and end date preceding trial recruitment.

Sources for dosage recommendations and treatment duration

In line with the NICE guidelines, the recommended dosages for medications were based on the BNF [1]. In addition to this, the recommended dosage of brand drugs was retrieved from the electronic medicines compendium website [2]. Dosages reported as free text was used to sense check BNF recommended dosages, and clinical opinion was sought when multiple recommendations or ranges were available. Creams, ointments, eye drops, mouth products were costed as a one-off cost.

The duration of treatment of each prescribed medication was estimated using the dates of starting and ending treatment as reported in the questionnaires. Where end dates were missing, the expected duration was imputed based on the BNF, clinical guidelines and clinical opinion when necessary.

Sources for unit cost per medication and estimation of daily cost per medications

In line with the NICE guidelines, the cost for prescribed medications packages were based on the following sources (accessed in September 2024):

1. eMIT database – in case of multiple prices for the same active ingredient/medication, the price for the most commonly prescribed medication was selected[3]
2. British National Formulary unit costs[1]
3. NHS Drug Tariffs websites (see Supplementary Information file 2)
4. Boots websites (see Supplementary Information file 2)

The unit cost for each medication was estimated by dividing the cost per package by the number of tablets/capsules in each package. Based on the recommended dosage, the daily number of medications taken was estimated for each prescribed medication. The daily cost per medication was then calculated by multiplying the daily number of medications by the unit cost.

For medications classified as one-off costs (e.g., antibiotics, creams), the package cost was used directly to reflect their one-time usage, rather than calculating daily costs or treatment days.

Handling missing start and end dates of treatment

Missing start date for a prescribed medication was imputed as the date of patient completion when the prescribed medication was reported, as this reflects the earliest evidence of medication use. Missing end dates were imputed considering the expected duration of each prescribed medication based on clinical guidelines and assumptions (e.g. patients were assumed to take antidepressants until trial end date unless otherwise specified).

Medication propagation across timepoints

Using these imputed dates, we assigned each prescribed medication to specific timepoints in the follow-up period based on the (reported or imputed) end date and expected medication duration. If a medication spanned across multiple timepoints, it was propagated across all relevant timepoints between the (reported or imputed) start and end dates. This approach ensured that medications taken across timepoints were captured allowing for an accurate assessment of associated costs over time at each timepoint, while adhering to the questionnaire's wording.

We did not propagate prescribed medications across future timepoints in the timepoints where a patient was reported to be either dead, or withdrawn.

This approach allowed to evaluate the medication costs over time while addressing gaps in reporting and ensuring alignment with trial timelines and time points relevant for the analysis.

Standardised timeframes and treatment days estimation

To ensure consistent timeframes for cost calculations and fair comparisons across individuals, standardised one-year timeframes were established for each participant, regardless of differences in the actual follow-up schedules. Fixed timepoint-specific start and stop dates were assigned for each timepoint to ensure uniform follow-up intervals for all participants.

These dates were derived relative to their date of entry into the trial (DOE) and were structured as follows:

- For the first timepoint (three), stop date was set equal to the DOE, and the start date was set equal to NA, reflecting the initiation of the timeframe.
- For the subsequent timepoints, the start date corresponds to the stop date of the previous timepoint leading to contiguous intervals.
- The stop date of each timepoint was incremented by one year to ensure a uniform one-year duration for each interval.

This fixed timeframe dataset was merged with the cleaned and post-propagation prescribed medication dataset using TNO (individual ID) and relevant tp_number (timepoint) as identifiers.

For prescribed medications listed at timepoint three (baseline) and without a reported start date, timepoint-specific start date was defined depending on the type of medication:

- For long-term medication: it was assumed that the patient was already taking the medication at the time of trial entry. Timepoint-specific start date was imputed as the date of entry into the trial to ensure that their costs were included throughout the trial period or until the reported end date;
- For non-long-term medications: it was assumed that the medication use preceded the DOE and therefore fell outside the trial's cost window. The associated cost for timepoint three was estimated as zero.

A list of long-term prescribed and non-prescribed medication is provided in Supplementary Table 1.1.

Supplementary Table 1. 1 List of long-term prescribed medications

Accrete D3	Calcium supplement (undefined)	Glyceryl trinitrate	Propranolol
Adcal-D3	Candesartan	Hux D3	Pyridoxine
Adcal-D3 lemon	Carbocisteine	Hydroxychloroquine	Ramipril
Alendronic acid	Citalopram	Ibandronic acid	Risedronate
Allopurinol	Clenil	Indapamide	Ropinirole
Alogliptin	Clonidine hydrochloride	Injection (undefined)	Salamol inhaler
Amitriptyline	Clopidogrel	Isosorbide	Salbutamol
Amlodipine	Colecalciferol	Ivabradine	Sertraline
Anti depressant (undefined)	Denosumab	Lamotrigine	Simvastatin
Apixaban	Desunin	Levothryoxine	Spiriva inhalation powder
Atorvastatin	Diazepam	Lisinopril	Spironolactone
Bendroflumethiazide	Ditropan	Losartan	Statins (undefined)
Betahistine	Duloxetine	Metformin	Strontium ranelate
Bisoprolol	Escitalopram	Mirabegron	Sulfasalazine
Bisphosphonates (undefined)	Evacal D3	Mirtazapine	TheiCal-D3
Braltus Inhalation Powder	Evening Primrose oil	Multivitamins supplements	Tildiem
Bumetanide	Ezetrol	Natecal D3	Venlafaxine
Calceos	Felodipine	Nebivolol	Ventolin
Calcichew D3	Fluoxetine	Nortriptyline	Vitamin B supplement (undefined)
Calcichew forte	Fosavance	Paroxetine	Vitamin C+D supplement (undefined)
Calcit D3	Fostair inhaler	Perindropril	Vitamin D supplement (undefined)
Calcium + Vitamin D supplements (undefined)	Fultium D3	Pravastatin	Vitamin E supplement
Calcium carbonate	Furosemide	Premarin tablets	Warfarin

For each prescribed medication the number of treatment days within a timepoint was calculated directly as the difference between the start and stop dates assigned to that timepoint.

This approach ensured that the analysis used standardised timeframes for each timepoint which in turn allowed for consisted calculation of the treatment days and associated costs.

Total medication costs estimation

Using the calculated treatment days daily costs and propagated medications the total cost for each prescribed medication was estimated for each patient at each timepoint:

- Continuous (non-one-off) medications: the total cost was calculated by multiplying the daily cost per medication by the total treatment days (estimated as above) .
- One-off medications: these were costed based on the package cost rather than the duration of treatment reflecting their one-time usage.

For each patient the total prescribed medication costs were then aggregated at each timepoint summing up the costs for all prescribed medications recorded or propagated to that timepoint.

Non prescribed medication costs

Resource information related to non-prescribed medications taken to help women with continuing breast cancer symptoms and side-effects was collected from patient questionnaires. Patient reported the name of the non-prescribed medication, reasons as to why the medication was taken, date of start and end medication, and reason for stopping the medication.

Patients were instructed to list only medications/therapies taken since the last time the question was asked.

The patient-reported names of non-prescribed medications were cleaned and coded into common categories. Medications which occurred more than once were costed.

The following prescribed medications were excluded from costing:

- Hospital-based prescribed medication (e.g. iscador injection, treatment for breast cancer recurrence), as hospital costs were captured using the HES data;
- Medications classed as one-off cost (e.g. antibiotics, creams) with start date preceding trial recruitment;
- Medications with both start and end date preceding trial recruitment;
- Prescribed medications were excluded to avoid double-counting, as they may have already been listed in the prescribed medication section.

A similar approach was used for non-prescribed medications as described for prescribed medications, including (1) sources for dosage recommendations and treatment duration; (2) sources for unit cost per medication and estimation of daily cost per medications; (3) handling missing start and end dates for treatment; (4) medication propagation across timepoints; (5) medication propagation across timepoints; and (6) total medication cost estimation. Two key differences apply:

- Creams, ointments, eye drops, mouth products were costed as a one-off cost.
- For non-prescribed medications reported at baseline (timepoint 3) without a start date, treatment start was defined according to the type of medication. Long-term non-prescribed medications were assumed to be ongoing at trial entry and therefore included in cost calculations from the start of follow-up. In contrast, non-long-term medications were assumed to have been used prior to the date of entry and not costed at baseline. A full list of long-term non-prescribed medications is provided in Supplementary Table 1.2.

Supplementary Table 1. 2 List of long-term non-prescribed medications

Apple Cider Vinegar	Herbal remedies	Probiotics
Biotin supplement	Indapamide	Sea kelp
Calcium supplement	Magnesium supplement	Skin Nail Hair formula Boots
CBD	Menopace tablets	Vitamin C supplement (undefined)
CBD oil	Menopause supplements (undefined)	Vitamin D3 + K2
Claritin	Multivitamins supplements	Vitamin E supplement (undefined)
Cod liver oil	Nicorandil	Vitamin K2 supplement
Co-enzyme Q10 supplement	Oils (undefined)	Wellwoman 50+ tablets
Doxazosin	Omega 3 fish oil	Wheatgrass supplement
Ginger supplement	Osteocare tablets	Zinc supplement
Ginkgo biloba	Osteoguard tablets	
Green tea	Pomi-T	
Hair growth product (undefined)	Prebiotics + probiotics	

Private and self-funded treatment costs

The total amount (£) of health services social services or care paid by the patients over the last three months was collected through patient questionnaires. Costs covered by medical insurance were excluded from the analysis as these do not fall into the societal perspective.

To estimate annual private treatment costs reported costs were categorised into two groups:

- One-off private costs: services that are unlikely to recur within a year (e.g. major surgery specialised treatments home renovations);
- Periodic private costs: regular or recurring services (e.g. physiotherapy sessions routine consultations).

For periodic private costs reported costs for the previous three months were multiplied by a factor of four. This approach assumed that the periodic private costs incurred over the reported three month-period were representative of costs throughout the year. One-off private costs were treated as incurred once per year without any multiplication factor. Total annual private treatment costs were calculated by summing the annualized periodic private costs and the one-off private costs for each participant.

Each participants costs were inflated from the year of the questionnaires completion to the analysis year (2023) using the Consumer Price Inflation rates from the ONS [4].

Other expenses costs

The total amount (£) of other expenses due to patients health or treatment (e.g. home adaptions extra laundry cleaning services) over the last three months was collected from patient questionnaires.

Similarly to private treatment costs to estimate annual other expenses reported expenses were categorised into two groups:

- One-off expenses: services that are unlikely to recur within a year (e.g. home renovations purchase of walking sticks mobility scooters);
- Periodic other expenses: regular or recurring services (e.g. gardening cleaning and minor maintenance activities).

For periodic expenses for the previous three months were multiplied by a factor of four. This approach assumed that the periodic other expenses incurred over the reported three month-period were representative of costs throughout the year. One-off expenses were treated as incurred once per year without any multiplication factor. Total annual other expenses costs were calculated by summing the annualized periodic expenses and the one-off other expenses for each participant.

Each participants costs were inflated from the year of the questionnaires completion to the analysis year (2023) using the Consumer Price Inflation rates from the ONS [4].

Travel costs

Travel estimates to hospital or other health and social care appointments were collected from patient questionnaires which recorded the total miles travelled over the previous three months.

Travel costs were estimated multiplying the reported miles with the current NHS Mileage Allowance Payments (MAPS) rates car allowance which was set equal to 59p up to 3500 miles [5].

Each participant’s travelling costs were inflated from the year of the questionnaires completion to the analysis year (2023) using the Consumer Price Inflation rates from the ONS [4].

To estimate annual travel costs the three-months travel costs were multiplied by a factor of four. This approach assumed that the travel costs incurred over the reported three month-period were representative of costs throughout the year.

Parking costs

Healthcare-related parking estimates over the previous three months were collected from patient questionnaires.

Each participant's parking costs were inflated from the year of the questionnaires completion to the analysis year (2023) using the Consumer Price Inflation rates from the ONS [4].

To estimate annual parking costs the three-months parking costs were multiplied by a factor of four. This approach assumed that the parking costs incurred over the reported three month-period were representative of costs throughout the year.

Productivity loss cost

Data on time taken off work due to health conditions over the previous three months (hours) was collected from patient questionnaires. Employment status was also collected through patient questionnaires.

Productivity costs associated with time off work were estimated using the human capital approach based on median UK earnings for 2023 as reported by the ONS [6]. For full-time employees median weekly earnings were £682 assuming a 37.5-hour work week which corresponds to an hourly rate off £18.18. For part-time employees' median weekly earnings were £241 based on a 16.5-hour work week resulting in an hourly rate of £14.60.

Productivity costs associated with time off work were calculated based on the participants reported employment status. For those in full-time or part-time employment the respective hourly wage was applied to estimate the productivity costs by multiplying the number of hours missed over the previous three months by the hourly rate. Participants not in paid employment or retired were assigned a productivity cost of £0 as these groups were assumed not to incur any productivity losses. If employment status was missing participants were assumed to be in full-time employment to avoid underestimating costs.

To estimate annual productivity costs the three-months productivity costs associated with time taken off work were multiplied by a factor of four. This approach assumed that the productivity costs incurred over the reported three month-period were representative of costs throughout the year.

Unpaid informal care costs

Informal care provided by family or friends over the last three months was assessed through patient questionnaires. Participants were first asked if they have received help or support from family and

friends and then to report the total time spent (hours) over the last three months. In addition, participants indicated if their helpers took time off work to provide this support and if so the total hours of work missed over the same period.

The value of informal care was estimated using the human capital approach based on median UK earnings for 2023 [6]. We applied the same hourly rate used to estimate the productivity lost costs. As the employment status of the helpers was unknown, we conservatively assumed full-time employment to estimate the worst-case scenario.

Informal care costs were calculated as the sum of two components:

- Unpaid informal care costs: total time spent helping multiplied by the hourly rate.
- Productivity costs: total hours of work missed multiplied by the same hourly rate.

To estimate annual informal care costs the three-month costs were multiplied by a factor of four. This approach assumed that the costs incurred during the reported period were representative of annual care costs.

Incapacity benefit costs

Data on patients' employment status including whether they were on sick leave and receiving incapacity benefits were collected from patient questionnaires. The questionnaires however did no provide specific details regarding the amount of incapacity benefit received the state pension age or the type of pension.

To estimate the annualised incapacity benefit costs a standardised short-term incapacity benefit rate of £130.20 per week (2023/2024)[7] was applied and multiplied by the number of weeks in one year on average (52.14).

Hospital Episode Statistics costs

Hospital-based healthcare activity was obtained from Hospital Episode Statistics (HES) data provided by NHS England for the duration of participants involvement in the trial. Datasets were requested for admitted patient care (APC) critical care and outpatient care (OP) for 2014 – 2022.

Hospital costs were divided as follows:

- Admitted Patient Care (APC) costs: which included admitted patient care and critical care;
- Outpatient costs (OP) costs: which covered outpatient cost.

APC Finished Consultant Episode (FCE) data were grouped into Continuous InPatient Spells (CIPS, also known as Super-Spells) according to NHS Digital criteria (Health & Social Care Information

Centre. CIPS definitions accommodate transfers within a day across providers as one patient interaction rather than as two separate interactions. Duplicate FCEs nested completely within a CIPS (indicating a patient was in two places at the same time) were deleted to avoid double counting. The HRG4+ 2022/2023 NHS National Costs Grouper[8] was used to attribute CIPS level Healthcare Resource Groups (HRGs) to each CIPS as well as length of stay. Unbundled activity, high-cost activity like MRI scans which are paid separately from the core HRG, are also identified. Length of stay was adjusted by any relevant Critical Care days to avoid double counting of any excess bed days.

As National Costs are no longer reported at CIPS level, unit costs were derived from the NHS 2022/2023 National Tariff Payment System [9]. In addition to providing resource use at CIPS level, the tariff also accommodates excess bed day costs. Tariffs are considered a more accurate representation of resource use as they do not require arbitrary rules of costing multi-FCE CIPs and allow for higher costs for longer stays.

Tariffs in 2022/2023 prices were mapped to core HRGs, excess bed days and Unbundled HRGs where relevant. Costs were matched according to elective or non-elective admission and at HRG level. Where HRG specific tariffs were not available costs where matched at sub-chapter and then chapter averages. Non-published unbundled HRG prices were imputed using national sources or local prices.

Critical Care data were attached to the relevant CIPs and costed by the number of organs supported and length of stay.

Outpatient appointments were also assigned HRGs via the national costs grouper and matched to tariff prices in a similar process to admitted patient care data. Appointments that were either cancelled or unattended were assigned a zero cost.

Monthly costs for each participant enrolled in the trial were then reported separately for each hospital cost category. To integrate these costs into the analysis, costs were matched to individual participants using their unique patient ID. Costs were then assigned to specific trial timepoints based on the reported month of observations. Timepoints were derived using predefined monthly intervals to ensure consistency with the trials longitudinal structure. Specifically, each month of observation was assigned to a corresponding trial timepoint as follows:

- Baseline (timepoint 3): costs reported for the first month of the trial (month = 1);
- Timepoint 4: costs reported between month 2 and 12;
- Timepoint 5: costs reported between month 13 and 24;
- Timepoint 6: costs reported between month 25 and 36;
- Timepoint 7: costs reported between month 37 and 48;

- Timepoint 8: costs reported between month 49 and 60.

Addressing double-counting and redundancies across costing categories

To ensure the accuracy of cost estimates and avoid double-counting the following approaches were implemented to handle redundancies among the costing categories:

- Duplication between prescribed medications and community-based health and social care costs: costs associated with prescribed medications were retained as the recall period for this costing category was longer and therefore was considered more reliable than the recall period for community-based health and social care costs.
- Duplication between hospital activities reported as prescribed medications and hospital costs: any hospital-related prescribed medication (e.g. lypo-modelling ER+ maintenance treatment iscador injection) were removed from prescribed medication costs . These costs were assumed to be captured under hospital costs.
- Redundancy between community-based health and social care costs reported as out-of-pocket expenses (in free text) and private/self-funded treatment costs: private treatment expenses were retained and entries in the first category were treated as typo errors or repetitions made by the patient.
- Duplication between other expenses explicitly related to travel and parking for hospital appointments and separately reported travel and parking costs: costs related to travel and parking were retained and entries in the first category were treated as repetitions made by the patient.

Multiple imputation methods

To achieve complete cost and health-related quality of life data for the trial participants, we employed multiple imputation to address missing patient questionnaire data. This was conducted using the ‘mice’ and ‘miceadds’ packages in R version 2024-06-14 (The R Foundation for Statistical Computing, Vienna, Austria) which employ a chained equations approach. Imputation was carried out by trial arm to capture arm-specific variability and potential differences across arms [10].

Given the longitudinal nature of the dataset, where repeated measurements were available for each individual across six timepoints, we conducted a hierarchical multiple imputation. This approach accounted for the within-patient variability in costs and EQ5D index scores over time, as well as variability across individuals with similar characteristics.

Categorization of missing data

Each variable’s missingness was flagged and managed accordingly using a set of logical rules:

- For participants indicating no use of a resource (e.g. no prescribed medications or no private treatment), costs were set equal to £0.
- Missing data for participants who explicitly reported using a resource but failed to specify a cost were classified as missing at random.
- Missing data for participants who returned the questionnaire but failed to provide estimates for any or all EQ5D index dimension explicitly were classified as missing at random.

For each costing variable, an overview of the logical rules to define missing values is provided in Supplementary Table 1.3.

Supplementary Table 1. 3 Overview of the logical rules to define missing values sorted for costing variables

Costing category	Rules for setting cost value equal to 0	Rules for setting cost value equal to missing at random
Community health and social care (chsc) costs	Respondent stated no CHSC or listed CHSC resources which either<br>• did not occur often in the dataset.<br>• were removed because they occurred outside of the analysis period.<br>• were removed due to redundancies with other costing categories	Respondent stated CHSC use but did not provide costs, or the CHSC section was incomplete .
Prescribed medication costs	Respondent stated no prescribed medications, or listed medications which either<br>• occurred only once in the dataset.<br>• were removed due to redundancies with other costing categories.<br>• were removed because they occurred outside of the analysis period.	Respondent listed prescribed medications, but costs were missing, or entries were unintelligible.
Non-prescribed medication costs	Respondent stated no non-prescribed medications, or listed medications which either<br>• occurred only once in the dataset.<br>• were removed due to redundancies with other costing categories.<br>• were removed because they occurred outside of the analysis period.	Respondent listed non-prescribed medications, but costs were missing, or entries were unintelligible.
Private and self-funded treatment costs	Respondent stated no private treatment, or costs were fully funded by insurance, or costs were removed due to redundancies with other costing categories.	Respondent stated private treatment but did not provide costs, or the private treatment section was incomplete.

Other expenses	Respondent stated no other expenses or costs were removed due to redundancies with other costing categories.	Respondent stated other expenses but did not provide costs, or the other expenses section was incomplete.
Travel costs	Respondent stated no travel costs.	Travel cost section was left blank or incomplete.
Parking costs	Respondent stated no parking costs.	Parking cost section was left blank or incomplete.
Productivity loss costs (time off work)	Respondent stated no time off work.	Time off work section was left blank or incomplete.
Informal care (help from family and friends) costs	Respondent stated no unpaid informal care.	Unpaid informal care section was left blank or incomplete.
Family and friends time off work costs	Respondent stated no time off work for family or friends.	Time off for family and friends section was left blank or incomplete.
Incapacity benefit costs	Respondent stated no incapacity benefits.	Incapacity benefit section was left blank or incomplete.

Missingness for cost categories and EQ5D index was categorised into the following types:

- By design (type one): missing data was due to the study design (e.g. participants not required to complete the questionnaire at certain timepoints due to the follow-up frequency);
- Deceased participants (type two): missing data due to participant death;
- At random (type three): missing data occurring without a clear reason, such as skipped responses;
- Withdrawn participants (type four): missing data due to participants withdrawing from the study.

Missing values were initially left as NA and subsequently imputed using a multiple imputation model. For deceased participants, EQ-5D-5L scores were imputed as 0 from the date of death until the end of the trial, assuming a linear transition from the last recorded non-zero score to zero. Similarly, costs

were set to 0 at timepoints where participants were recorded as deceased. This approach minimized bias in the estimates for living participants with similar characteristics. For withdrawn participants, imputed values were retained, consistent with the principles of an intention-to-treat analysis.

Imputed variables

Costs were imputed for each at the disaggregated level, rather than unit of resource level. Missing quality of life was imputed at the EQ5D index score level, rather than at the domain level. The imputation targeted EQ5D index and key cost variables at each timepoint, including:

- Healthcare costs: community-based health and social care costs, prescribed medications costs and hospital costs (admitted patient care and outpatient care costs);
- Societal costs: non-prescribed medication costs, private and self-funded treatment expenses, other expenses, travelling and parking costs, incapacity benefit costs, productivity loss costs, unpaid informal care costs.

Data collected at baseline [i.e. age at randomisation, type of surgery, type of disease (DCIS only, invasive disease), HER2 status, ER status] were used as predictors of every cost component and EQ5D index in the imputation model. Additionally, whether a patient experienced a breast cancer (BC) or non-BC recurrence at a given timepoint was included as a predictor.

Predictor selection and model structure

Prescribed and non- prescribed medication costs were included in the analysis, but they were not used as predictors for other costing variables and EQ5D index. This decision was made due to the significant amount of missing data, poor reporting of prescription durations and dosages, and the heavily skewed distribution of the data, which included many zeroes. These factors made the reliability of prescribed and non-prescribed medication costs questionable as predictors.

Observed healthcare costs values were included as predictors of other healthcare costs and EQ5D index in the imputation models. In addition, observed healthcare costs and EQ5D index were also included in the imputation for societal costs to ensure consistency and comparability across analysis perspectives, and to preserve the relationships between societal costs and healthcare costs. Observed societal costs values, however, were not used to predict healthcare costs and EQ5D index due to low and negligible correlations observed between these two categories. The decision to use healthcare costs and EQ5D index as predictors for societal costs (but not vice versa) was supported by the stronger completeness and stability of healthcare cost and quality of life data.

Observed societal costs, excluding non-prescribed medication costs (due to poor reporting and unreliability), were used to predict other societal costs alongside risk stratification variables. Observed

non-prescribed medication costs were excluded as predictors for societal costs for similar reasons as prescribed medication costs.

Imputation methodology

The two-level predictive mean matching (2l.pmm) method was applied to account for both individual-level variability and time variability across repeated measures. This method ensured that the imputed values fell within the range of the observed data.

To address skewness in the distribution of cost variables and facilitate imputation, we applied logarithmic transformations to healthcare and societal cost variables in the datasets. After completing the imputation, the logarithmic transformations were reversed to return the variables to their original scale.

The multiple imputation was run creating 25 imputed datasets and setting the maximum number of 20 iterations to determine the imputed values. This was shown to provide accurate and stable estimates for the imputed values compared to the observed values, while keeping the computational burden manageable. A series of diagnostic checks were performed to ensure the validity and robustness of the imputed values.

Cost-effectiveness analysis approach

Resource use data collected from patient questionnaires were estimated at each timepoint (ranging from three [baseline] to nine [last timepoint]). For the base-case analysis (i.e. healthcare perspective), however, only data from timepoints four to eight were included to align with the follow-up of the HES data and ensure consistency in the estimation of costs during the defined five-year follow-up period. Costs estimated at each timepoint reflect the resource use accrued during the preceding year. For example, timepoint four reflects costs for years three-four, timepoint five reflects costs for years four-five.

Patient health benefit was expressed in terms of quality-adjusted life years (QALYs), combining estimates of patient survival (life years) during follow-up and the associated health-related quality of life (utility) at each timepoint. The area under the curve (AUC) method was used to calculate QALYs. The EQ-5D-5L index value at baseline (timepoint three) was used to estimate the AUC contribution for the interval between timepoints three and four. However, only the AUC contributions from timepoint four onwards, reflecting data collected one year after the trial start, were included in the calculation of total QALYs per patient. The AUC contribution calculated for the interval including timepoint three was excluded from the total QALYs estimation, as the follow-up period explicitly begins at timepoint four.

Following imputation, cost-effectiveness analysis was performed separately on each imputed dataset and combined using Rubin's rules. To quantify uncertainty in the cost-effectiveness results, we applied non-parametric bootstrapping with replacement – for each of the 25 imputed datasets, 5,000 bootstrapped samples were generated, and a Seemingly Unrelated Regression (SUR) model was fitted to each sample.

Budget impact analysis methods

In the absence of significant differences in mortality or health outcomes between trial arms based on the trial results [11], a budget impact analysis (BIA) was conducted to estimate the net financial cost savings to the NHS of implementing reduced-frequency surveillance mammography compared to annual surveillance mammography.

A cost calculator model was developed in Microsoft Excel to estimate the financial impact of both trial arms over a six-year time horizon (2024–2029). Annual results were presented to capture changes in the surveillance population and associated costs over time. Consistent with ISPOR Budget Impact Analysis Good Practice guidance[12], costs were not discounted .

Population assumptions

The BIA population included all patients eligible for mammogram surveillance in the trial (i.e. women older than 50 years old with three years post diagnosis), covering:

- DCIS (stage 0).
- Invasive breast cancer (stages I–III).

The total surveillance population was dynamically projected annually from 2024 to 2029, accounting for:

- Prevalence: the existing population of breast cancer survivors under surveillance, derived from latest available England prevalence estimates (2024) [13, 14].
- New diagnoses: annual incident cases from 2023-2021 for each stage DCIS, stage I, stage II, and stage III) were used to project future diagnoses using an annual growth rate approach [15, 16].
- Survival probabilities: stage-specific yearly overall survival probabilities, derived from publicly available NHS England Cancer Survival Statistics [17, 18].
- Attrition rates: annual reductions due to stage-specific attrition rates derived from the trial.

To ensure accurate cost saving estimation, the population was stratified by year in reduced surveillance, reflecting the transition from annual mammogram surveillance (years 1-3) to reduced-frequency surveillance (>year 4).

Population estimation

The BIA account for both the prevalent and incident breast cancer populations eligible for mammographic surveillance.

Prevalent population

The prevalent population eligible for reduced-frequency surveillance was estimated using the latest available breast cancer prevalence for 2024[14], which reported 710,000 women living with breast cancer in the England.

We applied an age-specific proportion from the National Disease Registration Service (NDRS) ten-year incidence data (2021)[13] to restrict the population to those aged 50 years or older in line with trial eligibility criteria, resulting in 619,145 individuals aged 50 or older living with breast cancer in 2024.

To estimate the proportion of patients eligible for reduced surveillance, we used the NDRS 10-year prevalence estimates (2021)[13]. Based on these data, we estimated that 77% of individuals with a breast cancer diagnosis within the past 10 years had more than three years since diagnosis. The final prevalent population eligible for reduced-frequency surveillance was estimated at 478,138 individuals.

To differentiate prevalent population by stage, we applied the stage-specific proportions taken from five-year incidence NDRS (2021)[13] as it was the only prevalence data available sorted by stage. DCIS cases were excluded at this stage due to paucity of data.

Incident population

The incident population comprised new breast cancer diagnoses expected from 2024 onward. Annual estimates of new diagnoses were stratified by stage using stage-specific incident data from Early Cancer Diagnosis Hub [15].

Since the latest available stage-specific incidence data was from 2021, projections for 2024-2029 were estimated using the annual average growth rate (AAGR) calculated from incidence trends between 2013 and 2021.

For each stage (DCIS, I-III), the AAGR was estimated as:

$$AAGR = \left(\frac{incidence_{2021}}{incidence_{2013}}\right)^{\frac{1}{2021-2013}} - 1$$

Stage-specific annual estimates of new diagnoses for 2024 were then projected as below:

$$estimated\ incidence_t = incidence_{2021} \times (1 + AAGR)^{t-2021}$$

Once the total number of new diagnoses was estimated for each year, an age proportion was applied to ensure that the projected cases included only individuals aged 50 years or older, in line with the Mammo-50 trial eligibility criteria. Historical data from Cancer Registration Statistics (2019-2022)[16] was used to estimate the age distribution of new cases. Based on these data, 86% of new DCIS cases and 82% of invasive breast cancer cases (stage I-III) were diagnosed in women aged 50 years or older.

Newly diagnosed patients received annual surveillance for the first three years post-diagnosis and entered the reduced-frequency surveillance population from year four onwards, at which point cost savings were realised.

Annual per-patient cost savings estimation

The base-case bootstrapped per-patient cost saving estimate (£734 per patient over six years) from the within-trial economic evaluation represented cumulative cost savings accrued over time. Applying this estimate would therefore overestimate cost savings in the BIA, as it does not account for the timing of cost accrual.

To ensure an appropriate distribution of cost savings, the six-year cumulative estimate was converted into an annual per-patient cost saving by timing the total amount evenly across six years.

The final yearly per-patient cost savings were then assigned based on each patient's surveillance phase. Patients in the reduced-surveillance phase (i.e. three years post diagnosis for incident population and all patients in the prevalent population) were assigned the annual per-patient cost savings each year. This approach ensured that cost reductions were applied only to those eligible for reduced surveillance in line with trial protocol .

Data sources

The parameters used in the BIA are summarized in Supplementary Table 1.4.

Supplementary Table 1. 4 Parameters used in the budget impact analysis: base-case estimate, upper and lower bound, data source and notes

Parameter	Base-case estimate	Lower bound	Upper bound	Source	Notes
Prevalent cases in 2024 eligible for mammogram surveillance	478,138	/	/	[13, 14]	Prevalence dated 2024 (710,000) multiplied by age-specific proportion from 10-year prevalence NDRS (87%), and by proportion of patients post 3 year after diagnosis from the 10-year prevalence NDRS (77%). Stage-specific proportions are estimated from 5-year prevalence NDRS (stage I: 47%, stage II: 44%, stage III: 9%) by removing stage IV and unknown stage and recalculating the proportions. Prevalence estimates exclude DCIS.
Annual number of new DCIS diagnoses (stage 0)	2024: 6,656<br>2025: 6,642<br>2026: 6,628<br>2027: 6,614<br>2028: 6,600<br>2029: 6,586	- 25%	+25%	[15, 16]	Projections (2024-2029) were estimated using the annual average growth rate (-0.21%) calculated from incidence trends between 2013 and 2021. Age-specific proportion was estimated using the midpoint value for carcinoma in situ of breast (86%) from Cancer Registration Statistics England 2019-2022. Counts, age-specific and directly age-standardised rates of cancer incidence by ICD-10 codes (3-digit), geographic region, age-group, IMD quintile and sex . Information on whether DCIS diagnoses were unilateral or bilateral was not available in the data sources used. The projected annual number of new DCIS diagnoses therefore includes both unilateral and bilateral cases.
Annual number of new diagnoses of early breast cancer (stage I)	2024: 15,270<br>2025: 15,432<br>2026: 15,595<br>2027: 15,760	- 25%	+25%	[15, 16]	Average of rolling average of observed breast cancer (early stage I). Projections (2024-2029) were estimated using the annual average growth rate (1.06%) calculated from incidence trends between 2013 and 2021. Age-specific proportion was estimated using the midpoint value for malignant neoplasm of breast (82%) from Cancer Registration Statistics England

	2028: 15,927<br>2029: 16,096				2019-2022. Counts, age-specific and directly age-standardised rates of cancer incidence by ICD-10 codes (3-digit), geographic region, age-group, IMD quintile and sex .
Annual number of new diagnoses of early breast cancer (stage II)	2024: 14,896<br>2025: 15,156<br>2026: 15,421<br>2027: 15,691<br>2028: 15,965<br>2029: 16,244	- 25%	+25%	[15, 16]	Projections (2024-2029) were estimated using the annual average growth rate (1.75%) calculated from incidence trends between 2013 and 2021. Age-specific proportion was estimated using the midpoint value for malignant neoplasm of breast (82%) from Cancer Registration Statistics England 2019-2022. Counts, age-specific and directly age-standardised rates of cancer incidence by ICD-10 codes (3-digit), geographic region, age-group, IMD quintile and sex .
Annual number of new diagnoses of early breast cancer (stage III)	2024: 3,261<br>2025: 3,282<br>2026: 3,303<br>2027: 3,324<br>2028: 3,345<br>2029: 3,366<br>2023: 1,399	- 25%	+25%	[15, 16]	Projections (2024-2029) were estimated using the annual average growth rate (0.64%) calculated from incidence trends between 2013 and 2021. Age-specific proportion was estimated using the midpoint value for malignant neoplasm of breast (82%) from Cancer Registration Statistics England 2019-2022. Counts, age-specific and directly age-standardised rates of cancer incidence by ICD-10 codes (3-digit), geographic region, age-group, IMD quintile and sex .

Attrition rate DCIS (stage 0)	9%	7%	11.25%	[11]	Estimated as the total patients withdrawn with DCIS by the total number of patients with DCIS in arm 2 less frequent arm .<br><br>Upper and lower bound estimated as +/- 25% from the base-case estimate .
Attrition rate invasive disease (stage I-III)	6%	5%	7.5%	[11]	Estimated as the total patients withdrawn with DCIS by the total number of patients with DCIS in arm 2 less frequent arm .<br><br>Upper and lower bound estimated as +/- 25% from the base-case estimate .
Yearly overall survival estimate DCIS (stage 0)	1	0.99	1	[17, 18]	Overall survival - assumed same rate as stage I as DCIS specific survival rates are not available .
Yearly overall survival estimate stage I	0.9817	0.9807	0.9826	[18]	Base-case 5-yearly overall survival equal to 0.912 .
Yearly overall survival estimate stage II	0.9698	0.9687	0.9707	[18]	Base-case 5-yearly overall survival equal to 0.858.
Yearly overall survival estimate stage III	0.9453	0.9423	0.9483	[18]	Base-case 5-yearly overall survival equal to 0.755.

Yearly per patient cost saving	£146.8	404.27	760.45	MAMMO-50 within-trial cumulative per-patient cost savings over 6 years (healthcare perspective)	Cumulative per-patient cost saving estimate is divided by 6.

Attrition rates were derived from withdrawal rates observed in the trial. These rates reflect real-world reasons for discontinuation, including patient preferences (e.g., opting out due to reduced frequency), non-compliance with follow-up protocols, and practical barriers to continued surveillance.

In the absence of England-specific data on attrition rates for mammogram surveillance, the trial data provided a robust and relevant proxy for estimating annual reductions in the surveillance population.

Overall survival was used instead of net survival to provide a more realistic estimate of population retention, accounting for deaths from all causes rather than being limited to breast cancer-specific mortality. This approach ensures the projections align with real-world demographic characteristics and competing risks faced by patients under mammogram surveillance.

Base-case analysis

The total budget impact was calculated by multiplying the incremental cost per patient by the projected surveillance population contributing to cost savings for each year. Annual costs were summed over the six-year horizon to estimate cumulative financial savings. The analysis accounted for dynamic changes in the surveillance population, driven by attrition, survival, and new diagnoses.

To estimate the reduction in mammograms associated with the implementation of reduced-frequency surveillance, we first determined the expected number of mammograms in both arms. Under annual surveillance, each patient in the cost-saving phase would receive one mammogram per year.

For the reduced-frequency arm, mammogram frequency varied based on trial-defined surveillance schedules, which depended on the type of surgery received. Patients who underwent breast-conserving surgery received a mammogram every two years (80.3%), while mastectomy patients received a mammogram every three years (19.7%), based on Mammo-50 trial estimates for the total population [11]. This corresponded to an average of 0.46651 mammograms per patient per year, indicating a 53.349% reduction in annual mammograms per patient compared to standard annual surveillance.

The total number of mammograms performed each year was estimated by multiplying the surveillance population contributing to cost savings in each arm by the respective per-patient mammogram rates. The number of mammograms saved per year was calculated as the difference between the total mammograms under annual surveillance and those under the reduced-frequency strategy.

Annual estimates of mammogram reductions were projected for the time horizon 2024–2029, accounting for changes in the surveillance population contributing to cost savings over time.

Sensitivity and scenario analysis

To explore parameter uncertainty, one-way deterministic sensitivity analyses were conducted by varying the following key parameters:

- Attrition rates: adjusted by ±10% for DCIS and invasive stages;
- Overall survival probabilities: adjusted by ±25%;
- Initial surveillance population: adjusted by ±25%.

Scenario analyses were performed to evaluate the financial implications of varying the unit cost of surveillance mammogram, based on consultations of several trusts involved in the Mammo-50 trial, and consequently the incremental costs per patient. Supplementary Table 1.5 presents a range of mammogram unit cost explored within the scenario analysis. Correspondingly, the yearly per-patient cost saving varies depending on the mammogram unit cost.

Supplementary Table 1. 5 Cumulative incremental cost saving per patient and yearly incremental cost saving per patient for each mammogram unit cost explored within the scenario analysis

Mammogram unit cost	Cumulative incremental cost per patient	Yearly incremental cost per patient
£272.0	-£953.0	£158.8
£208.6	-£864.3	£144.1
£200.0	-£852.2	£142.0
£115.3	-£734.0	£146.8
£80.0	£684.3	£114.1
£75.0	£677.3	£112.9
£51.5	£644.5	£107.4
£40.4	£628.9	£104.8
£33.0	£618.6	£103.1
£14.4	£592.5	£98.8

References

1. British National Formulary. BNF (British National Formulary). https://bnf.nice.org.uk/. Accessed 01/10/2024.
2. compendium em. https://www.medicines.org.uk/emc. Accessed 01/08/2024.
3. Department of Health and Social Care. Drugs and pharmaceutical electronic market information tool (eMIT) - 1 January 2023 to December 2023. 2023. Personal Social Services Research Unit (University of Kent) & Centre for Health Economics (University of York). Accessed 30/09/2024.
4. Office for National Statistics. Consumer price inflation time series (MM23). 2024. https://www.ons.gov.uk/economy/inflationandpriceindices/timeseries/d7bt/mm23. Accessed 01/10/2024.
5. NHS Staff Council. NHS Terms and Conditions of Service Handbook. 2024. https://www.nhsemployers.org/publications/tchandbook. Accessed 16/10/2024.
6. Office for National Statistics. Average actual weekly hours of work for part-time workers (seasonally adjusted) 2024. https://www.ons.gov.uk/employmentandlabourmarket/peopleinwork/earningsandworkinghours/timeseries/ybvb. Accessed 16/10/2024.
7. Department of Work & Pensions. Benefit and pension rates 2024 to 2025. 2024. https://www.gov.uk/government/publications/benefit-and-pension-rates-2024-to-2025/benefit-and-pension-rates-2024-to-2025. Accessed 16/10/2024.
8. NHS England. HRG4+ 2022_23 National Costs Grouper. https://digital.nhs.uk/services/national-casemix-office/downloads-groupers-and-tools/hrg4-2022-23-national-costs-grouper. Accessed 01/08/2024.
9. NHS England. National tariff payment system documents, annexes and supporting documents. https://www.england.nhs.uk/publication/national-tariff-payment-system-documents-annexes-and-supporting-documents/. Accessed 01/08/2024.
10. Faria R, Gomes M, Epstein D, White IR. A Guide to Handling Missing Data in Cost-Effectiveness Analysis Conducted Within Randomised Controlled Trials. PharmacoEconomics. 2014; doi: 10.1007/s40273-014-0193-3.
11. Dunn JA, Donnelly P, Elbeltagi N, Marshall A, Hopkins A, Thompson AM, et al. Annual versus less frequent mammographic surveillance in people with breast cancer aged 50 years and older in the UK (Mammo-50): a multicentre, randomised, phase 3, non-inferiority trial. The Lancet. 2025; doi: 10.1016/S0140-6736(24)02715-6.
12. Sullivan SD, Mauskopf JA, Augustovski F, Jaime Caro J, Lee KM, Minchin M, et al. Budget Impact Analysis—Principles of Good Practice: Report of the ISPOR 2012 Budget Impact Analysis Good Practice II Task Force. Value in Health. 2014; doi: https://doi.org/10.1016/j.jval.2013.08.2291.
13. National Disease Registration Service. England Cancer Prevalence Statistics, 2021. 2021. https://nhsd-ndrs.shinyapps.io/prevalence/. Accessed 15/03/2025.
14. Support MC. Cancer prevalence. https://www.macmillan.org.uk/dfsmedia/1a6f23537f7f4519bb0cf14c45b2a629/19581-10061/2025-macmillan-prevalence-estimates. Accessed 17/03/2025.
15. Cancer Intelligence Team at Cancer Research UK. Early Cancer Diagnosis Data Hub. https://crukcancerintelligence.shinyapps.io/EarlyDiagnosis/. Accessed 14/03/2025.
16. NHS Digital. Cancer registrations statistics, England https://digital.nhs.uk/data-and-information/publications/statistical/cancer-registration-statistics. Accessed 13/03/2025.
17. Cancer Research UK. Survival for breast cancer. 2023. https://www.cancerresearchuk.org/about-cancer/breast-cancer/survival. Accessed 08/03/2025.
18. NHS Digital. Cancer Survival in England, cancers diagnosed 2016 to 2020, followed up to 2021. 2023. https://digital.nhs.uk/data-and-information/publications/statistical/cancer-survival-in-england/cancers-diagnosed-2016-to-2020-followed-up-to-2021. Accessed 08/03/2025.

Supplementary Information file 2 – Unit costs

Supplementary table 2. 1 Unit cost of community health and social care services

Cost item	Estimate	Inflated estimate*	Source	Notes
GP surgery visit	£56	£57.62	PSSRU 2023 (pg 64)	Per surgery consultation lasting 10 minutes, including direct care staff costs, including qualification costs
GP home visit	£169.20	£174.11	PSSRU 2023 (pg 64)	Including direct costs, qualifications. Assume 30-minute consultation. 30 min X £5.64 per minute of patient contact = £169.200
Telephone consultation with GP	£40.04	£41.21	PSSRU 2023 (pg 64)	Per patient contact assuming a 7.1 minute call including direct staff costs - £5.64 per minute of patient contact multiplied by 7.1 [page 127]
Practice nurse visit	£13.69	£14.09	PSSRU 2023 (pg 62)	Per hour of face-to-face contact including qualifications costs, assuming 15.5 minutes (see duration of contact in PSSRU 2015). Need to estimate the per minute cost = 53/60 = 0.8833*15.5 min
Practice nurse phone consultation	£6.18	£6.36	PSSRU 2023 (pg 62)	per hour of including qualifications, assuming a 7-minute telephone phone call. Per minute cost = £53/60 = 0.88333*7
District nurse home visit	£33.50	£41.77	PSSRU 2015 (pg 169)	per hour of patient related work, including qualifications, assuming a 30-minute consultation. Per minute cost = £67/60 = £1.11666668.
Social worker visit	£26.50	£27.27	PSSRU 2023 (pg 75)	Cost including qualification. Including qualifications. Assume a 30-min appointment and divide hourly cost to estimate cost per minute (£53 per hour/60 min = 0.88333 per minute)*30 min
NHS/Social Services home help or other home care	£27.00	£27.78	PSSRU 2023 (pg 78)	Assume 1 hour
Physiotherapist face-to-face visit	£20.50	£21.09	PSSRU 2023 (pg 56)	Assume a 30-minute. Assume community-based physiotherapist in band 5. Estimate per minute cost = 41£/60 = 0.68333

Physiotherapist phone contact	£4.78	£4.92	PSSRU 2023 (pg 56)	Assume a 7-minute phone call. Assume community-based physiotherapist in band 5. Estimate per minute cost = 41£/60 = 0.68333
Counsellor face-to-face visit	£26.50	£27.27	PSSRU 2023 (pg 56)	Assume a 30-minute appointment. Assume counsellor in band 6 [band 5 is entry level). Estimate per minute cost = 53£/60 = 0.88333
Counsellor phone contact visit	£6.18	£6.36	PSSRU 2023 (pg 56)	Assume a 7-minute appointment. Assume counsellor in band 6 [band 5 is entry level). Estimate per minute cost = 53£/60 = 0.88333
Podiatrist	£20.50	£21.09	PSSRU 2023 (pg 56)	Assume a 30-minute appointment [same assumption applied to physiotherapist]. Assume community-based physiotherapist in band 5. Estimate per minute cost = 41£/60 = 0.8833
Acupuncturist	£53	£56.06	PSSRU 2023 (pg 56)	Only NHS Reference costs specific to acupuncture at the hospital. Assume band 6 community-based and professional staff, 1h visit (https://www.nhs.uk/conditions/acupuncture/)
Lymphoedema clinic	£39.75	£40.90	PSSRU 2023 (pg 56)	Band 6 community-based scientific and professional staff. Duration follow-up visit: 45 minutes based on Thomas et al 2023 Int Wound J. Lymphoedema specialist (band 6). Need to calculate per minute cost = 53/60 = £0.8833
Mammogram	£112	£115.25	NHS National Cost Collection 2022/2023	NHS breast screening programme (NSP002). Inflated to 2023/2024

Supplementary table 2. 2 Recommended dosage, duration and unit costs of prescribed medications

Name prescribed medication	Category	Duration (days)	Assumptions	Dosage	Dosage frequency	Number of dosages per frequency	Cost package (£)	Unit cost (£)	Sources and details
Accrete D3	Vitamin	Until end of trial	If start date given, but no stop date, assume end of trial	1.5 gram + 400 unit	Daily	2	2.95	0.05	60 tablets https://bnf.nice.org.uk/drugs/colecalciferol-with-calcium-carbonate/medicinal-forms/ Dosage from https://www.medicines.org.uk/emc/files/pil.2766.pdf
Adcal-D3	Vitamin	Until end of trial	If start date given, but no stop date, assume end of trial	4 caplets	Daily	4	3.54	0.03	112 tablets - Adcal-D3 750 mg/200 unit caplets https://bnf.nice.org.uk/drugs/colecalciferol-with-calcium-carbonate/medicinal-forms/ Dosage from https://www.medicines.org.uk/emc/files/pil.4723.pdf

Adcal-D3 lemon	Vitamin	Until end of trial	If start date given, but no stop date, assume end of trial	1500 mg + 400 units	Daily	2	4.38	0.08	56 tablets https://bnf.nice.org.uk/drugs/colecalciferol-with-calcium-carbonate/medicinal-forms/ Dosage from https://www.medicines.org.uk/emc/product/401/smpc#gref
Alendronic acid	Osteoporosis	Until end of trial	If start date given, but no stop date, assume end of trial	10mg	Daily	0.142857	0.18	0.05	Alendronic acid 70mg tablets / Pack size 4 (eMIT database)
Allopurinol	Gout	Until end of trial	If start date given, but no stop date, assume end of trial	300mg	Daily	3	0.31	0.01	Allopurinol 100mg tablets / Pack size 28
Alogliptin	Diabetes	Until end of trial	If start date given, but no stop date, assume end of trial	25mg	Daily	1	26.6	0.95	28 tablets (25mg) https://bnf.nice.org.uk/drugs/alogliptin/medicinal-forms/
Amitriptyline	Antidepressant	Until end of trial	If start date given, but no stop date,	10mg	Daily	1	0.23	0.01	Amitriptyline 10mg tablets / Packs

			assume end of trial						ize 28 (eMIT database)
Amlodipine	Cardiac treatment	Until end of trial	If start date given, but no stop date, assume end of trial	5mg	Daily	1	0.19	0.01	Amlodipine 5mg tablets / Packsize 28 (eMIT database)
Amoxicillin	Antibiotic	5	One off cost	500mg every 8 hours	Daily	2	0.66	0.66	Amoxicillin 1g powder for solution for injection vials / Packsize 1 (eMIT database)
Anovate Cream	Cream	One off	One off cost	1	One-off	1	2.49	2.49	Anusol HC ointment https://bnf.nice.org.uk/drugs/benzyl-benzoate-with-bismuth-oxide-bismuth-subgallate-hydrocortisone-acetate-peru-balsam-and-zinc-oxide/medicinal-forms/
Anti depressant (undefined)	Antidepressant	Until end of trial	If start date given, but no stop date,	20mg	Daily	1	0.31	0.01	Citalopram 20mg tablets / Packs

			assume end of trial						ize 28 (eMIT database)
Antibiotics (undefined)	Antibiotic	5	One off cost	250mg 4 times	Daily	2	1.74	0.062143	Flucloxacillin 500mg capsules / Packsize 28
Antihistamines (undefined)	Anti-allergy	One off	One off cost	10mg	One-off	1	0.25	0.008333	Cetirizine 10mg tablets / Packsize 30 Same as cetirizine
Anti-inflammatory medication (undefined)	Anti-inflammatory	1	One off cost	500mg	Daily	2	0.59	0.02	Naproxen 250mg tablets / Packsize 28 (eMIT database)
Apixaban	Cardiac treatment	Until end of trial	If start date given, but no stop date, assume end of trial	5mg twice	Daily	4	2.91	0.29	10 tablets A A H Pharmaceuticals https://bnf.nice.org.uk/drugs/apixaban/medicinal-forms/ Dosage https://bnf.nice.org.uk/drugs/apixaban/#indications-and-dose (it is usually twice 2.5mg daily)

AproDerm Cream	Cream	One off	One off cost	1	One-off	1	3.99	3.99	500gr Aproderm cream https://bnf.nice.org.uk/drugs/emollient-creams-and-ointments-paraffin-containing/medicinal-forms/
Aqueous cream (undefined)	Cream	One off	One off cost	1	One-off	1	3.85	3.85	https://www.boots.com/boots-pharmaceuticals-aqueous-cream-500g-10277133?cm_mmc=bmm-buk-google-ppc-_-PLAs_HeroCompare-_--_-PMax:+UK_Smart_Shopping_Healthcare_Other&gad_source=1&gclid=CjwKCAiA_5WvBhBAEiwAZtCU79vSfsx9uXptSvl824MK1J1ZZ2jwRMZfB9PyCa26ckwKf1p_E-

									DzcxoCFEIQAvD_BwE&gclsrc=aw.ds
Aspirin	Pain relief	2	One off cost	75mg	Daily	1	0.29	0.01	Aspirin 75mg dispersible tablets / Packsize 28 (eMIT database)
Atorvastatin	Cardiac treatment	Until end of trial	If start date given, but no stop date, assume end of trial	10mg	Daily	0.78	0.03	0.02	Atorvastatin 40mg tablets / Packsize 28 (eMIT database)
Aveeno cream	Cream	One off	One off cost	1	One-off	1	6.8	6.8	300ml https://bnf.nice.org.uk/drugs/emollient-creams-and-ointments-colloidal-oatmeal-containing/medicinal-forms/
Bendroflumethiazide	Cardiac treatment	Until end of trial	If start date given, but no stop date, assume end of trial	2.5mg	Daily	0.21	0.01	0.01	Bendroflumethiazide 5mg tablets / Packsize 28 (eMIT database)
Betahistine	Sickness	Until end of trial	If start date given, but no stop date,	24mg	Daily	3	1.47	0.02	Betahistine 8mg tablets / Packs

			assume end of trial						ize 84 (eMIT database)
Betnovate Cream	Cream	One off	One off cost	1	One-off	1	4.05	4.05	100gr GlaxoSmithKline https://bnf.nice.org.uk/drugs/betamethasone/medicinal-forms/
Bisoprolol	Cardiac treatment	Until end of trial	If start date given, but no stop date, assume end of trial	10mg	Daily	4	0.24	0.01	Bisoprolol 2.5mg tablets / Packsize 28 (eMIT database)
Bisphosphonates (undefined)	Osteoporosis	Until end of trial	If start date given, but no stop date, assume end of trial	10mg	Daily	0.29	0.4	0.1	Risedronate sodium 35mg tablets / Packsize 4 (eMIT database)
Bowen therapy	Chsc treatment	3	One off cost	0	Daily	1	56.06	56.06	Physiotherapist face to face (band 6) PSSRU
Braltus Inhalation Powder	Asthma inhaler	Until end of trial	If start date given, but no stop date, assume end of trial	1 capsule	Daily	1	25.8	0.86	30 capsules (10mg) https://bnf.nice.org.uk/drugs/tiotropium/medicinal-forms/

Brufen Retard	Pain relief	2	One off cost	1.6g	Daily	2	7.74	0.14	56 tablets (800mg)
Budenoside	Steroids	7	One off cost	200mg twice	Daily	1.6	14.45	0.72	Budesonide 1mg/2ml nebuliser liquid unit dose vials / Packsize 20 (eMIT database)
Bumetanide	Cardiac treatment	Until end of trial	If start date given, but no stop date, assume end of trial	1mg	Daily	1	1.59	0.06	Bumetanide 1mg tablets / Packsize 28 (eMIT database)
Calceos	Vitamin	Until end of trial	If start date given, but no stop date, assume end of trial	1250mg +400 unit	Daily	2	4.05	0.07	60 tablets https://bnf.nice.org.uk/drugs/colecalciferol-with-calcium-carbonate/medicinal-forms/ Dosage from https://www.medicines.org.uk/emc/product/3747/smpc#gref
Calcichew D3	Vitamin	Until end of trial	If start date given, but no stop date, assume end of trial	1000mg	Daily	1	7.29	0.24	30 tablet https://bnf.nice.org.uk/drugs/colecalciferol-with-calcium-

									carbonate/medicinal-forms/
Calcichew forte	Vitamin	Until end of trial	If start date given, but no stop date, assume end of trial	100mg	Daily	1	14.21	0.24	60 tablets https://bnf.nice.org.uk/drugs/calcium-carbonate/medicinal-forms/
Calcit D3	Vitamin	Until end of trial	If start date given, but no stop date, assume end of trial	500mg	Daily	1	4.06	0.14	30 sachets https://bnf.nice.org.uk/drugs/colecalciferol-with-calcium-carbonate/medicinal-forms/ Dosage 1: one or two sachets per day (taken the smallest amount) https://www.medicines.org.uk/emc/product/14780/smpc#gref
Calcium + Vitamin D supplements (undefined)	Vitamin	Until end of trial	If start date given, but no stop date, assume end of trial	4 caplets	Daily	4	3.54	0.03	112 tablets - Adcal-D3 750 mg/200 unit caplets https://bnf.nice.org.uk/drugs/colecalciferol-with-calcium-

									carbonate/medicinal-forms/ Dosage from https://www.medicines.org.uk/emc/files/pil.4723.pdf
Calcium carbonate	Vitamin	Until end of trial	If start date given, but no stop date, assume end of trial	150mg twice	Daily	0.171429	12.31	0.41	Calcium lactate gluconate 2.327g / Calcium carbonate 1.75g effervescent tablets sugar free (Sandocal 1000) / Packsize 30 (eMIT database)
Calcium supplement (undefined)	Vitamin	Until end of trial	If start date given, but no stop date, assume end of trial	150mg twice	Daily	0.171429	12.31	0.41	Calcium lactate gluconate 2.327g / Calcium carbonate 1.75g effervescent tablets sugar free (Sandocal 1000) / Packsize 30 (eMIT database)

Candesartan	Cardiac treatment	Until end of trial	If start date given, but no stop date, assume end of trial	8mg	Daily	2	0.34	0.01	Candesartan 4mg tablets / Packsize 28 (eMIT database)
Canesten Cream	Cream	One off	One off cost	5g for 1 dose	One-off	1	4.41	4.41	50gr Canesten 1% cream https://bnf.nice.org.uk/drugs/clotrimazole/medicinal-forms/
Carbimazole	Hyperthyroidism treatment	365	One off cost	50mg	Daily	10	2.32	0.02	Carbimazole 5mg tablets / Packsize 100 (eMIT database)
Carbocisteine	Asthma inhaler	Until end of trial	If start date given, but no stop date, assume end of trial	1.5g	Daily	4	2	0.01	Carbocisteine 375mg capsules / Packsize 120 (eMIT database)
Carbomer Eye Gel	Eye lubricant	One off	One off cost	3 times	One-off	1	1.29	1.29	Carbomer 0.2% eye gel 10 gram https://bnf.nice.org.uk/drugs/carbomers/medicinal-forms/
Celecoxib	Pain relief	14	One off cost	200mg	Daily	2	1.64	0.027333	Celecoxib 100mg capsules / Pac

									ksize 60 (eMIT database)
Cetirizine	Anti-allergy	One off	One off cost	10mg	One-off	1	0.25	0.01	Cetirizine 10mg tablets / Packsize 30 (eMIT database)
Cetraben Cream	Cream	One off	One off cost	1	One-off	1	4.17	4.17	150 gram bottle BNF https://bnf.nice.org.uk/drugs/emollient-creams-and-ointments-paraffin-containing/medicinal-forms/
Chiropractic	Chsc treatment	3	One off cost	0	Daily	1	56.06	56.06	Physiotherapist face to face (band 6) PSSRU
Ciprofloxacin	Antibiotic	7	One off cost	250mg twice	Daily	1	0.59	0.06	Ciprofloxacin 500mg tablets / Packsize 10 (eMIT database)
Citalopram	Antidepressant	Until end of trial	If start date given, but no stop date, assume end of trial	20mg	Daily	1	0.34	0.01	Citalopram 20mg tablets / Packsize 28 (eMIT database)

Clenil	Asthma inhaler	Until end of trial	If start date given, but no stop date, assume end of trial	200 mg twice daily	Daily	2	16.17	0.081	Clenil Modulite 200micrograms/dose inhaler
Clobetasol	Steroids	One off	One off cost	1	One-off	1	2.56	2.56	ClobaDerm 30gr https://bnf.nice.org.uk/drugs/clobetasol-propionate/medicinal-forms/
Clonidine hydrochloride	Cardiac treatment	Until end of trial	If start date given, but no stop date, assume end of trial	50mg twice	Daily	2	2.95	0.026339	Clonidine 25microgram tablets / Packsize 112 (eMIT database)
Clopidogrel	Cardiac treatment	Until end of trial	If start date given, but no stop date, assume end of trial	75mg	Daily	1	0.79	0.03	Clopidogrel 75mg tablets / Packsize 28 (eMIT database)
Co-amoxiclav	Antibiotic	5	One off cost	250/125 mg every 8 hours	Daily	2	1.88	0.09	Co-amoxiclav 500mg/125mg tablets / Packsize 21 (eMIT database)
Co-codamol	Pain relief	3	One off cost	1 capsules every 4 hours	Daily	6	0.98	0.03	Co-codamol 30mg/500mg tablets / Packs

									ize 30 (eMIT database)
Codeine phosphate	Pain relief	3	One off cost	30mg	Daily	1	1.46	0.05	Codeine 30mg tablets pre-labelled TTA / Packsize 28 (eMIT database)
Co-dydramol	Pain relief	3	One off cost	10mg/500mg every 6 hours	Daily	4	3.53	0.04	Co-dydramol 10mg/500mg tablets / Packsize 100 (eMIT database)
Colecalciferol	Vitamin	Until end of trial	If start date given, but no stop date, assume end of trial	400 units	Daily	0.5	1.26	0.04	Colecalciferol 800unit capsules / Packsize 30 [Maintenance dose for treatment D deficiency] (eMIT database)
Complementary therapy	Chsc treatment	3	One off cost	0	One-off	1	21.09	21.09	Physiotherapist face to face (band 6) PSSRU
Cosmocol	Constipation	14	One off cost	4 sachets	Daily	4	1.97	0.1	Macrogol compound oral powder sachets NPF sugar free (orange) (e.g.

									Laxido, Macroherm,Movicol,cosmocol,laxagol,macilax) / Packsize 20 (eMIT database)
Counselling	Chsc treatment	4	One off cost	0	Daily	1	27.27	27.27	Counsellor face to face visit PSSRU
Cream (undefined)	Cream	One off	One off cost	1	One-off	1	6.83	6.83	500gr Epaderm ointment
Cyclizine	Sickness	2	One off cost	50mg twice	Daily	2	6.68	0.67	Cyclizine 50mg/1ml solution for injection ampoules / Packsize 10 (eMIT database)
Denosumab	Osteoporosis	Until end of trial	If start date given, but no stop date, assume end of trial	60mg every 6 months	Half-yearly	1	183	183	Prolia solution for injection pre-filled syringes https://bnf.nice.org.uk/drugs/denosumab/medicinal-forms/ Dosage from https://bnf.nice.org.uk/drugs/denosumab/#ind

									ications-and-dose
Dermol Lotion	Cream	One off	One off cost	1	One-off	1	6.04	6.04	Dermol 500ml lotion https://bnf.nice.org.uk/drugs/emollient-creams-and-ointments-antimicrobial-containing/medicinal-forms/
Desunin	Vitamin	Until end of trial	If start date given, but no stop date, assume end of trial	400 units daily	Daily	2	3.6	0.12	800unit tablets https://bnf.nice.org.uk/drugs/colecalciferol/medicinal-forms/
Diazepam	Antidepressant	Until end of trial	If start date given, but no stop date, assume end of trial	2mg 3 times	Daily	3	0.36	0.01	Diazepam 2mg tablets / Packsize 28 (eMIT database)
Diethylamine salicylate	Pain relief	One off	One off cost	Once or twice	One-off	1	1.61	1.61	Surgical spirit A A H Pharmaceuticals https://bnf.nice.org.uk/drugs/diethyl-phthalate-with-methyl-

									salicylate/medicinal-forms/
Difflam	Pain relief	One off	One off cost	4 sprays every 1.5 hours	One-off	1	2.84	2.84	Benzydamine 0.15% oromucosal spray sugar free 30 ml / Packsize 1 (eMIT database)
Dihydrocodeine	Pain relief	One off	One off cost	30mg every 4 hours	Daily	6	1.39	0.05	28 tablets (30mg) A A H Pharmaceuticals https://bnf.nice.org.uk/drugs/dihydrocodeine-tartrate/medicinal-forms/
Diprobase Cream	Cream	One off	One off cost	1	One-off	1	7.01	7.01	500ml Drug Tariff https://www.drugtariff.nhsbsa.nhs.uk/#/00852760-DD/DD00852741/Part%20IXA-Appliances
Ditropan	Treatment for underactive bladder	Until end of trial	If start date given, but no stop date,	5mg twice	Daily	4	1.96	0.023333	Oxybutynin 2.5mg tablets / Packs

			assume end of trial						ize 84 (eMIT database)
Docusate	Constipation	5	One off cost	120 mg for 1 dose	Daily	2.5	18.32	0.061067	300ml Docusate 50mg/5ml oral solution sugar free https://bnf.nice.org.uk/drugs/docusate-sodium/medicinal-forms/
Domperidone	Sickness	7	One off cost	10mg 3 times	Daily	3	0.22	0.01	Domperidone 10mg tablets / Packsize 30 (eMIT database)
Duloxetine	Antidepressant	Until end of trial	If start date given, but no stop date, assume end of trial	60mg	Daily	1	1.24	0.04	Duloxetine 60mg gastro-resistant capsules / Packsize 28 (eMIT database)
E45 Cream	Cream	One off	One off cost	Twice	One-off	1	2.88	2.88	200ml https://bnf.nice.org.uk/drugs/emollient-creams-and-ointments-paraffin-containing/medicinal-forms/

Edoxaban	Cardiac treatment	90	One off cost	60mg	Daily	1	49	1.75	Lixiana 60mg tablets https://bnf.nice.org.uk/drugs/edoxaban/medicinal-forms/ Dosage https://bnf.nice.org.uk/drugs/edoxaban/ (assuming up to 61kg)
Epaderm Cream	Cream	One off	One off cost	1	One-off	1	6.83	6.83	500gr Epaderm ointment
Escitalopram	Antidepressant	Until end of trial	If start date given, but no stop date, assume end of trial	10mg	Daily	1	0.5	0.02	Escitalopram 10mg tablets / Packsize 28 (eMIT database)
Esomeprazole	Gastrointestinal treatment	30	One off cost	20mg	Daily	0.5	3.06	3.06	Esomeprazole 40mg powder for solution for injection vials / Packsize 1 (eMIT database)
Estradiol	Menopause treatment	24	One off cost	10 micrograms	Daily	1	10.6	0.441667	Estradiol 10microgram pessaries/vaginal tablets (e.g. vagifem) / Packsize 24

									(eMIT database)
Estriol Cream	Cream	One off	One off cost	1	One-off	1	4.52	4.52	Estriol 0.1% cream 15 gram (1mg/g) / Packsize 1 (eMIT database)
Evacal D3	Vitamin	Until end of trial	If start date given, but no stop date, assume end of trial	1500mg	Daily	2	2.75	0.05	56 tablets https://bnf.nice.org.uk/drugs/colecalciferol-with-calcium-carbonate/medicinal-forms/ Dosage from https://www.drugs.com/uk/evacal-d3-1500-mg-400-iu-chewable-tablets-leaflet.html
Evening Primrose oil	Vitamin	Until end of trial	If start date given, but no stop date, assume end of trial	60mg	Daily	1	5.31	0.09	https://www.boots.com/boots-evening-primrose-oil-1000-mg-60-capsules-10149542
Ezetrol	Cardiac treatment	Until end of trial	If start date given, but no	10mg	Daily	1	1.58	0.06	Ezetimibe 10mg

			stop date, assume end of trial						tablets / Packsize 28 (eMIT database)
Felodipine	Cardiac treatment	Until end of trial	If start date given, but no stop date, assume end of trial	5mg once	Daily	1	0.89	0.03	Felodipine 5mg modified-release tablets / Packsize 28 (eMIT database)
Fenbid Ibuprofen Gel	Pain relief	One off	One off cost	1	One-off	1	4.09	4.09	Unit price https://ayp.healthcare/fenbid-ibuprofen-5-gel-100g
Ferrous sulphate	Vitamin	365	One off cost	200mg once	Daily	1	0.57	0.02	Ferrous sulfate 200mg tablets / Packsize 28 (eMIT database)
Flucloxacillin	Antibiotic	5	One off cost	250mg 4 times	Daily	2	1.74	0.06	Flucloxacillin 500mg capsules / Packsize 28 (eMIT database)
Fluoxetine	Antidepressant	Until end of trial	If start date given, but no stop date, assume end of trial	20mg	Daily	1	0.42	0.01	Fluoxetine 20mg capsules / Packsize 30 (eMIT database)
Folic acid	Vitamin	16	One off cost	5mg	Daily	1	0.25	0.01	Folic acid 5mg tablets / Packs

									ize 28 (eMIT database)
Fosavance	Osteoporosis	Until end of trial	If start date given, but no stop date, assume end of trial	1 tablet	Weekly	1	22.8	5.7	Fosavance tablets (BNF https://bnf.nice.org.uk/drugs/alendronic-acid-with-colecalciferol/medicinal-forms/)
Fostair inhaler	Asthma inhaler	Until end of trial	If start date given, but no stop date, assume end of trial	4 inhalation	Daily	4	29.32	0.24	Fostair 200micrograms/dose 6 micrograms/dose inhaler 120 doses https://bnf.nice.org.uk/drugs/beclometasone-with-formoterol/medicinal-forms/
Fucibet Cream	Cream	One off	One off cost	1	One-off	1	6.38	6.38	Fucibet cream 30gr https://www.dermatologyhandbook.co.uk/companies/leo-pharma/fucibet-cream/

Fultium D3	Vitamin	Until end of trial	If start date given, but no stop date, assume end of trial	1 capsule	Daily	1	3.6	0.12	30 tablets (800 unit) https://bnf.nice.org.uk/drugs/colecalciferol/medicinal-forms/ Dosage https://www.medicines.org.uk/emc/files/pil.2813.pdf
Furosemide	Cardiac treatment	Until end of trial	If start date given, but no stop date, assume end of trial	20mg	Daily	0.5	0.27	0.01	Furosemide 40mg tablets / Packsize 28 (eMIT database)
Fybogel	Constipation	7	One off cost	1 sachet twice	Daily	2	4.77	0.16	30 sachets https://bnf.nice.org.uk/drugs/ispaghula-husk/medicinal-forms/
Gabapentin	Pain relief	30	One off cost	300mg three times	Daily	3	1.8	0.02	Gabapentin 300mg capsules / Packsize 100
Garment/support - bra	Garment	4	One off cost	1	Daily	1	16.22	16.22	NHS Drug Tariff page 612 Juzo Expert - Knitted-in, seamless bra cup. Assume

									change every 4 months (3 times each year)
Garment/support - gloves	Garment	4	One off cost	1	Daily	1	70.56	70.56	NHS Drug Tariff pg 579 Cicatrex Open Finger Glove price per piece. Assume change every 4 months (3 times each year)
Garment/support - gloves & socks	Garment	4	One off cost	1	Daily	1	99.3	99.3	NHS Drug Tariff Cicatrex Open Finger Glove + Sigvaris Active Work Wear Sock. Assume change every 4 months (3 times each year). Assume change every 4 months (3 times each year)
Garment/support - pressure bandage	Garment	4	One off cost	1	Daily	1	10.92	10.92	NHS Drug Tariff March 2024 page 612-613. Assume

									change every 4 months (3 times each year)
Garment/support - sleeve	Garment	4	One off cost	1	Daily	1	15.47	15.47	NHS Drug Tariff pag 596 Jobst Bella Lite Armsleeve with Knitted Band (Class 1). Assume change every 4 months (3 times each year)
Garment/support - sleeve & bra	Garment	4	One off cost	1	Daily	1	31.69	31.69	Sleeve + Bra NHS Drug Tariff. Assume change every 4 months (3 times each year)
Garment/support - sleeve & gloves	Garment	4	One off cost	1	Daily	1	30.27	30.27	NHS Drug Tariff pag 625 Sigvaris Advance 2 Arms Sleeve with Hand Piece and Grip Top. Medium compression. Assume change every 4

									months (3 times each year)
Garment/support - socks	Garment	4	One off cost	1	Daily	1	28.74	28.74	NHS Drug Tariff pag 625 Sigvaris Active Work Wear Sock (pair price).Assume change every 4 months (3 times each year)
Garment/support (undefined)	Garment	4	One off cost	1	Daily	1	114.77	114.77	NHS Drug Tariff Gloves + Sleeve + socks (not specific). Assume change every 4 months (3 times each year)
Gaviscon	Antacid	7	One off cost	5ml four times	Daily	4	1.22	0.01	Gaviscon Advance or eqv - 500ml - Sodium alginate 500mg/5ml / Potassium bicarbonate 100mg/5ml oral suspension sugar free 500

									ml / Packsize 1 (eMIT database)
Glucosamine	Joints	730	One off cost	1250mg	Daily	1.25	4.25	0.05	90 tablets (1000mg) FSC Glucosamine sulfate 100mg tablets - dosage for Alateris https://bnf.nice.org.uk/drugs/glucosamine/medicinal-forms/
Glyceryl trinitrate	Cardiac treatment	Until end of trial	If start date given, but no stop date, assume end of trial	500mg (1 tablet) before the activity	Daily	1	4.78	0.05	Glyceryl trinitrate 500microgram sublingual tablets / Packsize 100 (eMIT database)
Homeopathy	Homeopathy	180	One off cost	1	Daily	1	10.3	0.343333	Boots Menolieve Black Cohosh root extract 6.5 mg - 30 tablets. Assume 1 tablet per day https://www.boots.com/boots-menolieve-black-cohosh-root-extract-6-5mg-30-

									tablets-10086726
Hormone replacement therapy	HRT	24	One off cost	10mg	Daily	1	10.6	0.44	Estradiol 10microgram pessaries/vaginal tablets (e.g. vagifem) / Packsize 24 (eMIT database)
Hux D3	Vitamin	Until end of trial	If start date given, but no stop date, assume end of trial	4000units	Daily	1.25	8.99	0.1	Hux D3 3,200 unit capsules https://bnf.nice.org.uk/drugs/colecalciferol/medicinal-forms/
Hydrocortisone cream	Cream	One off	One off cost	1	One-off	1	3.26	3.26	Hydrocortisone 0.5% cream https://bnf.nice.org.uk/drugs/hydrocortisone/medicinal-forms/#cutaneous-cream
Hydrocortisone with miconazole	Steroids	One off	One off cost	1	One-off	1	2.49	2.49	Daktacort 2%1% cream 30gr https://bnf.nice.org.uk/drugs/hydrocortisone-with-

									miconazole/medicinal-forms/
Hydroxychloroquine	Rheumatoid Arthritis	Until end of trial	If start date given, but no stop date, assume end of trial	200mg	Daily	1	3.04	0.05	Hydroxychloroquine 200mg tablets / Packsize 60 (eMIT database)
Hylo Forte Eye Drops	Eye lubricant	One off	One off cost	Apply as required	One-off	1	9.5	9.5	Hylo-Forte 0.2% eye drops preservative free https://bnf.nice.org.uk/drugs/sodium-hyaluronate/medicinal-forms/
Hypromellose Eye Drops	Eye lubricant	One off	One off cost	1 drop 3 times	One-off	1	4.99	4.99	10 ml Hypromellose 0.3% eye drops Alissa Healthcare Research https://bnf.nice.org.uk/drugs/hypromellose/medicinal-forms/
Ibandronic acid	Osteoporosis	Until end of trial	If start date given, but no stop date, assume end of trial	50mg	Daily	1	27.13	0.97	Ibandronic acid 50mg tablets / Packsize 28 (eMIT database)

Ibuprofen	Pain relief	2	One off cost	200mg three times	Daily	3	0.41	0.017083	Ibuprofen 200mg tablets / Packsize 24 (eMIT database)
Ibuprofen gel	Pain relief	One off	One off cost	3 times	One-off	1	0.59	0.59	Ibuprofen 5% gel 50 gram / Packsize 1 (eMIT database)
Indapamide	Antihypertensive	Until end of trial	If start date given, but no stop date, assume end of trial	2.5mg	Daily	1	0.56	0.02	Indapamide 2.5mg tablets / Packsize 28 (eMIT database)
Injection (undefined)	Injection	Until end of trial	If start date given, but no stop date, assume end of trial	60mg	Half-yearly	1	183	183	Prolia solution for injection pre-filled syringes https://bnf.nice.org.uk/drugs/denosumab/medicinal-forms/ Dosage from https://bnf.nice.org.uk/drugs/denosumab/#indications-and-dose
Isosorbide	Cardiac treatment	Until end of trial	If start date given, but no stop date,	10mg twice daily	Daily	0.33	1.4	0.06	Isosorbide mononitrate 60mg

			assume end of trial						modified-release tablets / Packsize 28 (eMIT database)
Ivabradine	Cardiac treatment	Until end of trial	If start date given, but no stop date, assume end of trial	5mg twice	Daily	2	22.19	0.4	Ivabradine 5mg tablets / Packsize 56 (eMIT database)
Lactulose	Laxative	7	One off cost	15mL twice	Daily	0.03	1.6	1.6	Lactulose 3.1-3.7g/5ml oral solution 500 ml / Packsize 1 https://www.nhs.uk/medicines/lactulose/how-and-when-to-take-lactulose/#:~:text=You%20can%20take%20lactulose%20for,take%20it%20for%20many%20months. Usual duration 1 week
Lamotrigine	Anticonvulsant	Until end of trial	If start date given, but no stop date,	100mg	Daily	2	3.75	0.07	56 tablets (50mg) A A H Pharmaceuticals -

			assume end of trial						maintenance dosage https://bnf.nice.org.uk/drugs/lamotrigine/medicinal-forms/
Lansoprazole	Gastrointestinal treatment	30	One off cost	30mg	Daily	1	0.58	0.020714	Lansoprazole 30mg gastro-resistant capsules / Packsize 28 (eMIT database)
Latanoprost	Glaucoma	One off	One off cost	1	One-off	1	1.34	1.34	Latanoprost 50micrograms/ml eye drops 2.5 ml / Packsize 1 (eMIT database)
Levothryoxine	Thryoid hormone replacement	Until end of trial	If start date given, but no stop date, assume end of trial	50mg	Daily	2	0.43	0.02	Levothyroxine sodium 25microgram tablets / Packsize 28 (eMIT database)
Lidocaine	Local anesthetic	One off	One off cost	Up to 8 sprays	One-off	1	16.02	16.02	Lidocaine 5% / Phenylephrine 0.5%nasal spray 2.5 ml (Co-Phenylcaine Forte) / Packs

									ize 1 (eMIT database)
Lidocaine plasters	Pain relief	One off	One off cost	1	One-off	1	61.54	2.05	Ralvo 700mg medicated plasters https://bnf.nice.org.uk/drugs/lidocaine-hydrochloride/medicinal-forms/
Lisinopril	Cardiac treatment	Until end of trial	If start date given, but no stop date, assume end of trial	20mg	Daily	4	0.52	0.02	Lisinopril 5mg tablets / Packsize 28 (eMIT database)
Loperamide	Antidiarrheal	1	One off cost	4mg	Daily	2	0.41	0.01	Loperamide 2mg capsules (standard pack) / Packsize 30 (eMIT database)
Loprazolam	Sleeping tablet	30	One off cost	1mg	Daily	1	27	0.96	28 tablets (1mg) A A H Pharmaceuticals
Losartan	Cardiac treatment	Until end of trial	If start date given, but no stop date, assume end of trial	50mg	Daily	2	0.49	0.02	Losartan 25mg tablets / Packsize 28 (eMIT database)

Lymphoedema clinic	Chsc treatment	2	One off cost	0	Daily	1	40.9	40.9	Specialist nurse lymphoedema clinic PSSRU
Massage	Chsc treatment	3	One off cost	0	Daily	1	21.09	21.09	Physiotherapist face to face PSSRU
Mebeverine	Treats muscle spasms	14	One off cost	200mg	Daily	1.5	4.85	0.05	Mebeverine 135mg tablets / Packsize 100 (eMIT database)
Medicated patches (undefined)	Medicated patches (undefined)	One off	One off cost	1	One-off	1	61.54	2.05	Ralvo 700mg medicated plasters https://bnf.nice.org.uk/drugs/lidocaine-hydrochloride/medicinal-forms/
Medroxyprogesterone	Hormonal treatment	70	One off cost	20mg	Daily	2	24.73	0.25	100 tablets https://bnf.nice.org.uk/drugs/medroxyprogesterone-acetate/medicinal-forms/ (10mg) Dosage for hot flushes

Megestrol	Appetite stimulant	60	One off cost	160mg	Daily	1	19.52	0.65	30 tablets (160mg) https://bnf.nice.org.uk/drugs/megestrol-acetate/medicinal-forms/
Meloxicam	Pain relief	7	One off cost	15mg	Daily	2	1.54	0.05	Meloxicam 7.5mg tablets / Packsize 30 (eMIT database)
Metformin	Diabetes	Until end of trial	If start date given, but no stop date, assume end of trial	500mg 3 times	Daily	3	0.33	0.01	Metformin 500mg tablets / Packsize 28 (eMIT database)
Metoclopramide	Sickness	5	One off cost	10mg	Daily	1	0.72	0.03	Metoclopramide 10mg tablets / Packsize 28 (eMIT database)
Mindfulness	Chsc treatment	4	One off cost	0	Daily	1	27.27	27.27	Counsellor face to face visit PSSRU
Mirabegron	Overactive bladder treatment	Until end of trial	If start date given, but no stop date, assume end of trial	50mg	Daily	1	29	0.97	Betmiga 50mg modified-release tablet https://bnf.nice.org.uk/drugs/

									mirabegron/medicinal-forms/
Mirtazapine	Antidepressant	Until end of trial	If start date given, but no stop date, assume end of trial	15mg	Daily	1	0.57	0.02	Mirtazapine 15mg tablets / Packsize 28 (eMIT database)
Mometasone	Steroids	One off	One off cost	1	One-off	1	1.14	1.14	Mometasone 0.1% ointment 30 gram / Packsize 1 (eMIT database)
Morphine MST	Pain relief	3	One off cost	10mg every 4 hours	Daily	6	5.2	0.09	60 tablets (10mg) Napp Pharmaceuticals Ltd https://bnf.nice.org.uk/drugs/morphine/medicinal-forms/
Mouth product (undefined)	Mouth product	One off	One off cost	1	One-off	1	3.5	3.5	Saliveze mouth spray https://bnf.nice.org.uk/medical-devices/artificial-saliva-products/saliveze/
Movicol	Constipation	14	One off cost	4 sachets	Daily	4	1.97	0.0985	Macrogol compound oral

									powder sachets NPF sugar free (orange) (e.g. Laxido, Macroherm,Movicol,cosmocol,laxagol,macilax) / Packsize 20 (eMIT database)
Multivitamins supplements	Vitamin	Until end of trial	If start date given, but no stop date, assume end of trial	1	Daily	1	4.9	0.05	Tesco A-Z Multivitamins & Minerals 90S https://www.tesco.com/groceries/en-GB/products/285513527
Naproxen	Anti-inflammatory	1	One off cost	500mg	Daily	2	0.59	0.02	Naproxen 250mg tablets / Packsize 28 (eMIT database)
Natecal D3	Vitamin	Until end of trial	If start date given, but no stop date, assume end of trial	2 tablets	Daily	2	3.63	0.06	60 tablets https://bnf.nice.org.uk/drugs/colecalciferol-with-calcium-carbonate/medicinal-forms/ Dosage from https://www.m

									edicines.org.uk/emc/product/6313/smpc#about-medicine
Nebivolol	Cardiac treatment	Until end of trial	If start date given, but no stop date, assume end of trial	5mg daily	Daily	1	0.79	0.03	Nebivolol 5mg tablets / Packsize 28 (eMIT database)
Nefopam	Pain relief	3	One off cost	30mg 3 times	Daily	3	3.67	0.04	Nefopam 30mg tablets / Packsize 90 (eMIT database)
Nitrofurantoin	Antibiotic	4	One off cost	50mg 4 times a day	Daily	2	9.77	0.7	Nitrofurantoin 100mg modified-release capsules / Packsize 14 (eMIT database)
Nortriptyline	Antidepressant	Until end of trial	If start date given, but no stop date, assume end of trial	10mg	Daily	1	0.85	0.01	Nortriptyline 10mg tablets / Packsize 100 (eMIT database)
Occupational therapist	Chsc treatment	4	One off cost	0	Daily	1	27.27	27.27	Counsellor face to face visit PSSRU
Omeprazole	Gastrointestinal treatment	30	One off cost	20mg	Daily	2	0.35	0.01	Omeprazole 20mg gastro-resistant

									capsules / Packsize 28 (eMIT database)
Oramorph	Pain relief	3	One off cost	10mg every 4 hours	Daily	6	1.89	0.09	100ml each solution has a size of 5ml (20 solutions). Recommended dose for acute pain is 60mg per day (10mg every 4 hours) https://bnf.nice.org.uk/drugs/morphine/#unlicensed-use
Ovestin cream	Menopause treatment	One off	One off cost	1	One-off	1	5.45	5.45	15gram https://bnf.nice.org.uk/drugs/estriol/medicinal-forms/
Pain killers (undefined)	Pain relief	2	One off cost	500mg four times	Daily	4	0.49	0.015313	Paracetamol 500mg tablets / Packsize 32 (eMIT database)
Panadol	Pain relief	3	One off cost	1000mg	Daily	2	1.45	0.09	Panadol Advance 500mg Paracetamol - 16 Tablets https://www.bo

									ots.com/panadol-advance-500mg-paracetamol---16-tablets-10323679?cm_mmc=bmm-buk-google-ppc-_-PLAs_HeroCompare-_--_-PMax:+UK_Smart_Shopping_Healthcare_Other&gad_source=1&gclid=CjwKCAjw74e1BhBnEiwAbqOAjH-NsnPA9sVCSAGEcInI1vPDOresnCUvi04UAkwfrmXMhCH7UdcHWxoCZ8YQAvD_BwE&gclsrc=aw.ds
Paracetamol	Pain relief	2	One off cost	500mg four times	Daily	4	0.49	0.02	Paracetamol 500mg tablets / Packsize 32 (eMIT database)

Paroxetine	Antidepressant	Until end of trial	If start date given, but no stop date, assume end of trial	20mg	Daily	1	0.68	0.02	Paroxetine 20mg tablets / Packsize 30 (eMIT database)
Penicillin	Antibiotic	5	One off cost	250mg twice	Daily	2	1.18	0.042	Phenoxymethylpenicillin 125mg/5ml oral solution 100 ml / Packsize 1 Assume same as phenoxymethylpenicillin as they are both prescribed for lymphoedema (eMIT database)
Perindropril	Cardiac treatment	Until end of trial	If start date given, but no stop date, assume end of trial	4mg	Daily	1	0.41	0.01	Perindopril erbumine 4mg tablets / Packsize 30 (eMIT database)
Phenoxymethylpenicillin	Antibiotic	5	One off cost	250mg twice	Daily	2	1.18	0.04	Phenoxymethylpenicillin 250mg tablets / Packsize 28 (eMIT database)

Physiotherapy	Chsc treatment	3	One off cost	0	Daily	1	21.09	21.09	Physiotherapist face to face PSSRU
Pravastatin	Cardiac treatment	Until end of trial	If start date given, but no stop date, assume end of trial	40mg	Daily	2	0.66	0.02	Pravastatin 20mg tablets / Packsize 28 (eMIT database)
Pregabalin	Pain relief	30	One off cost	150mg	Daily	6	0.68	0.01	Pregabalin 25mg capsules / Packsize 56 (eMIT database)
Premarin tablets	Menopause treatment	Until end of trial	If start date given, but no stop date, assume end of trial	0.3mg	Daily	1	24.02	0.285952	Premarin 0.3mg tablets Pfizer https://bnf.nice.org.uk/drugs/conjugated-oestrogens-equine/medicinal-forms/
Prochlorperazine	Sickness	2	One off cost	10mg	Daily	2	0.86	0.03	Prochlorperazine 5mg tablets / Packsize 28 (eMIT database)
Promethazine	Anti-allergy	One off	One off cost	25mg	One-off	1	18.01	0.32	56 tablets (25mg) A A H Pharmaceuticals https://bnf.nice

									.org.uk/drugs/promethazine-hydrochloride/medicinal-forms/
Propranolol	Cardiac treatment	Until end of trial	If start date given, but no stop date, assume end of trial	40mg	Daily	4	0.33	0.01	Propranolol 10mg tablets / Packsize 28 (eMIT database)
Pyridoxine	Vitamin	Until end of trial	If start date given, but no stop date, assume end of trial	20mg	Daily	0.4	14.76	0.53	Pyridoxine 50mg tablets / Packsize 28 (eMIT database)
Quinine	Antimalarial	30	One off cost	300mg	Daily	1.5	4.42	0.16	Quinine sulfate 200mg tablets / Packsize 28 (eMIT database)
Ramipril	Cardiac treatment	Until end of trial	If start date given, but no stop date, assume end of trial	1.25mg	Daily	0.5	0.36	0.01	Ramipril 2.5mg capsules / Packsize 28 (eMIT database)
Ranitidine	Gastrointestinal treatment	30	One off cost	300mg	Daily	1	5.99	0.21	Assume 28 tablets (300mg) Unknown price, not reported in the BNF

									https://www.nhsbsa.nhs.uk/pharmacies-gp-practices-and-appliance-contractors/drug-tariff/drug-tariff-updates
Replens	Vaginal moisturizer	One off	One off cost	1	One-off	1	6.06	6.06	35g tube - NHS Drug Tariff https://www.drugtariff.nhsbsa.nhs.uk/#/00852760-DD/DD00852626/Part%20IXA-Appliances
Risedronate	Osteoporosis	Until end of trial	If start date given, but no stop date, assume end of trial	5mg	Daily	0.142857	0.4	0.1	Risedronate sodium 35mg tablets / Packsize 4 (eMIT database)
Rivaroxaban	Cardiac treatment	35	One off cost	20mg	Daily	2	18	1.8	10 tablets (10mg) https://bnf.nice.org.uk/drugs/rivaroxaban/medicinal-forms/ Dosage https://bnf.nice

									.org.uk/drugs/rivaroxaban/
Ropinirole	Parkinson's disease treatment	Until end of trial	If start date given, but no stop date, assume end of trial	2mg	Daily	0.5	15.17	0.18	Ropinirole 1mg tablets / Packsize 84 (eMIT database)
Salamol inhaler	Asthma inhaler	Until end of trial	If start date given, but no stop date, assume end of trial	100mg	Daily	1	1.4	1.4	Salbutamol 100micrograms/dose inhaler CFC Free 200 dose / Packsize 1 (eMIT database)
Salbutamol	Asthma inhaler	Until end of trial	If start date given, but no stop date, assume end of trial	100mg	Daily	1	1.4	1.4	Salbutamol 100micrograms/dose inhaler CFC Free 200 dose / Packsize 1 (eMIT database)
Sertraline	Antidepressant	Until end of trial	If start date given, but no stop date, assume end of trial	50mg	Daily	1	0.45	0.02	Sertraline 50mg tablets / Packsize 28 (eMIT database)
Simvastatin	Cardiac treatment	Until end of trial	If start date given, but no stop date, assume end of trial	20mg	Daily	0.25	0.36	0.01	Simvastatin 40mg tablets / Packsize 28 (eMIT database)

Solpadol	Pain relief	3	One off cost	2 tablets every 4 hours	Daily	6	6.74	0.07	100 tablets https://bnf.nice.org.uk/drugs/co-codamol/medicinal-forms/
Spiriva inhalation powder	Asthma inhaler	Until end of trial	If start date given, but no stop date, assume end of trial	5mg	Daily	2	23	0.383333	Spiriva Respimat 2.5micrograms/dose inhalation solution cartridge with device 60 doses (eMIT database)
Spironolactone	Cardiac treatment	Until end of trial	If start date given, but no stop date, assume end of trial	25mg	Daily	1	1.39	0.05	Spironolactone 25mg tablets / Packsize 28 (eMIT database)
Statins (undefined)	Cardiac treatment	Until end of trial	If start date given, but no stop date, assume end of trial	10mg	Daily	0.25	0.68	0.02	Atorvastatin 40mg tablets / Packsize 28 (eMIT database)
Stemetil	Sickness	2	One off cost	10mg	Daily	2	0.8	0.08	Prochlorperazine 5mg tablets / Packsize 28 (eMIT database)

Steroid cream (undefined)	Steroids	One off	One off cost	1	One-off	1	2.56	2.56	ClobaDerm 0.05% cream https://bnf.nice.org.uk/drugs/clobetasol-propionate/medicinal-forms/
Steroid injections (undefined)	Steroids	1	One off cost	100mg	Daily	1	9.28	0.93	Hydrocortisone sodium succinate 100mg powder for solution for injection vials (e.g. Solu-Cortef or eqv) / Packsize 10 (eMIT database)
Steroids (undefined)	Steroids	7	One off cost	10mg	Daily	0.5	0.41	0.01	Prednisolone 5mg tablets / Packsize 28 (eMIT database)
Strontium ranelate	Osteoporosis	Until end of trial	If start date given, but no stop date, assume end of trial	2g	Daily	1	147.32	5.26	Strontium ranelate 2g granules sachets sugar free / Packsize 28 (eMIT database)
Sulfasalazine	Rheumatoid Arthritis	Until end of trial	If start date given, but no	500mg	Daily	1	8.62	0.08	Sulfasalazine 500mg gastro-

			stop date, assume end of trial						resistant tablets / Packs ize 112 (eMIT database)
Sylk lubricant	Vaginal moisturizer	One off	One off cost	1	One-off	1	5.16	5.16	NHS Electronic Drug Tariff https://www.drugtariff.nhsbsa.nhs.uk/#/00548381-DA/DA00547781/Part%20IXA-Appliances
Systane Eye Drops	Eye lubricant	One off	One off cost	1	One-off	1	7.68	7.68	NHS Electronic Drug Tariff http://www.drugtariff.nhsbsa.nhs.uk/#/00371022-DD/DD00370113/Part%20IXA-Appliances
Tens machine	Pain relief device	One off	One off cost	1	One-off	1	59.99	59.99	https://www.boots.com/boots-tens-digital-pain-relief-10165899
TheiCal-D3	Vitamin	Until end of trial	If start date given, but no stop date,	1000mg	Daily	1	2.95	0.1	30 tablets https://bnf.nice.org.uk/drugs/c

			assume end of trial						olecalciferol-with-calcium-carbonate/medicinal-forms/ Dosage from https://www.medicines.org.uk/emc/product/3334/pil#gref
Tildiem	Cardiac treatment	Until end of trial	If start date given, but no stop date, assume end of trial	200mg	Daily	1	6.29	0.22	28 capsules (200mg) https://bnf.nice.org.uk/drugs/diltiazem-hydrochloride/medicinal-forms/
Timolol	Glaucoma	One off	One off cost	1	One-off	1	3.37	3.37	Timolol 5mg/ml / Latanoprost 50micrograms/ml eye drops 2.5 ml (e.g. Xalacom) / Packsize 1 (eMIT database)
Topical oestrogen cream (undefined)	Menopause treatment	One off	One off cost	1	One-off	1	4.52	4.52	Estriol 0.1% cream 15 gram (1mg/g) / Packsize 1 (eMIT database)

Traditional Chinese Therapy	Chsc treatment	3	One off cost	0	Daily	1	56.06	56.06	Physiotherapist face to face (band 6) PSSRU
Tramadol	Pain relief	3	One off cost	50mg	Daily	1	0.51	0.02	Tramadol 50mg capsules / Packsize 30 (eMIT database)
Treatment for dry eyes (undefined)	Eye lubricant	One off	One off cost	1	One-off	1	4.99	4.99	10 ml Hypromellose 0.3% eye drops Alissa Healthcare Research https://bnf.nice.org.uk/drugs/hypromellose/medicinal-forms/
Trimethoprim	Antibiotic	3	One off cost	200mg twice daily	Daily	2	0.38	0.03	Trimethoprim 200mg tablets / Packsize 14 (eMIT database)
Vagifem	Menopause treatment	39	One off cost	1	Daily	1	16.72	0.7	24 pessaries Vagifem 10microgram vaginal tablets https://bnf.nice.org.uk/drugs/e

									stradiol/medicinal-forms/
Vaginal lubricant (undefined)	Vaginal moisturizer	One off	One off cost	1	One-off	1	6.06	6.06	35g tube - NHS Durg Tariff https://www.drugtariff.nhsbsa.nhs.uk/#/00852760-DD/DD00852626/Part%20IXA-Appliances
Vaginal pessary	Vaginal support device	39	One off cost	once a day for 3 weeks	One-off	1	13.38	0.56	Imvaggis 0.03mg pessaries https://bnf.nice.org.uk/drugs/estriol/medicinal-forms/
Venlafaxine	Antidepressant	Until end of trial	If start date given, but no stop date, assume end of trial	37.5mg	Daily	0.5	1.74	0.06	Venlafaxine 75mg modified-release capsules / Packsize 28 (eMIT database)
Ventolin	Asthma inhaler	Until end of trial	If start date given, but no stop date, assume end of trial	200mg	Daily	1	1.99	0.03	60 doses (200mg) https://bnf.nice.org.uk/drugs/salbutamol/medicinal-forms/

Vitamin B supplement (undefined)	Vitamin	Until end of trial	If start date given, but no stop date, assume end of trial	100mg	Daily	1	0.78	0.03	Thiamine 100mg tablets / Packsize 28 (eMIT database)
Vitamin C+D supplement (undefined)	Vitamin	Until end of trial	If start date given, but no stop date, assume end of trial	1	Daily	1	4.32	0.07	Boots Vitamin C + Vitamin D, 60 tablets https://www.boots.com/boots-vitamin-c-and-vitamin-d-food-supplement-60-tablets-10291401
Vitamin D supplement (undefined)	Vitamin	Until end of trial	If start date given, but no stop date, assume end of trial	400 units	Daily	0.5	1.26	0.04	Colecalciferol 800unit capsules / Packsize 30 [Maintenance dose for treatment D deficiency] (eMIT database)
Vitamin E supplement	Vitamin	Until end of trial	If start date given, but no stop date, assume end of trial	3mg daily	Daily	1	1	0.03	Valupak Vitamin E 100unit capsules https://bnf.nice.org.uk/drugs/alpha-

									tocopheryl-acetate/medicinal-forms/
Warfarin	Cardiac treatment	Until end of trial	If start date given, but no stop date, assume end of trial	5mg once daily	Daily	5	0.59	0.02	Warfarin 1mg tablets / Packsize 28 (eMIT database)
Zapain	Pain relief	3	One off cost	8mg/500mg * 6 hours *4	Daily	4	1.56	0.0312	50 tablets https://bnf.nice.org.uk/drugs/co-codamol/medicinal-forms/
Zerobase cream	Cream	One off	One off cost	1	One-off	1	1.1	1.1	Zerobase 11% cream, 50gr https://www.chemist-4-u.com/zerobase-emollient-cream-50g?gad_source=1&gclid=CjwKCAiAhP67BhAVEiwA2E_9g5z3O-Sqf86B-XO249Mb5P6eisxVsMKUQRZjd3SiTbWppSnP5Ao0Wx

									oCFJUQAvD_BwE
Zoledronic acid	Prevent bone fractures in those with advanced cancer	One off	One off cost	5mg once yearly	One-off	1	5.04	5.04	Zoledronic acid 4mg/5ml solution for injection vials / Packsize 1 (eMIT database)
Zomorph	Pain relief	3	One off cost	10mg every 4 hours	Daily	6	3.47	0.06	60 capsules(10mg) https://bnf.nice.org.uk/drugs/morphine/medicinal-forms/
Zopiclone	Sleeping tablet	30	One off cost	3.75mg	Daily	1	0.52	0.02	Zopiclone 3.75mg tablets / Packsize 28 (eMIT database)

Supplementary table 2. 3 Recommended dosage, duration and unit cost of non-prescribed medications

Name unprescribed medication	Category	Duration (days)	Assumption	Dosage	Dosage frequency	Number of dosages per frequency	Cost package (£)	Unit cost (£)	Sources and details
Anadin Pain Relief	Pain relief	2	One off cost	2 tablets	Daily	2	2.4	0.15	https://www.boots.com/anadin-extra-caplets-16-10191782
Apple Cider Vinegar	Supplement	Until end of trial	If start date given, but no stop date, assume end of trial	3	Daily	3	18	0.3	https://www.boots.com/myvitamins-apple-cider-vinegar-gummies-apple-60-gummies-10342709
Apricot Kernels	Supplement	30	One off cost	1	Daily	1	7.95	0.02	https://everydaysuperfood.co.uk/products/apricot-kernels?variant=42666519756990&currency=GBP&utm_medium=product_sync&utm_source=google&utm_content=sag_organic&utm_campaign=sag_organic&gad_source=1&gclid=Cj0KCQjw1Yy5BhD-ARIsAI0RbXYv8aUJkXUQVBkxVGE4JaOQyNvwv-H7m6YxqZHb0X8YmB5yrv41obQaAvMbEALw_wcB
Arnica	Cream	One off	One off cost	1	One-off	1	4.5	4.5	https://www.boots.com/boots-bruise-relief-

									arnica-cream-10258294?srsltid=AfmBOoowmNI7vFThD2q0ntnY489ecKaGHm-tz_6tUB_lAryxD7k2Li-p
ASEA supplement	Supplement	1	One off cost	1	One-off	1	180	180	https://www.breathe360.uk/products/asea-redox-signalling?srsltid=AfmBOoopIZ0BVIN412iV6OCuCGU0EvFh3bNY3QamwOK_MyD5uVfn5wKa
Bellis Perennis	Homeopathy	30	One off cost	2	Daily	2	12.75	0.01	https://www.rxhomeo.com/bellis-perennis-30c-1000-pellets-homeopathic-remedy/
Beta glucan	Cream	One off	One off cost	1	One-off	1	12	12	https://www.boots.com/the-ordinary-natural-moisturizing-factors-beta-glucan-100ml-10329958?srsltid=AfmBOorWfoeO_cCPVj41zsarEZEE3D19DhbBZEvSoAHmKhV1072KRyEG
Bio-Oil	Cream	One off	One off cost	1	One-off	1	23.99	23.99	https://www.boots.com/bio-oil-200ml-for-scars-stretch-marks-and-dehydrated-skin-10072167

Biotin supplement	Supplement	Until end of trial	If start date given, but no stop date, assume end of trial	1	Daily	1	14	0.16	https://www.boots.com/boots-hair-formula-biotin-90-tablets-10326826?cm_mmc=bmm-buk-google-ppc-_-PLAs_HeroCompare-_--_-pmax_health__gg_shopping__hc-vitssupps___pmedia&gad_source=1&gclid=Cj0KCQjw1Yy5BhD-ARIsAI0RbXaOBRIvpaiu4P7XQVlQ9YiWy8hDqR4EwKQEwW0T31b3OGVxtAv4OnwaAtYPEALw_wcB&gclsrc=aw.ds
Black cohosh tablets	Supplement	180	One off cost	1	Daily	1	10.3	0.34	https://www.boots.com/boots-menolieve-black-cohosh-root-extract-6-5mg-30-tablets-10086726?srsltid=AfmBOoqXwbtDQg6dc7FtSu0QbFxXWXd1v5xE57ZxKkZkUam_LUC_4uej
Bowen treatment	Chsc treatment	3	One off cost	0	Daily	1	56.06	56.06	Physiotherapist face to face (band 6) PSSRU
Calcium supplement (undefined)	Vitamin	Until end of trial	If start date given, but no stop date,	150mg twice	Daily	0.17	12.31	0.41	Calcium lactate gluconate 2.327g / Calcium carbonate 1.75g effervescent

			assume end of trial						tablets sugar free (Sandocal 1000) / Packsize 30 (eMIT database)
Carcinosin	Homeopathy	30	One off cost	2	Daily	1	4.3		https://www.homeoforce.co.uk/carcinosin-20410-p.asp https://homeopathinfo.com/carcinosin/
CBD	CBD	Until end of trial	If start date given, but no stop date, assume end of trial	1 spray	Daily	1	10	0.03	https://www.boots.com/vitality-cbd-oral-spray-high-strength-lemon-flavour-10266363
CBD oil	CBD	Until end of trial	If start date given, but no stop date, assume end of trial	1	Daily	1	14.49	0.04	https://www.boots.com/healthspan-cbd-oil-260mg-drops-275--10ml-10263817
Claritin	Anti-allergy	Until end of trial	If start date given, but no stop date, assume end of trial	1	Daily	1	10.99	0.37	https://www.boots.com/clarityn-allergy-10mg-tablets-30s-10314786?srsltid=AfmBOoq2xqBZk64V2g3MQYJ6RRNZzUnO3Mq2Gr66S-Hkf5f1gnRF97tE
Cod liver oil	Supplement	Until end of trial	If start date given, but no stop date,	1	Daily	1	3.5	0.12	https://www.boots.com/boots-omega-3-fish-oil---30-capsules-10259788

			assume end of trial						
Co-enzyme Q10 supplement	Supplement	Until end of trial	If start date given, but no stop date, assume end of trial	1	Daily	1	9	0.3	https://www.boots.com/boots-re-balance-vitality-co-q10-50-mg-30-capsules-10149733
Cranberry extract	Supplement	180	One off cost	1	Daily		4	0.13	https://www.boots.com/boots-cranberry-extract-400mg-30-tablets-10151464?cm_mmc=bmm-buk-google-ppc-_-PLAs_HeroCompare-_--_-pmax_health__gg_shopping__hc-vitssupps___pmedia&gad_source=1&gclid=Cj0KCQjw1Yy5BhD-ARIsAI0RbXb88vJ6MyLMk2ECKJu23eynWsyUG2VZvBWMojhgPsbnVjOPE0ePK4caAlSrEALw_wcB&gclsrc=aw.ds
Curcumin supplement	Supplement	90	https://www.webmd.com/vitamins/ai/ingredientmono-662/turmeric	1	Daily	1	28	0.93	https://www.boots.com/bioglan-active-curcumin-tablets-30s-10253906?srsltid=AfmBOoodS_K4dlssTxly9sl l6hDC7OhJnVknsr2CzL7NRhgZ2wtC9ENt

Doxazosin	Hypertension	Until end of trial	If start date given, but no stop date, assume end of trial	1	Daily	1	1.11	0.04	https://bnf.nice.org.uk/drugs/doxazosin/medicinal-forms/
Essential oils	Cream	One off	One off cost	1	One-off	1	11	11	https://www.boots.com/botanics-peaceful-night-pure-essential-oil-lavender-20ml-10264131
Treatment for dry eyes (undefined)	Eye lubricant	One off	One off cost	1	One-off	1	4.99	4.99	10 ml Hypromellose 0.3% eye drops Alissa Healthcare Research https://bnf.nice.org.uk/drugs/hypromellose/medicinal-forms/
Feverfew	Anti-inflammation	60	One off cost	1	Daily	1	8	0.27	https://www.boots.com/boots-migraine-relief-feverfew-herb-100mg-30-capsules-10050597?srsltid=AfmBOorwpVP4O83LWx_XvCxPH2jXM6na2Ju5tpJC8GfUSuMi3BKSPk7l
Flaxseed	Anti-inflammation	90	One off cost	1	Daily	1	10.99	0.18	https://www.hollandandbarrett.com/shop/product/holland-barrett-flaxseed-oil-vegi-capsules-500mg-60080493?skuid=060312&utm_campaign=shop

									ping&utm_medium=cpc&utm_source=google&utm_campaign=pmax&&gad_source=1&gclid=Cj0KCQjw1Yy5BhD-ARIsAI0RbXanPDCk-kh336dJX2Dkkk1-7ug9FeiqgJnwNlXT2b2n0IWs8IFWQE8aAkzNEALw_wcB&gclsrc=aw.ds
Garlic supplement	Supplement	90	One off cost	1	Daily	1	7.5	0.08	https://www.boots.com/boots-garlic---90-tablets-10270051?cm_mmc=bmm-buk-google-ppc-_-PLAs-_--_-pmax_catchall__gg_shopping__v2catchall___pmedia&gad_source=1&gclid=Cj0KCQjw1Yy5BhD-ARIsAI0RbXbFMKut6CesdjL20wp3oc4GBdEihSPJ7X5SKj_4D6XuV4kOFxykQIkaAn66EALw_wcB&gclsrc=aw.ds
Garment/support (undefined)	Garment	4	One off cost	1	Daily	1	114.77	114.77	NHS Drug Tariff Gloves + Sleeve + socks (not specific). Assume change every 4 months (3 times each year)

Ginger supplement	Supplement	Until end of trial	If start date given, but no stop date, assume end of trial	1	Daily	1	6	0.1	https://www.boots.com/boots-naturals-ginger-60-tablets-10275606?cm_mmc=bmm-buk-google-ppc-_-PLAs_HeroCompare-_--_-pmax_health__gg_shopping__hc-vitssupps___pmedia&gad_source=1&gclid=Cj0KCQjw1Yy5BhD-ARIsAI0RbXb9b_guqfQS-A_iRDySeq_KwY82QrULlMTiaNjksi4wW-5pwPVEky0aAsv9EALw_wcB&gclsrc=aw.ds
Ginkgo biloba	Supplement	Until end of trial	If start date given, but no stop date, assume end of trial	1	Daily	1	25.7	0.29	https://www.boots.com/boots-ginkgo-biloba-120mg-90-tablets--10274982?cm_mmc=bmm-buk-google-ppc-_-PLAs-_--_-pmax_catchall__gg_shopping__v2catchall___pmedia&gad_source=1&gclid=Cj0KCQjw1Yy5BhD-ARIsAI0RbXaY6wIMjTWzHDY-pCM8svrBOtFDeCbtZ5al_pUwima-iAH-

									KyCfMCkaAkhREALw_wcB&gclsrc=aw.ds
Green tea	Supplement	Until end of trial	If start date given, but no stop date, assume end of trial	1	Daily	1	12.05	0.12	https://www.hollandandbarrett.com/shop/product/nature-s-garden-green-tea-extract-750mg-100-tablets-6100003921?skuid=064400&utm_campaign=shopping&utm_medium=cpc&utm_source=google&utm_campaign=pmax&&gad_source=1&gclid=Cj0KCQjw1Yy5BhD-ARIsAI0RbXad6lEnYFiGVxz4n3WpYG5MdNoE6MZMXr0mbA9P-mbOKdJgemnViJEaAuHlEALw_wcB&gclsrc=aw.ds
Hair growth product (undefined)	Supplement	Until end of trial	If start date given, but no stop date, assume end of trial	1	Daily	1	14.5	0.16	https://www.boots.com/boots-beauty-formula-skin-hair-nails-90-tablets-10319613?srsltid=AfmBOoojPNl_yq8pVEVlGqsHOsvNm3FyEk0V0bZFrCh-kN_L1eZY5XN2
Herbal remedies	Vitamin	Until end of trial	If start date given, but no stop date,	60mg	Daily	1	5.31	0.09	https://www.boots.com/boots-evening-

			assume end of trial						primrose-oil-1000-mg-60-capsules-10149542
Imodium	Antidiarrheal	1	One off cost	4mg	Daily	2	3.5	0.58	https://www.boots.com/imodium-original-2mg-capsules-6-capsules-10006872
Iron supplement	Supplement	90	One off cost	1	Daily	1	1.7	0.03	https://www.boots.com/boots-iron-14mg-60-tablets-10292905?cm_mmc=bmm-buk-google-ppc-_-PLAs_HeroCompare-_--_-pmax_health__gg_shopping__hc-vitssupps___pmedia&gad_source=1&gclid=Cj0KCQjw1Yy5BhD-ARIsAI0RbXbcdj2YRtTGKQHqdNwsNkyymiLVm6bYNqGQ0jWK2IwCQiTSLnmjMGIaAuJCEALw_wcB&gclsrc=aw.ds
Kalms herbal product	Anxiety treatment	14	One off cost	6	Daily	6	9.39	0.06	https://www.boots.com/kalms-day-valerian-root-extract-25-99mg-tablets-168-tablets-10334613
KY Jelly Lubricant	Cream	One off	One off cost	1	One-off	1	4.25	4.25	https://www.boots.com/knect-personal-water-based-lube-50ml-

									10085537?utm_source=affiliates&utm_medium=CSS/Shopping%20Directory&utm_campaign=Shareight&gad_source=1&utm_source=Shareight+%28Octer%29+CSS-863943&utm_medium=affiliate&utm_campaign=Comparison+Shopping+Service+%28CSS%29&utm_term=&utm_content=&sv1=affiliate&sv_campaign_id=863943&awc=2041_1730385657_e0abe4ebeb09e4afb8b41976dd7f503f
Ladycare magnet	Vaginal support	One off	One off cost	1	One-off	1	45	45	https://www.amazon.co.uk/LadyCare-PLUS-Discreet-Menopause-alternative/dp/B00VTMZ508/ref=asc_df_B00VTMZ508/?tag=googshopuk-21&linkCode=df0&hvadid=696285193871&hvpos=&hvnetw=g&hvrand=841114020031437881 0&hvpone=&hvptwo=&hvqmt=&hvdev=c&hvdvcmdl=&hvlocint=&hvlocphy=1006656&hvtargid=pla-

									2281435177618&psc=1&mcid=5eff142ab6cd3595bd34a82fbe625e86&hvocijid=8411140200314378810-B00VTMZ508-&hvexpln=74&gad_source=1
Light therapy	Light therapy	8	One off cost	0	Daily	1	55	55	https://theskinrepairclinic.co.uk/how-much-does-redlight-near-infrared-therapy-cost/#:~:text=Price%20Range%20of%20UK%20Red%20Light%20Therapy%20Bed%20Sessions&text=Central%20London%20and%20Greater%20London,for%20a%20one%20off%20treatment.
Magnesium supplement	Supplement	Until end of trial	If start date given, but no stop date, assume end of trial	2	Daily	2	6.3	0.04	https://www.boots.com/boots-magnesium-375mg-180-tablets-%283-month-supply%29-10317532
Magnesium gel	Cream	One off	One off cost	1	One-off	1	8.62	8.62	https://www.amazon.co.uk/Better-You-P33481-BetterYou-Magnesium/dp/B06ZZX8JQS/ref=asc_df_B06ZZX8JQS/?tag=googshopuk-

									21&linkCode=df0&hvadid=696285193871&hvpos=&hvnetw=g&hvrand=6647353132118133711&hvpone=&hvptwo=&hvqmt=&hvdev=c&hvdvcmdl=&hvlocint=&hvlocphy=1006656&hvtargid=pla-2281435177618&mcid=0b78f627b0ea318b87598e5e31e98ece&hvocijid=6647353132118133711-B06ZZX8JQS-&hvexpln=74&gad_source=1&th=1
Manuka honey	Cream	One off	One off cost	1	One-off	1	10	10	https://www.boots.com/manuka-health-mgo-30-manuka-honey-blend-250g--10256528?cm_mmc=bmm-buk-google-ppc-_-PLAs_HeroCompare-_--_-pmax_health__gg_shopping__hc-vitssupps___pmedia&gad_source=1&gclid=Cj0KCQjw1Yy5BhD-ARIsAI0RbXbMXzQl0oRkM7OPQmZh4gi7uHS9STjRYJ_dSvzHz5PPVk5F4CKSGSEaAvN

									TEALw_wcB&gclsrc=aw.ds
Menoforce tablets	Menopausal symptoms treatment	30	One off cost	1	Daily	1	15.99	0.53	https://www.boots.com/a-vogel-menoforce-sage-30-tablets-10117577?srsltid=AfmBOopz4vtAhPeEA6e8k5PCFGOM9vwMTvAaWn7M86tbtbk6fX3qy5Qc
Menopace tablets	Menopausal symptoms treatment	Until end of trial	If start date given, but no stop date, assume end of trial	1	Daily	1	10	0.11	https://www.boots.com/menopace-original-90-tablets-10000298?srsltid=AfmBOooV0pUtpd9fFeyW75_crrPi_BHxzXD-yMUxJhAxPkaPpltVTnFX
Menopause supplements (undefined)	Menopausal symptoms treatment	Until end of trial	If start date given, but no stop date, assume end of trial	1	Daily	1	10	0.11	https://www.boots.com/menopace-original-90-tablets-10000298?srsltid=AfmBOooV0pUtpd9fFeyW75_crrPi_BHxzXD-yMUxJhAxPkaPpltVTnFX
Moringa Plus	Supplement	30	One off cost	2	Daily	2	131.25	2.19	https://ae.boots.com/en/buy-bio-nutrition-moringa-super-food-60-capsules.html

Mouth product (undefined)	Mouth product	One off	One off cost	1	One-off	1	3.5	3.5	Saliveze mouth spray https://bnf.nice.org.uk/medical-devices/artificial-saliva-products/saliveze/
Multivitamins supplements	Vitamin	Until end of trial	If start date given, but no stop date, assume end of trial	1	Daily	1	4.9	0.05	Tesco A-Z Multivitamins & Minerals 90S https://www.tesco.com/groceries/en-GB/products/285513527
Nytol	Sleeping treatment	14	One off cost	2	Daily	2	6.2	0.21	https://www.boots.com/wcsstore/ExtendedSitesCatalogAssetStore/Attachment%2FProduct_PatientLeaflets%2FPIL.30105.latest_Nytol_10087223.pdf
Oils (undefined)	Supplement	Until end of trial	If start date given, but no stop date, assume end of trial	1	Daily	1	3.5	0.12	https://www.boots.com/boots-omega-3-fish-oil---30-capsules-10259788
Omega 3 fish oil	Supplement	Until end of trial	If start date given, but no stop date, assume end of trial	1	Daily	1	15.99	0.09	https://www.boots.com/boots-omega-3-fish-oil-1000-mg-food-supplement-180-capsules-10149643?cm_mmc=bmm-buk-google-ppc-_-PLAs_HeroCompare-_-

									-_-pmax_health__gg_shopping__hc-vitssupps___pmedia&gad_source=1&gclid=Cj0KCQjw1Yy5BhD-ARIsAI0RbXbU4_I5XgpVbb3WWGGxvKSfCRbKCjH53hXzlezBVODUx6Yas5CSYbwaAss8EALw_wcB&gclsrc=aw.ds
Osteocare tablets	Bone health	Until end of trial	If start date given, but no stop date, assume end of trial	2	Daily	2	11.2	0.12	https://www.boots.com/vitabiotics-osteocare-original---90-tablets-10268020?srsltid=AfmBOorW9LwAnhNbpbUOMi_ZLOCeMZ8pNZxjxJrCxJELIGFXD_tYOsmY
Osteoguard tablets	Bone health	Until end of trial	If start date given, but no stop date, assume end of trial	1	Daily	1	13.5	0.45	https://www.healthstuff.co.uk/en/gb/Lamberts-Osteoguard---90-Tabs/s-1354-1722.aspx?PartnerID=148&utm_source=google&utm_medium=cpc&gad_source=1&gclid=Cj0KCQjw1Yy5BhD-ARIsAI0RbXYGZzoF5_4GM6MkA3EBNkqIuU8ahgloFsru2FRnniY8

									w32gMPyjl1MaApjiEALw_wcB
Osteopathy	Chsc treatment	3	One off cost	0	Daily	1	55	55	https://www.nhs.uk/conditions/osteopathy/ average between 45 and 65
Pain killers (undefined)	Pain relief	2	One off cost	500mg four times	Daily	4	0.49	0.02	Paracetamol 500mg tablets / Packsize 32 (eMIT database)
Pomi-T	Supplement	Until end of trial	If start date given, but no stop date, assume end of trial	1	Daily	1	24.6	0.41	https://www.amazon.co.uk/Pomi-T-60caps-by-Nature-Medical/dp/B01M66FN5J/ref=pd_vtp_d_sccl_2_1/260-9014012-8105568?pd_rd_w=z6eBu&content-id=amzn1.sym.7b03b296-ca7e-42c7-b279-dea680a9c6a4&pf_rd_p=7b03b296-ca7e-42c7-b279-dea680a9c6a4&pf_rd_r=VK45D3KGGTMMXWNBNF5C&pd_rd_wg=hMFHv&pd_rd_r=e35e2d33-5552-48bb-99b8-b6a8c5e23795&pd_rd_i=B01M66FN5J&psc=1
Prebiotics + probiotics	Supplement	Until end of trial	If start date given, but no stop date,	1	Daily	1	22	0.73	https://www.boots.com/boots-synbiotics-pre--pro-gut-blend-

			assume end of trial						immunity-support-30-capsules-10278073?srsltid=AfmBOop_A4lQznEamQFchMo9vTM4jEaUHNUr8ebVqxvGH9TKru9I1tmM
Prednisolone	Steroids	7	One off cost	2.5mg	Daily	0.5	0.41	0.01	Prednisolone 5mg tablets / Packsize 28 (eMIT database)
Probiotics	Supplement	Until end of trial	If start date given, but no stop date, assume end of trial	1	Daily	1	26.95	0.9	https://www.boots.com/vitabiotics-probiotis-extra-strength-30-capsules-10336345?cm_mmc=bmm-buk-google-ppc-_-PLAs_HeroCompare-_--_-pmax_health__gg_shopping__hc-vitssupps___pmedia&gad_source=1&gclid=Cj0KCQjw1Yy5BhD-ARIsAI0RbXZKtnHzWmP6dmULc-GQMvt_VZ0Ge7gPKAN6dtf8DGYLqd_HOOG9XbEaApnkEALw_wcB&gclsrc=aw.ds
Ratanhia supplement	Mouthcare	One off	One off cost	1	One-off	1	13.13	13.13	https://www.beautyflash.co.uk/weleda-duo-ratanhia-mouthcare?gad_source=

									1&gclid=Cj0KCQjw1Yy5BhD-ARIsAI0RbXYfK5uLM52gDJqr_lEoNFdHWkBZ6acEm_9ZJnAQuzGQ0Q53JgtHp5oaAha5EALw_wcB
Red clover	Menopausal symptoms treatment	84	One off cost	1	Daily	1	16	0.53	https://www.boots.com/red-clover-isoflavones-30-tablets-10270060?cm_mmc=bmm-buk-google-ppc-_-PLAs_HeroCompare-_--_-pmax_health__gg_shopping__hc-vitssupps___pmedia&gad_source=1&gclid=Cj0KCQjw1Yy5BhD-ARIsAI0RbXZl9EIoyJQu_4duO2uKSCH5J8G26sqsC_G_NioU3kOPYTUFRZea9T4aAiO_EALw_wcB&gclsrc=aw.ds
Reiki	Chsc treatment	3	One off cost	0	Daily	1	56.06	56.06	Physiotherapist face to face (band 6) PSSRU
Rescue Remedy product	Supplement	One off	One off cost	1	One-off	1	12.5	12.5	https://www.boots.com/bach-rescue-remedy-dropper-20ml
Rhus toxicodendrum	Supplement	7	One off cost	2	Daily	2	9.28	0.07	https://www.natureshealthbox.co.uk/products/w

									eleda-rhus-tox-30c-tablets-125?variant=44812392595723&currency=GBP&utm_medium=product_sync&utm_source=google&utm_content=sag_organic&utm_campaign=sag_organic&utm_source=google&utm_campaign=21367761288&utm_medium=cpc&utm_content=&utm_term=&gad_source=1&gclid=CjwKCAjw-JG5BhBZEiwAt7JR6_JS8LGpCu-wwfCXIhoqTgjYUtI8l6a9DwQFoGD8iEWmh3NoilmHtxoCzYAQAvD_BwE
Rose hips	Supplement	90	One off cost	2	Daily	2	12.4	0.41	https://www.boots.com/jointace-rose-hip-and-msm-30-tablets-10077562
Sage tablets	Supplement	120	One off cost	1	Daily	1	15.99	0.53	https://www.boots.com/a-vogel-menoforce-sage-30-tablets-10117577?srsltid=AfmBOopiG0p_hkmMEPJt3JegTeFN-Cw0nBJyofwGULRb8KKv3jvKkw14

Sage tea	Drink	One off	One off cost	1	One-off	1	5.95	5.95	https://www.vitabiotics.com/products/ultra-sage-tablets?variant=29112666095685&network=google_x&placement=&keyword=&device=&campaignid=18064843341&adgroupid=&creativeid=&saf_src=google_x&saf_pt=&saf_kw=&saf_dv=&saf_cam=18064843341&saf_grp=&saf_ad=&account_id=6427961420&saf_acc=6427961420&gad_source=1&gclid=CjwKCAjw-JG5BhBZEiwAt7JR6yzeFnzWbXhB9Nt9g5E4GfckGbn92PCeCPruFurGvAlkTI9D_dX5MRoC77QQAvD_BwE
Sea kelp	Supplement	Until end of trial	If start date given, but no stop date, assume end of trial	1	Daily	1	9.99	0.05	https://www.superdrug.com/health/vitamins-supplements/minerals/prowise-sea-kelp-2000mg-200-vegan-tablets/p/mp-00026393?gclsrc=aw.ds&gclsrc=aw.ds&gad_source=1&gclid=Cj0KCQjw1Yy5BhD-ARIsAI0RbXbCXvCRG-qz15Aei-

									rlZ9cOtjiHEieg4pKNvjug5oa6PoCB1lKGdSMaAjWMEALw_wcB
Selenium	Supplement	180	One off cost	1	Daily	1	7.8	0.13	https://www.boots.com/boots-selenium-with-vitamins-a-c-and-e-60-tablets-10274976
Senna tablets	Constipation	7	One off cost	2	Daily	2	8	0.08	https://www.boots.com/care-senna-7-5mg-tablets-12-years-plus-100-tablets-10323707?srsltid=AfmBOooFGAcr1cLFzo9oNbPrQlJQ12tzsTpJdwsZ4TI6ZUo7ZuLlCg3z
Skin Nail Hair formula Boots	Supplement	Until end of trial	If start date given, but no stop date, assume end of trial	1	Daily	1	14.5	0.16	https://www.boots.com/boots-beauty-formula-skin-hair-nails-90-tablets-10319613?srsltid=AfmBOoojPNl_yq8pVEVlGqsHOsvNm3FyEk0V0bZFrCh-kN_L1eZY5XN2
Sleeping products	Sleeping treatment	14	One off cost	2	Daily	2	6.2	0.21	https://www.boots.com/wcsstore/ExtendedSitesCatalogAssetStore/Attachment%2FProduct_PatientLeaflets%2FPIL.30105.latest_Nytol_10087223.pdf

Soya product	Drink	One off	One off cost	1	One-off	1	2	2	https://www.tesco.com/groceries/en-GB/products/253178655?_gl=1*12blysi*_up*MQ..*_ga*ODg2NzY5Njk2LjE3MzA0NjAyNDQ.*_ga_33B19D36CY*MTczMDQ2MDI0NC4xLjAuMTczMDQ2MDI0NC4wLjAuOTIxMjI3MTA0
St John's wort	Supplement	84	One off cost	1	Daily	1	9.3	0.31	https://www.boots.com/boots-pharmaceuticals-st-johns-wort-tablets-30-x-250-mg?cm_mmc=bmm-buk-google-ppc-_-LIAs-_--_-pmax_health__gg_shopping__lia___pmedia&gad_source=1&gclid=CjwKCAjw-JG5BhBZEiwAt7JR62ZLzImYIABZpPzd6Lcp1SCUlO3CJMbFJHV_dsphCM6G0RdIkVD2tBoCHrcQAvD_BwE&gclsrc=aw.ds
Starflower oil	Supplement	42	One off cost	1	Daily	1	7.5	0.25	https://www.boots.com/boots-starflower-oil-1000-mg-30-capsules-10274984?cm_mmc=bmm-buk-google-ppc-_-PLAs_HeroCompare-_-

									-_-pmax_health__gg_shopping__hc-vitssupps___pmedia&gad_source=1&gclid=CjwKCAjw-JG5BhBZEiwAt7JR69cFILwMrORjK_bOS2QTq3rA6zscwlLXwRm-nq180-xYui64InPMARoClqIQAvD_BwE&gclsrc=aw.ds
Turmer + black pepper supplement	Supplement	180	One off cost	1	Daily	1	12.5	0.21	https://www.boots.com/boots-naturals-turmeric--black-pepper-60-tablets-10275605?srsltid=AfmBOordlFcmHH12h4Ak7aCoPHN9h5JcvKGbgtERfiq4Yr0KX4Qs8f2t
Turmeric + curcumin supplement	Supplement	180	One off cost	3	Daily	3	20.99	0.23	https://www.hollandandbarrett.com/shop/product/turmeric-vitality-organic-turmeric-curcumin95-capsules-60039647?skuid=039647&utm_campaign=shopping&utm_medium=cpc&utm_source=google&&gad_source=1&gclid=CjwKCAjw-JG5BhBZEiwAt7JR656YJxN1giX5kMJ8RmJu

									ofiYjrknWRXwkrgJMPBUCbGG2lpiVR7fWxoCPxMQAvD_BwE&gclsrc=aw.ds
Turmeric supplement	Supplement	180	One off cost	1	Daily	1	16.99	0.57	https://www.boots.com/boots-joint-assure-turmeric-30-capsules-30-tablets-10294898
Vaginal dilator	Device	One off	One off cost	1	One-off	1	69.99	69.99	https://www.boots.com/lovehoney-health-silicone-dilator-set-10326858?srsltid=AfmBOopB6xW5YbvMoDGqfvjzX8JmYsR5Up61gei66VZ5zm4ZVLyg77-A
Vagisil	Cream	One off	One off cost	1	One-off	1	5.49	5.49	https://www.boots.com/vagisil-medicated-creme-30g-10007183
Valerian root	Anxiety treatment	14	One off cost	6	Daily	6	9.39	0.056	https://www.boots.com/kalms-day-valerian-root-extract-25-99mg-tablets-168-tablets-10334613
Vitamin C supplement (undefined)	Supplement	Until end of trial	If start date given, but no stop date, assume end of trial	1	Daily	1	4.5	0.02	https://www.boots.com/boots-everyday-vitamin-c-240-tablets-10327278?cm_mmc=bmm-buk-google-ppc-_-PLAs_HeroCompare-_--_-pmax_health__gg_shop

									ping__hc-vitssupps___pmedia&gad_source=1&gclid=CjwKCAjw-JG5BhBZEiwAt7JR653bKx9f1vDeBX8Jol4E3NkVoKIlNy7TgPadWt1uirr5l2p8_8Fe0BoCD7QQAvD_BwE&gclsrc=aw.ds
Vitamin D3 + K2	Supplement	Until end of trial	If start date given, but no stop date, assume end of trial	1	Daily	1	13.47	0.22	https://www.boots.com/osavi-vitamin-d3-and-k2-4000iu-and-150mcg-60-softgels-10343981?srsltid=AfmBOooaONh7UDsHIu3D3reyheQn3m9fs09cNHG-0kbyKq78BNcBTG8b
Vitamin E supplement (undefined)	Supplement	Until end of trial	If start date given, but no stop date, assume end of trial	1	Daily	1	8	0.09	https://www.boots.com/boots-skin-formula-vitamin-e-90-capsules-10319614?cm_mmc=bmm-buk-google-ppc-_-PLAs_HeroCompare-_--_-pmax_health__gg_shopping__hc-vitssupps___pmedia&gad_source=1&gclid=CjwKCAjw-JG5BhBZEiwAt7JR6xOlyY4aJ2T4poCcwioIUREMtCJdFihBY417C2r

									mBLmaGxR2IUbAgxoCteUQAvD_BwE&gclsrc=aw.ds
Vitamin K2 supplement	Supplement	Until end of trial	If start date given, but no stop date, assume end of trial	1	Daily	1	22.14	0.18	https://www.boots.com/osavi-vitamin-k2-mk-7-100mcg-120-softgels-10348833?cm_mmc=bmm-buk-google-ppc-_-PLAs_HeroCompare-_--_-pmax_health__gg_shopping__hc-vitssupps___pmedia&gad_source=1&gclid=CjwKCAjw-JG5BhBZEiwAt7JR69SSrS3l6piyHOsEZIYUhJ6kN-XS3fPs30VFIEGbw1ac2KZDSL18IBoCFncQAvD_BwE&gclsrc=aw.ds
Voltarol	Cream	One off	One off cost	1	One-off	1	8.5	8.5	https://www.boots.com/voltarol-joint-pain-relief-gel---30g-10254311?srsltid=AfmBOoqvx5KPrStwr-gJVzSXeC9JbOyv8wWS-dfi87FQd0t-aEanDBiC
Wellwoman 50+ tablets	Supplement	Until end of trial	If start date given, but no stop date,	1	Daily	1	10.5	0.35	https://www.boots.com/wellwoman-50-plus-30-tablets-

			assume end of trial						10072511?srsltid=AfmBOopEsgLwgPAk2i7eH25-xq_dhE0oOf2fQQTkoyaJejQJt3luAEQo
Wheatgrass supplement	Supplement	Until end of trial	If start date given, but no stop date, assume end of trial	1	Daily	1	5.99	0.18	https://www.nutriseed.co.uk/products/wheatgrass-by-nutriseed?utm_source=google&utm_medium=surfaces&wickedsource=google&wickedid=CjwKCAjw-JG5BhBZEiwAt7JR65wHr47wcTChokmP55ugZebKuw2cMQvFury8-ca3dItb14oW9MnmIxoCrg0QAvD_BwE&wickedid=688129760326&wv=3.1&nbt=nb%3Aadwords%3Ag%3A20947345833%3A15625282556 5%3A688129760326&nb_adtype=pla&nb_kwd=&nb_ti=pla-338739924937&nb_mi=109261234&nb_pc=online&nb_pi=shopify_GB_1512194757_8326395013&nb_ppi=338739924937&nb_placement=&nb_li_ms=&nb_lp_ms=&nb_fii=&nb_ap=&nb_mt=&tw_source=googl

									e&tw_adid=688129760326&tw_campaign=20947345833&gad_source=1&gclid=CjwKCAjw-JG5BhBZEiwAt7JR65wHr47wcTChokmP55ugZebKuw2cMQvFury8-ca3dItb14oW9MnmIxoCrg0QAvD_BwE
Zinc supplement	Supplement	Until end of trial	If start date given, but no stop date, assume end of trial	1	Daily	1	8.2	0.05	https://www.boots.com/boots-zinc-10mg-180-one-a-day-tablets-10131469?srsltid=AfmBOooVmpgScGAVU2STLiuNLAaLsvbdyF2w-fxyh7LbJkctCnDRmCqa

Supplemental Information file 3: Supplemental Results

Base-case analysis

Supplementary Table 3. 1 Proportion and percentage of patient questionnaires expected, returned and total missing, dead, withdrawn and missing at random questionnaires at each timepoint for annual mammography arm

	Mammogram follow-up frequency					
	Arm 1: annual					
Timepoint	Received (n)	Expected (n)	Total Missing (%)[1]	Dead (%)[2]	Withdrawn (%)[2]	Missing at random (%)[2]
3	2,499	2,618	119 (5)	0 (0)	0 (0)	119 (100)
4	2,337	2,618	281 (11)	6 (2)	14 (5)	261 (93)
5	2,157	2,618	461 (18)	24 (5)	34 (7)	403 (87)
6	2,010	2,618	608 (23)	54 (9)	55 (9)	499 (82)
7	1,918	2,618	700 (27)	81 (12)	76 (11)	543 (78)
8	1,805	2,618	813 (31)	125 (15)	97 (12)	591 (73)
9	1,451	2,618	1,167 (45)	151 (13)	116 (10)	900 (77)

[1] Percentage of total missing questionnaires is estimated based on the number of total missing and the number of expected questionnaires at each timepoint

[2] Percentage of dead, withdrawn patients or missing at random questionnaires are estimated based on the number of dead, withdrawn patients or missing at random (respectively) and the number of missing questionnaires at each timepoint

Supplementary Table 3. 2 Proportion and percentage of patient questionnaires expected, returned and total missing, dead, withdrawn patients, and missing by design and missing at random questionnaires at each timepoint for less frequent mammogram arm

	Mammogram follow-up frequency						
	Arm 2: less frequent (two- yearly and three-yearly)						
Timepoint	Received (n)	Expected (n)*	Total Missing (%)[1]	Missing by design (%)[2]	Dead (%)[2]	Withdrawn (%)[2]	Missing at random (%)[2]
3	2,465	2,617	152 (6)	0 (0)	0 (0)	0 (0)	152 (100)
4	155	2,617	2,462 (94)	2,387 (97)	20 (1)	55 (2)	0 (0)
5	1,636	2,617	981 (37)	452 (46)	41 (4)	85 (9)	403 (41)
6	369	2,617	2,248 (86)	1,940 (86)	65 (3)	108 (5)	135 (6)
7	1,431	2,617	1,186 (45)	433 (37)	98 (8)	127 (11)	528 (45)
8	21	2,617	2,596 (99)	2,324 (90)	128 (5)	144 (6)	0 (0)
9	1,330	2,617	1,287 (49)	0 (0)	150 (12)	145 (11)	992 (77)

[1] Percentage of total missing questionnaires is estimated based on the number of total missing and the number of expected questionnaires at each timepoint

[2] Percentage of dead, withdrawn patients, missing by design or missing at random are estimated based on the number of dead, withdrawn patients, missing by design or missing at random questionnaires (respectively) and the number of missing questionnaires at each timepoint

*"Expected" represents the total number of participants in each arm at that timepoint. In Arm 2 (less frequent surveillance), participants were not required to complete questionnaires at certain timepoints due to the study design

Supplementary Table 3. 3 Proportion and percentage of patient questionnaires expected, returned and total missing, dead, withdrawn patients, and missing by design and missing at random questionnaires at each timepoint for less frequent mammogram arm (two-yearly only)

	Mammogram follow-up frequency						
	Arm 2: less frequent (two -yearly only)						
Timepoint	Received (n)	Expected (n)*	Total Missing (%)[1]	Missing by design (%)[2]	Dead (%)[2]	Withdrawn (%)[2]	Missing at random (%)[2]
3	1,997	2,099	102 (5)	0 (0)	0 (0)	0 (0)	102 (100)
4	125	2,099	1,974 (94)	1,919 (97)	12 (1)	43 (2)	0 (0)
5	1,612	2,099	487 (23)	0 (0)	25 (5)	59 (12)	403 (83)
6	44	2,099	2,055 (98)	1,940 (94)	43 (2)	72 (4)	0 (0)
7	1,421	2,099	678 (32)	0 (0)	62 (9)	88 (13)	528 (78)
8	19	2,099	2,080 (99)	1,898 (91)	82 (4)	100 (5)	0 (0)
9	1,095	2,099	1,004 (48)	0 (0)	95 (9)	100 (10)	809 (81)

[1] Percentage of total missing questionnaires is estimated based on the number of total missing and the number of expected questionnaires at each timepoint

[2] Percentage of dead, withdrawn patients, missing by design or missing at random are estimated based on the number of dead, withdrawn patients, missing by design or missing at random questionnaires (respectively) and the number of missing questionnaires at each timepoint

*"Expected" represents the total number of participants in each arm at that timepoint. In Arm 2 (less frequent surveillance), participants were not required to complete questionnaires at certain timepoints due to the study design

Supplementary Table 3. 4 Proportion and percentage of patient questionnaires expected, returned and total missing, dead, withdrawn patients, and missing by design and missing at random questionnaires at each timepoint for less frequent mammogram arm (three-yearly only)

	Mammogram follow-up frequency						
	Arm 2: less frequent (three-yearly only)						
Timepoint	Received (n)	Expected (n)*	Total Missing (%)[1]	Missing by design (%)[2]	Dead (%)[2]	Withdrawn (%)[2]	Missing at random (%)[2]
3	468	518	50 (10)	0 (0)	0 (0)	0 (0)	50 (100)
4	30	518	488 (94)	468 (96)	8 (2)	12 (2)	0 (0)
5	24	518	494 (95)	452 (91)	16 (3)	26 (5)	0 (0)
6	325	518	193 (37)	0 (0)	22 (11)	36 (19)	135 (70)
7	10	518	508 (98)	433 (85)	36 (7)	39 (8)	0 (0)
8	2	518	516 (100)	426 (83)	46 (9)	44 (9)	0 (0)
9	235	518	283 (55)	0 (0)	55 (19)	45 (16)	183 (65)

[1] Percentage of total missing questionnaires is estimated based on the number of total missing and the number of expected questionnaires at each timepoint

[2] Percentage of dead, withdrawn patients, missing by design or missing at random are estimated based on the number of dead, withdrawn patients, missing by design or missing at random questionnaires (respectively) and the number of missing questionnaires at each timepoint

"Expected" represents the total number of participants in each arm at that timepoint. In Arm 2 (less frequent surveillance), participants were not required to complete questionnaires at certain timepoints due to the study design

Supplementary Table 3. 5 Proportion and percentage of EQ5D index scores expected, returned and total missing, dead, withdrawn and missing at random, missing due to one or more EQ5D index dimensions left incomplete at each timepoint for annual mammogram arm

	Mammogram follow-up frequency						
	Arm 1: annual						
Timepoint	Received (n)	Expected (n)	Total Missing (%)[1]	Missing due to one or more EQ5D index dimensions left incomplete (n)	Dead (%)[2]	Withdrawn (%)[2]	Missing at random (%)[2]
3	2,441	2,618	177 (7)	58	0 (0)	0 (0)	177 (100)
4	2,270	2,618	348 (13)	67	6 (2)	14 (4)	328 (94)
5	2,097	2,618	521 (20)	60	24 (5)	34 (7)	463 (89)
6	1,959	2,618	659 (25)	51	54 (8)	55 (8)	550 (83)
7	1,859	2,618	759 (29)	59	81 (11)	76 (10)	602 (79)
8	1,759	2,618	859 (33)	46	125 (15)	97 (11)	637 (74)
9	1,394	2,618	1,224 (47)	57	151 (12)	116 (9)	957 (78)

[1] Percentage of total missing EQ5D index is estimated based on the number of total missing and the number of expected EQ5D index at each timepoint

[2] Percentage of dead, withdrawn patients or missing at random are estimated based on the number of dead, withdrawn patients or missing at random (respectively) and the number of missing EQ5D index at each timepoint

Supplementary Table 3. 6 Proportion and percentage of EQ5D index scores expected, returned and total missing, dead, withdrawn patients, missing by design and missing at random EQ5D index scores, missing due to one or more EQ5D index dimensions left incomplete at each timepoint for less frequent mammogram arm

	Mammogram follow-up frequency							
	Arm 2: less frequent (two- yearly and three-yearly)							
Timepoint	Received (n)	Expected (n)*	Missing (%)[1]	Missing due to one or more EQ5D index dimensions left incomplete (n)	Missing by design (%)[2]	Dead (%)[2]	Withdrawn (%)[2]	Missing at random (%)[2]
3	2,417	2,617	200 (7)	48	0 (0)	0 (0)	0 (0)	200 (100)
4	146	2,617	2,471 (94)	9	2,387 (97)	20 (1)	55 (2)	9 (0)
5	1,590	2,617	1,027 (39)	46	452 (44)	41 (4)	85 (8)	449 (44)
6	362	2,617	2,255 (86)	7	1,940 (86)	65 (3)	108 (5)	142 (6)
7	1,387	2,617	1,230 (47)	44	433 (35)	98 (8)	127 (10)	572 (47)
8	21	2,617	2,596 (99)	0	2,324 (90)	128 (5)	144 (6)	0 (0)
9	1,294	2,617	1,323 (50)	36	0 (0)	150 (11)	145 (11)	1028 (78)

[1] Percentage of total missing EQ5D index scores is estimated based on the number of total missing and the number of expected EQ5D index scores at each timepoint

[2] Percentage of dead, withdrawn patients, missing by design or missing at random EQ5D index scores are estimated based on the number of dead, withdrawn patients, missing by design or missing at random EQ5D index scores (respectively) and the number of missing EQ5D index scores questionnaires at each timepoint

*"Expected" represents the total number of participants in each arm at that timepoint. In Arm 2 (less frequent surveillance), participants were not required to complete questionnaires and fill in EQ5D scores at certain timepoints due to the study design

Supplementary Table 3. 7 Proportion and percentage of EQ5D index scores expected, returned and total missing, dead, withdrawn patients, missing by design and missing at random EQ5D index scores, missing due to one or more EQ5D index dimensions left incomplete at each timepoint for less frequent mammogram arm (two-yearly only)

	Mammogram follow-up frequency							
	Arm 2: less frequent (two-yearly)							
Timepoint	Received (n)	Expected (n)*	Missing (%)[1]	Missing due to one or more EQ5D index dimensions left incomplete (n)	Missing by design (%)[2]	Dead (%)[2]	Withdrawn (%)[2]	Missing at random (%)[2]
3	1,959	2,099	140 (7)	38	0 (0)	0 (0)	0 (0)	140 (100)
4	116	2,099	1,983 (94)	9	1,919 (97)	12 (1)	43 (2)	9 (0)
5	1,566	2,099	533 (25)	46	0 (0)	25 (5)	59 (11)	449 (84)
6	44	2,099	2,055 (98)	0	1,940 (94)	43 (2)	72 (4)	0 (0)
7	1,377	2,099	722 (34)	44	0 (0)	62 (9)	88 (12)	572 (79)
8	19	2,099	2,080 (99)	0	1,898 (91)	82 (4)	100 (5)	0 (0)
9	1,066	2,099	1,033 (49)	29	0 (0)	95 (9)	100 (10)	838 (81)

[1] Percentage of total missing EQ5D index scores is estimated based on the number of total missing and the number of expected EQ5D index scores at each timepoint

[2] Percentage of dead, withdrawn patients, missing by design or missing at random EQ5D index scores are estimated based on the number of dead, withdrawn patients, missing by design or missing at random EQ5D index scores (respectively) and the number of missing EQ5D index scores questionnaires at each timepoint

*"Expected" represents the total number of participants in each arm at that timepoint. In Arm 2 (less frequent surveillance), participants were not required to complete questionnaires and fill in EQ5D scores at certain timepoints due to the study design

Supplementary Table 3. 8 Proportion and percentage of EQ5D index scores expected, returned and total missing, dead, withdrawn patients, missing by design and missing at random EQ5D index scores, missing due to one or more EQ5D index dimensions left incomplete at each timepoint for less frequent mammogram arm (three-yearly only)

	Mammogram follow-up frequency							
	Arm 2: less frequent (three-yearly)							
Timepoint	Received (n)	Expected (n)*	Missing (%)[1]	Missing due to one or more EQ5D index dimensions left incomplete (n)	Missing by design (%)[2]	Dead (%)[2]	Withdrawn (%)[2]	Missing at random (%)[2]
3	458	518	50 (12)	10	0 (0)	0 (0)	0 (0)	50 (100)
4	30	518	488 (94)	0	468 (96)	8 (2)	12 (2)	0 (0)
5	24	518	494 (95)	0	452 (91)	16 (3)	26 (5)	0 (0)
6	325	518	193 (37)	7	0 (0)	22 (11)	36 (18)	135 (70)
7	10	518	508 (98)	0	433 (85)	36 (7)	39 (8)	0 (0)
8	2	518	516 (100)	0	426 (83)	46 (9)	44 (9)	0 (0)
9	228	518	283 (56)	7	0 (0)	55 (19)	45 (16)	183 (65)

[1] Percentage of total missing EQ5D index scores is estimated based on the number of total missing and the number of expected EQ5D index scores at each timepoint

[2] Percentage of dead, withdrawn patients, missing by design or missing at random EQ5D index scores are estimated based on the number of dead, withdrawn patients, missing by design or missing at random EQ5D index scores (respectively) and the number of missing EQ5D index scores questionnaires at each timepoint

*"Expected" represents the total number of participants in each arm at that timepoint. In Arm 2 (less frequent surveillance), participants were not required to complete questionnaires and fill in EQ5D scores at certain timepoints due to the study design

Supplementary Table 3. 9 Proportion and percentage of EQ5D index scores returned, recurrence-free patients and EQ5D index score returned, recurrent patients and EQ5D index score returned at each timepoint for annual mammogram arm

	Arm 1: annual mammogram		
Timepoint	Received (n)	Disease free and received	Recurrence and returned
3	2,441	2,440 (100)	1 (0)
4	2,270	2,253 (99)	17 (1)
5	2,097	2,068 (99)	29 (1)
6	1,959	1,930 (99)	29 (1)
7	1,859	1,840 (99)	19 (1)
8	1,759	1,738 (99)	21 (1)
9	1,394	1,372 (98)	22 (2)

Supplementary Table 3. 10 Proportion and percentage of EQ5D index scores returned, recurrence-free patients and EQ5D index score returned, recurrent patients and EQ5D index score returned at each timepoint for less frequent mammogram arm

	Arm 2: less frequent mammogram (two- yearly and three-yearly)		
Timepoint	Received (n)	Disease free and received	Recurrence and returned
3	2,417	2,416 (100)	1 (0)
4	146	143 (98)	3 (2)
5	1,590	1,582 (99)	8 (1)
6	362	352 (97)	10 (3)
7	1,387	1,368 (99)	19 (1)
8	21	21 (100)	0 (0)
9	1,294	1,276 (99)	18 (1)

Supplementary Table 3. 11 Summary statistics of area under the curve contributions over time for imputed values (used in base-case analysis)

	**Mammogram follow-up frequency**						
	**Arm 1: annual**			**Arm 2: less frequent (two- yearly and three-yearly)**			
**Timepoint**	**Mean**	**Median**	**SE**	**Mean**	**Median**	**SE**	**Mean difference**
4	0.7889	0.8070	0.1816	0.7857	0.8075	0.1866	-0.0032
5	0.7789	0.8025	0.1965	0.7748	0.8025	0.2039	-0.0041
6	0.7700	0.8025	0.2128	0.7647	0.8025	0.2167	-0.0053
7	0.7605	0.8025	0.2287	0.7546	0.8025	0.2330	-0.0059
8	0.7439	0.8020	0.2487	0.7427	0.7940	0.2446	-0.0012

Supplementary Table 3. 12 Count of scheduled and unscheduled questionnaires for each arm at each timepoint

	Annual		Less frequent		Two-yearly		Three-yearly	
Timepoint	Scheduled	Unscheduled	Scheduled	Unscheduled	Scheduled	Unscheduled	Scheduled	Unscheduled
3	2,486	5	2,451	6	1,987	5	464	1
4	2,274	47	10	16	9	14	1	2
5	2,075	52	1,502	52	1,501	49	1	3
6	1,851	88	283	26	4	8	279	18
7	1,760	77	1,256	80	1,256	80	0	0
8	1,580	81	1	0	1	0	0	0
0	1,132	76	968	114	797	9	171	18

Supplementary Figure 3. 1 Base-case analysis cost-effectiveness acceptability curve

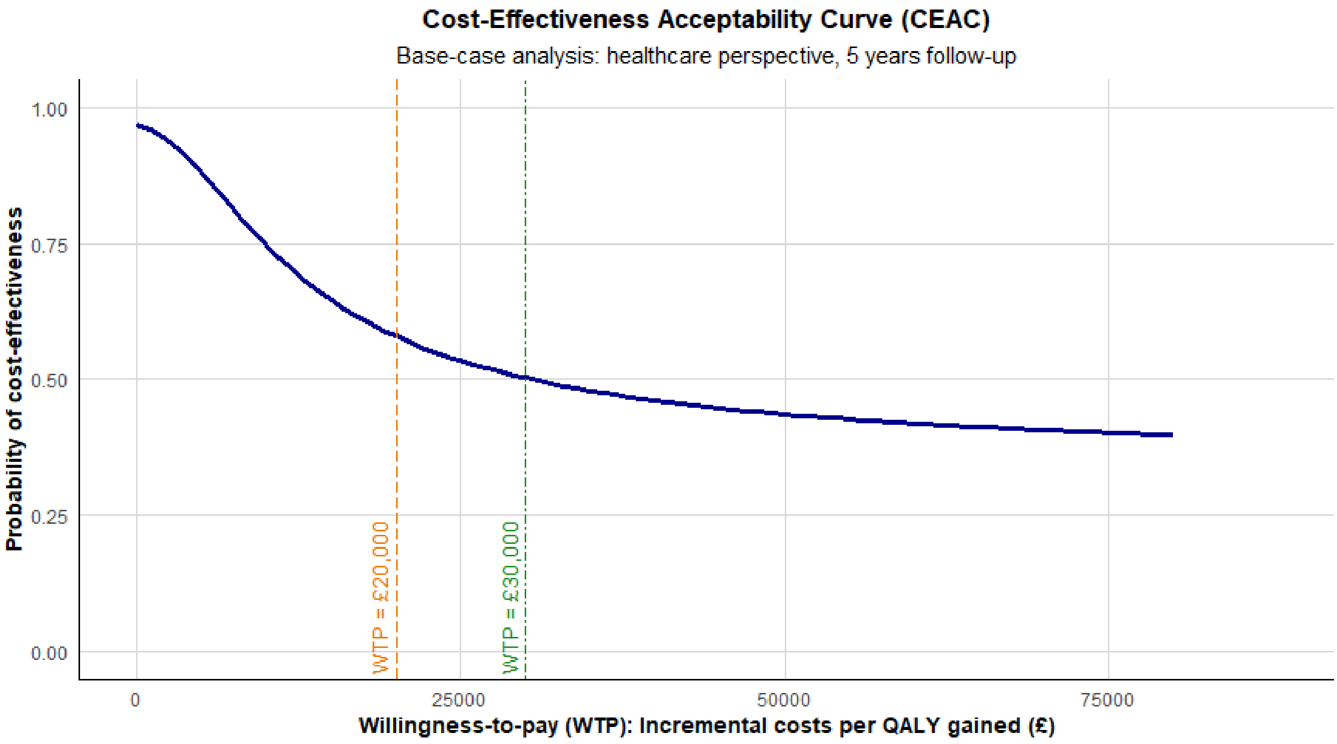

Secondary analyses

Supplementary Table 3. 13 Summary statistics for scenario and subgroup analyses.

Scenario analyses				
	Mean Incremental Costs (95% CI)	Mean Incremental QALYs (95% CI)	Mean INMB at £20,000 per QALY (95% CI)	Mean INMB at £30,000 per QALY (95% CI)
Base-case analysis	-£544 (-£1,114; £23)	-0.02 (-0.09; 0.04)	£187 (-£1,574; £2,027)	-£8 (-£2,498; £2,643)
Societal perspective	-£1,543 (-£2,416; -£669)	-0.02 (-0.09; 0.0)	£1,186 (-£824; £3,270)	£1008 (-£1,720; £3,861)
6-year follow-up	-£734 (-£1,525; £101)	-0.02 (-0.10; 0.06)	£339 (-£1,527; £2,142)	£142 (-£2,440; £2,637)
Outpatient costs excluded	-£310 (-£780; £162)	-0.02 (-0.09; 0.06)	-£47 (-£1,762; £1,731)	-£225 (-£2,692; £2,352)
Subgroup Analyses				
	Mean Incremental Costs (95% CI)	Mean Incremental QALYs (95% CI)	Mean INMB at £20,000 per QALY (95% CI)	Mean INMB at £30,000 per QALY (95% CI)
Surgery Type				
Wide Local Excision surgery (80%)	-£509 (-£28,638 ; £27,619)	-0.0001 (-2.6305 ; 2.6304)	£508 (-£59,148 ; £60,165)	£508 (-£83,269 ; £84,285)
Mastectomy surgery (20%)	-£687 (-£32,303 ; £30,929)	-0.0903 (-3.0169 ; 2.8363)	-£1,119 (-£67,645 ; £65,406)	-£2,023 (-£95,340 ; £91,295)
Type of disease				
DCIS only (13%)	-£1,154 (-£25,329 ; £23,021)	-0.1067 (-2.4458 ; 2.2323)	-£981 (-£53,640 ; £51,678)	-£2,048 (-£76,268 ; £72,171)
Invasive disease (87%)	-£456 (-£29,945 ; £29,034)	-0.0047 (-2.7378 ; 2.7285)	-£61,748 (£62,472 ; £51,678)	-£86,821 (£87,451 ; £72,171)
Age				
53-55 years old (8%)	-£886 (-£31,022 ; £29,251)	0.0243 (-2.6633 ; 2.7119)	£1,371 (-£60,252 ; £62,995)	£1,614 (£-84,461 ; £87,690)
55-75 years old (83%)	-£553 (-£29,095 ; £27,989)	-0.0092 (-2.6277 ; 2.6094)	£370 (-£59,273 ; £60,013)	£279 (-£8,3301 ; £83,858)

>75 years old (9%)	-£175 (-£30,526 ; £30,177)	-0.1335 (-3.325 ; 3.058)	-£2,496 (-£73,175 ; £68,183)	-£3,832 (-£104,272 ; £96,609)
Hormone therapy				
Therapy ongoing (73%)	-£342 (-£29,704 ; £29,019)	-0.0194 (-2.739 ; 2.7002)	-£45 (-£61,856 ; £61,766)	-£239 (-£86,949 ; £86,471)
Therapy stopped (5%)	-£2,541 (-£31,418 ; £26,337)	0.2164 (-2.7828 ; 3.2155)	£6,868 (-£59,704 ; £73,440)	£9,031 (-£85,463 ; £103,526)
Never started (22%)	-£748 (-27,935 ; £26,439)	-0.067 (-2.5758 ; 2.4418)	-£592 (-£57,661 ; £56,476)	-£1,262 (-£81,287 ; £78,762)
ER status				
ER-ve (11%)	-£6 (-£31,034 ; £31,021)	-0.0673 (-2.7242 ; 2.5897)	-£1,339 (-£62,873 ; £60,195)	-£2,012 (-£87,546 ; £83,522)
ER+ve (82%)	-£604 (-£29,552 ; £28,343)	-0.0128 (-2.7369 ; 2.7114)	£349 (-£61,346 ; £62,044)	£221 (-£86,478 ; £86,920)
Not done (7%)	-£790 (-£24,598 ; £23,018)	0.0248 (-2.2873 ; 2.3369)	£1,285 (-£50,726 ; £53,297)	£1,533 (-£71,803 ; £74,869)

Supplementary Figure 3. 2 Bootstrapped cost-effectiveness plane for secondary analysis (societal perspective, five-years follow-up period) with £20,000 (orange dashed line) and £30,000 (green dashed line) per QALY WTP thresholds and mean ICER (red square) based on 5,000 bootstrapped samples.

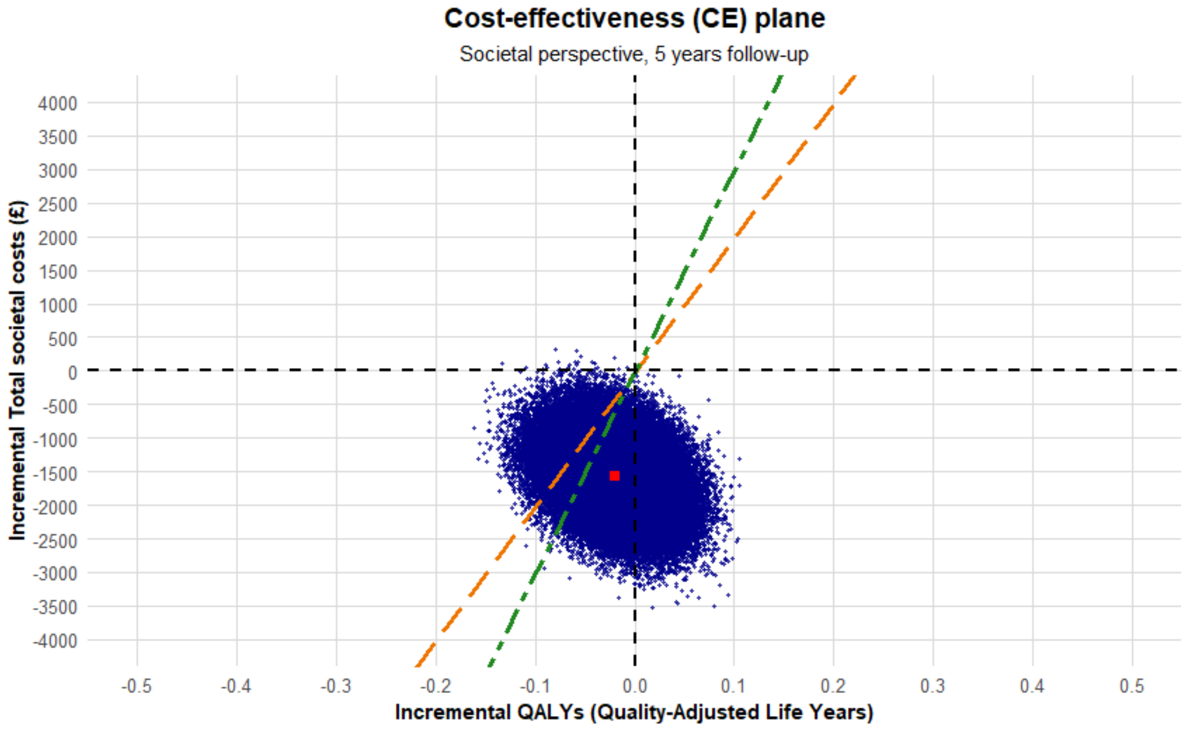

Supplemental Figure 3.2 illustrates the CE plane for the societal perspective over a 5-year follow-up, based on 5,000 bootstrapped estimates. Most points are below the x-axis, showing that the less frequent arm is cost-saving, and to the left of the y-axis, indicating a loss of QALYs compared to the annual arm. A large proportion of points fall below the £20,000 per QALY WTP threshold, indicating that the cost savings outweigh the value of the QALYs lost in most scenarios, making the less frequent arm cost-effective under this threshold. The societal perspective demonstrates larger cost savings for the less frequent arm compared to the healthcare perspective, reflecting additional reductions in non-healthcare costs like unpaid informal care and productivity losses.

Supplementary Figure 3. 3 Bootstrapped cost-effectiveness plane for sensitivity analysis 1 (healthcare perspective, six-years follow-up period) with £20,000 (orange dashed line) and

£30,000 (green dashed line) per QALY WTP thresholds and mean ICER (red square) based on 5,000 bootstrapped samples.

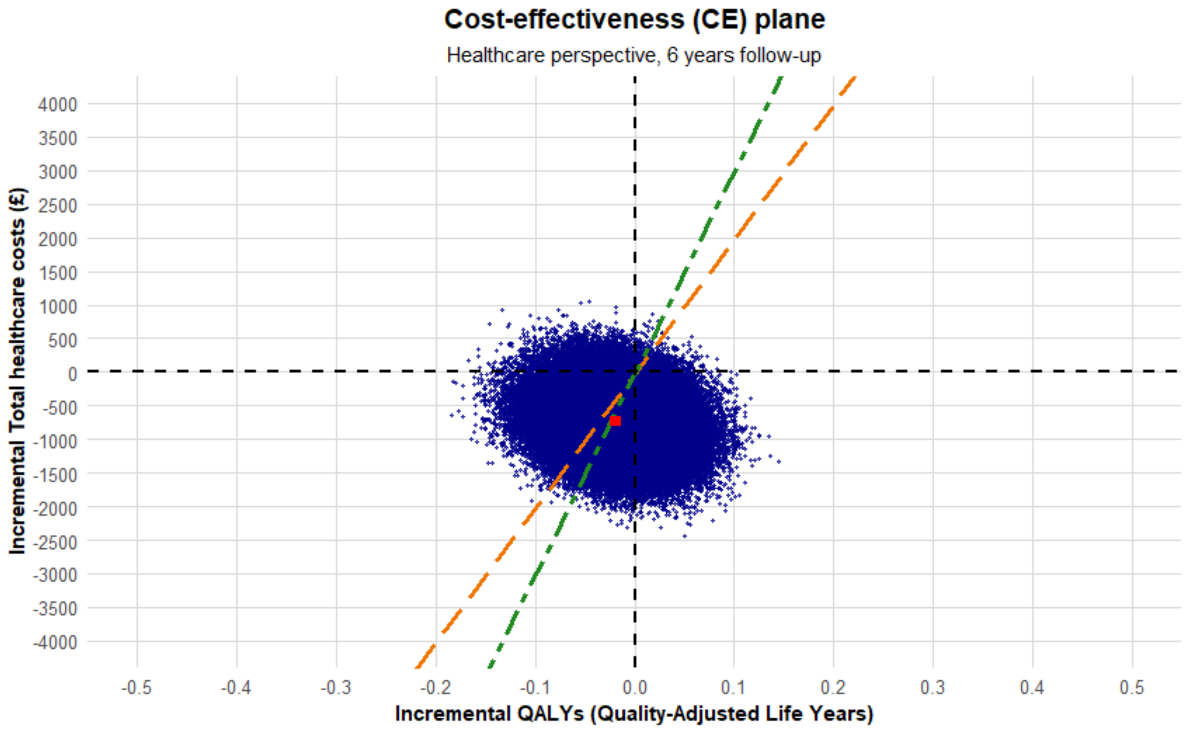

Supplemental Figure 3.3 illustrates the bootstrapped cost-effectiveness plane for the less frequent arm compared to the annual arm under a healthcare perspective with a 6-year follow-up. Most estimates fall below the £20,000 per QALY WTP threshold (orange dashed line), indicating that the less frequent arm is more cost-effective at this threshold. Some estimates also fall below the £30,000 per QALY WTP threshold (green dashed line), suggesting retained cost-effectiveness at higher thresholds.

The spread of points across all quadrants reflects substantial uncertainty in the estimates, with some scenarios showing higher costs or lower health outcomes for the less frequent arm. The clustering near the origin highlights small differences in costs and QALYs between the arms. Overall, the less frequent arm is likely cost-effective at £20,000 per QALY but shows diminishing certainty at £30,000.

Supplementary Figure 3. 4 Bootstrapped cost-effectiveness plane for sensitivity analysis 2 (healthcare perspective, five-years follow-up period, outpatient care costs set to £0) with £20,000

(orange dashed line) and £30,000 (green dashed line) per QALY WTP thresholds and mean ICER (red square) based on 5,000 bootstrapped samples.

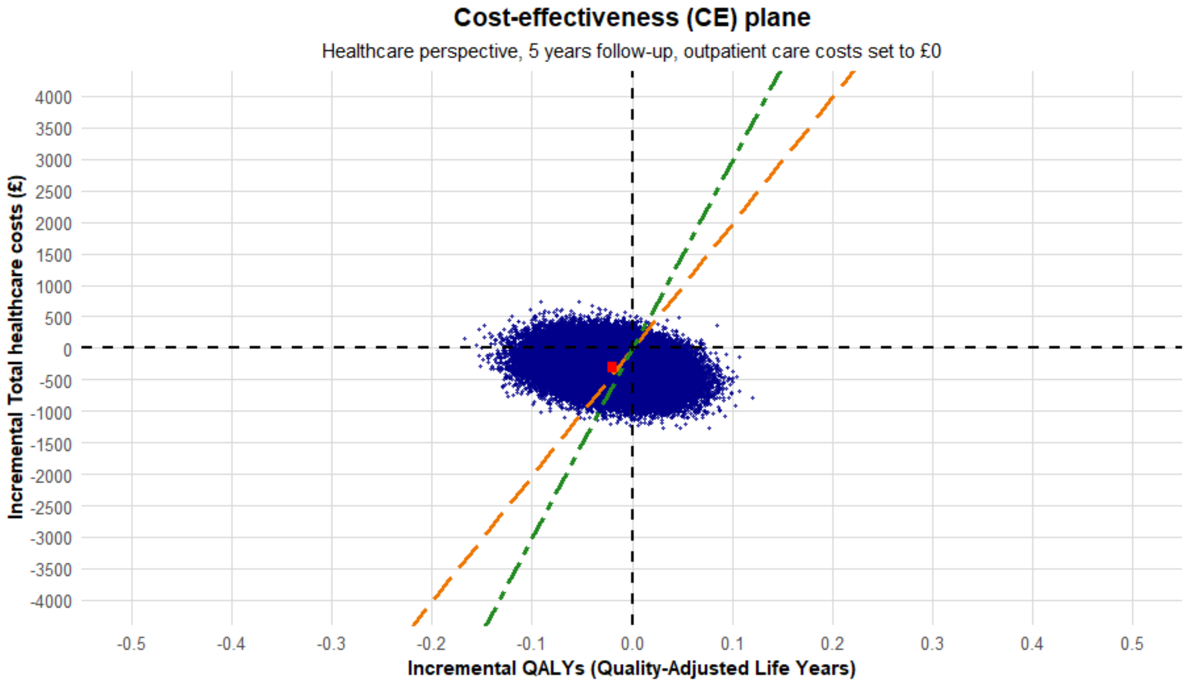

The CE plane illustrates substantial uncertainty in the cost-effectiveness estimates, with a notable proportion of simulations falling above the WTP thresholds. This suggests that, under the assumption of no difference in outpatient care costs, the less frequent arm appears to be less cost-effective compared to the annual arm.

Supplementary Figure 3. 5 Incremental Net Monetary Benefit (INMB) for varying surveillance mammogram costs at £20,000 per QALY WTP threshold (healthcare perspective)

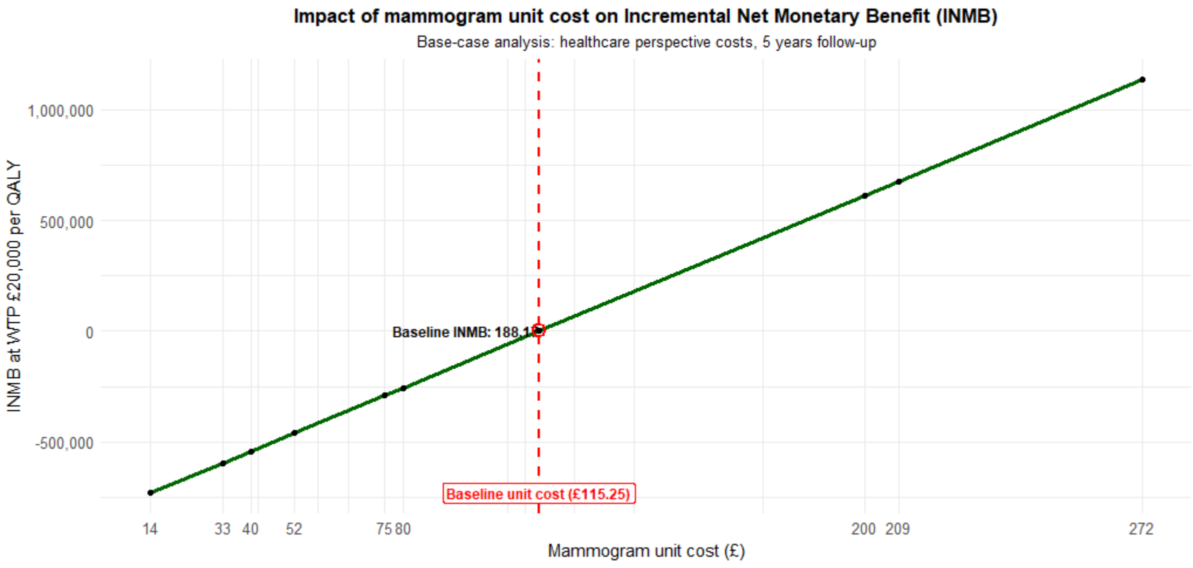

Supplementary Figure 3. 6 Bootstrapped Incremental Net Health Benefit (INHB) for varying surveillance mammogram costs at £20,000 per QALY WTP threshold (healthcare perspective, 5 years follow-up period), (n=5,000 bootstrapped samples)

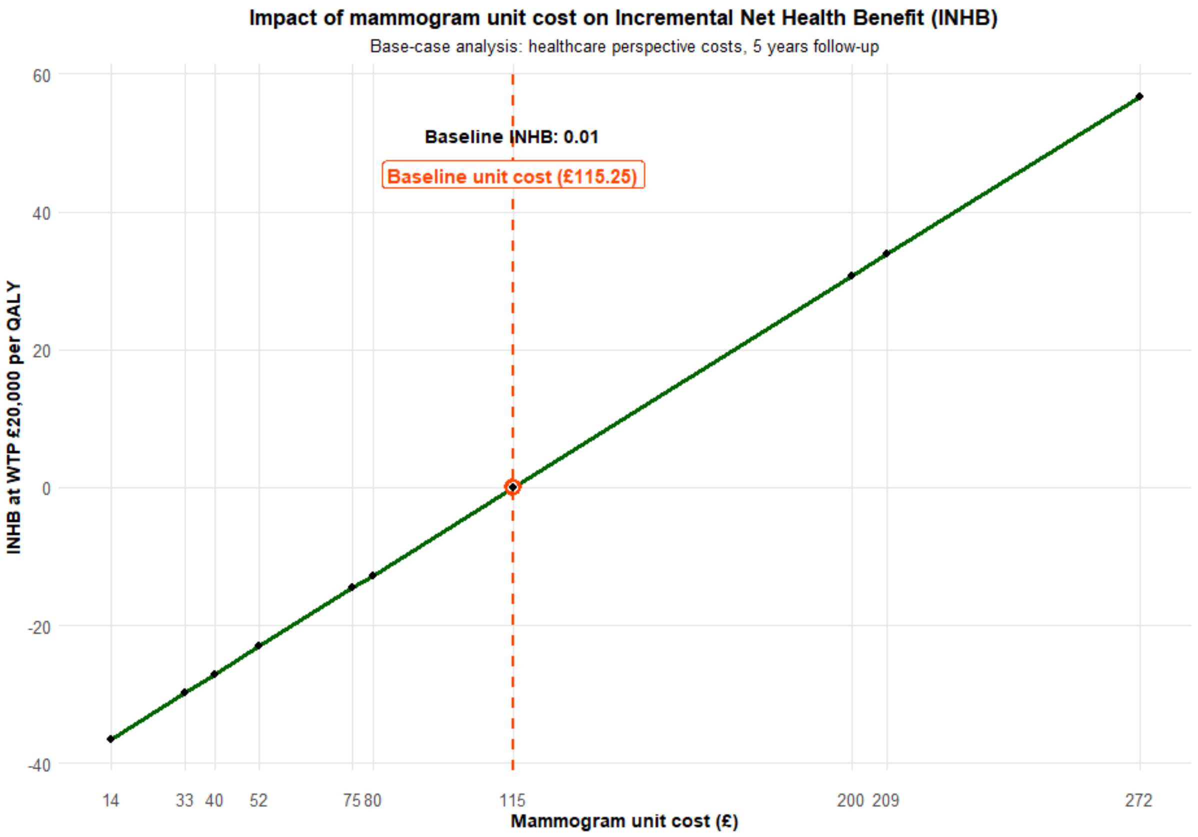

Budget impact analysis

Sensitivity and scenario analyses

Supplementary Table 3.14 summarises the annual cost savings to the NHS over a six-year period at varying mammogram unit cost and related per patient cost savings. At higher mammogram unit cost (£272), the cumulative cost savings reach £201,095,602 million, while at lower unit cost (£14.40), the savings decrease to £125,031,985 million.

Supplementary Table 3. 14 Scenario analysis budget impact analysis results: annual and cumulative cost savings to the NHS over a six-year horizon at varying mammogram unit cost and per patient cost savings

Mammogram unit cost	Per-patient cumulative cost savings	Per-patient yearly cost savings	2024	2025	2026	2027	2028	2029	Cumulative cost savings to the NHS
£272	£953	£159	£35,774,362	£32,697,342	£29,888,474	£32,162,565	£34,289,544	£36,283,314	£201,095,602
£209	£864	£144	£32,445,718	£29,655,000	£27,107,484	£29,169,982	£31,099,055	£32,907,313	£182,384,553
£200	£852	£142	£31,992,409	£29,240,682	£26,728,757	£28,762,440	£30,664,561	£32,447,555	£179,836,403
£80	£684	£114	£25,689,154	£23,479,581	£21,462,565	£23,095,564	£24,622,923	£26,054,626	£144,404,413
£75	£677	£113	£25,426,517	£23,239,535	£21,243,140	£22,859,444	£24,371,187	£25,788,253	£142,928,075
£52	£645	£107	£24,194,233	£22,113,242	£20,213,601	£21,751,572	£23,190,050	£24,538,438	£136,001,136
£40	£623	£105	£23,609,081	£21,578,419	£19,724,723	£21,225,497	£22,629,184	£23,944,960	£132,711,864
£33	£619	£103	£23,220,378	£21,223,150	£19,399,973	£20,876,037	£22,256,614	£23,550,727	£130,526,880
£14	£593	£99	£22,242,851	£20,329,702	£18,583,276	£19,997,202	£21,319,660	£22,559,294	£125,031,985

Supplemental Figure 3.7 illustrates the parameter-specific impact on cumulative cost savings to the NHS over a six-year period, with a baseline value of £185,866,317.71. The analysis shows that attrition rates for invasive cases and number of new diagnoses at stage 1 had the largest impact on cost savings, while yearly overall survival rates had a comparatively smaller influence.

Supplementary Figure 3. 7 Tornado plot: parameter impact on cumulative cost savings to the NHS over six-year period

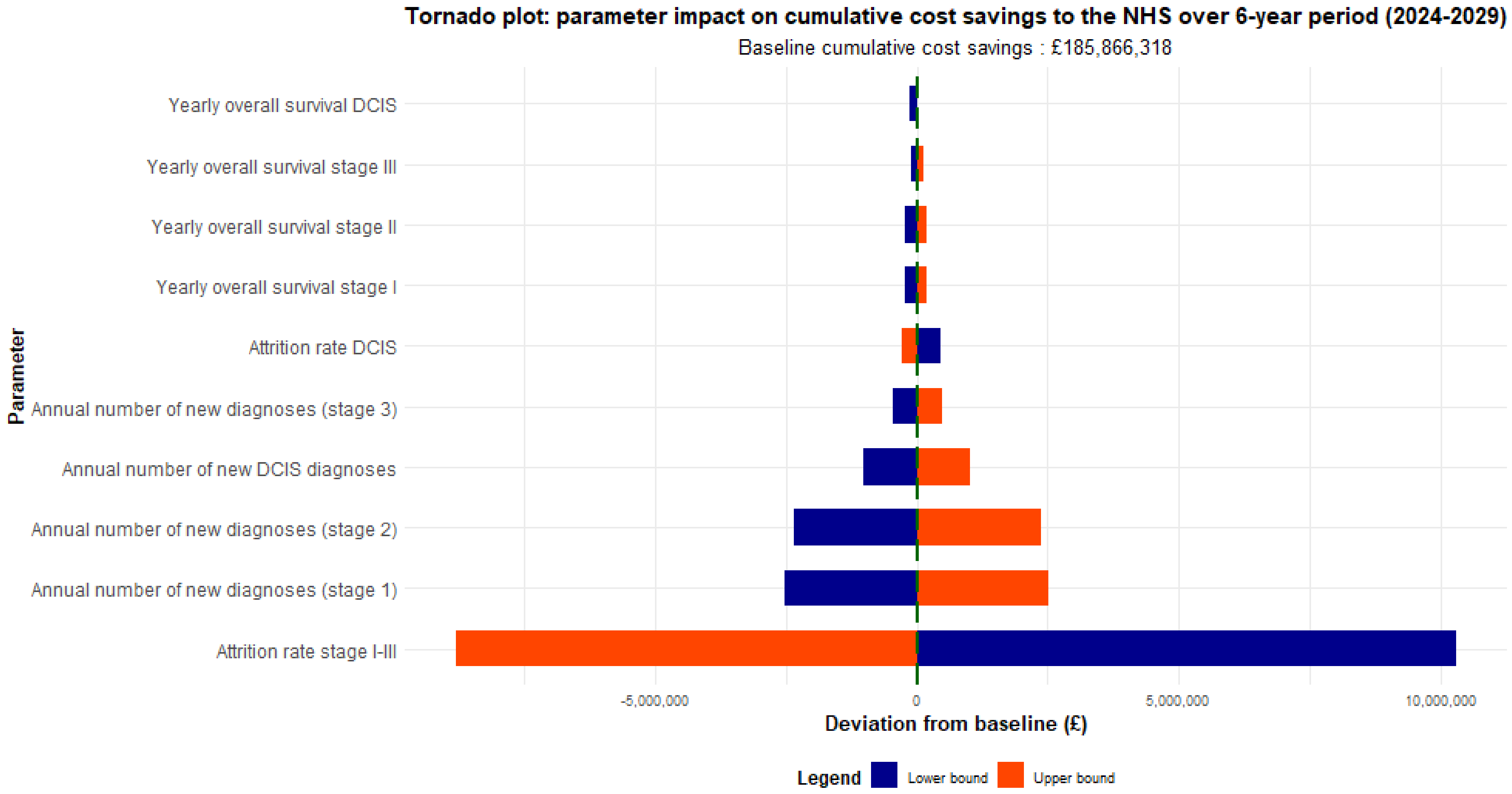

Supplementary Table 3.15 shows the expected numbers of mammogram for each arm and the number of mammograms saved each year when considering the total population contributing to cost-savings. The number of mammograms saved per year ranged from 87,788 in 2026 to 106,571 in 2029, reflecting changes in the surveillance population over time.

Supplementary Table 3. 15 Expected number of mammogram for each arm and number of mammograms saved each year

Year	Mammogram count annual arm (n)	Mammogram count less frequent arm (n)	Mammogram saved per year (n)
2024	225,239	120,163	105,076
2025	205,866	109,827	96,039
2026	188,181	100,393	87,788
2027	202,499	108,031	94,468
2028	215,891	115,175	100,715
2029	228,444	121,872	106,571
